# The Biosynthesis of Artemisinin (Qinghaosu) and the Phytochemistry of *Artemisia annua* L. (Qinghao)

**DOI:** 10.3390/molecules15117603

**Published:** 2010-10-28

**Authors:** Geoffrey D. Brown

**Affiliations:** Department of Chemistry, The University of Reading, Whiteknights, Reading, RG6 6AD, UK; E-Mail: g.d.brown@reading.ac.uk

**Keywords:** artemisinin, dihydroartemisinic acid, sesquiterpene, biosynthesis, *Artemisia annua*, phytochemistry, oxidation, allylic hydroperoxide

## Abstract

The Chinese medicinal plant *Artemisia annua* L. (Qinghao) is the only known source of the sesquiterpene artemisinin (Qinghaosu), which is used in the treatment of malaria. Artemisinin is a highly oxygenated sesquiterpene, containing a unique 1,2,4-trioxane ring structure, which is responsible for the antimalarial activity of this natural product. The phytochemistry of *A. annua* is dominated by both sesquiterpenoids and flavonoids, as is the case for many other plants in the Asteraceae family. However, *A. annua* is distinguished from the other members of the family both by the very large number of natural products which have been characterised to date (almost six hundred in total, including around fifty amorphane and cadinane sesquiterpenes), and by the highly oxygenated nature of many of the terpenoidal secondary metabolites. In addition, this species also contains an unusually large number of terpene allylic hydroperoxides and endoperoxides. This observation forms the basis of a proposal that the biogenesis of many of the highly oxygenated terpene metabolites from *A. annua* – including artemisinin itself – may proceed by spontaneous oxidation reactions of terpene precursors, which involve these highly reactive allyllic hydroperoxides as intermediates. Although several studies of the biosynthesis of artemisinin have been reported in the literature from the 1980s and early 1990s, the collective results from these studies were rather confusing because they implied that an unfeasibly large number of different sesquiterpenes could all function as direct precursors to artemisinin (and some of the experiments also appeared to contradict one another). As a result, the complete biosynthetic pathway to artemisinin could not be stated conclusively at the time. Fortunately, studies which have been published in the last decade are now providing a clearer picture of the biosynthetic pathways in *A. annua*. By synthesising some of the sesquiterpene natural products which have been proposed as biogenetic precursors to artemisinin in such a way that they incorporate a stable isotopic label, and then feeding these precursors to intact *A. annua* plants, it has now been possible to demonstrate that dihydroartemisinic acid is a late-stage precursor to artemisinin and that the closely related secondary metabolite, artemisinic acid, is not (this approach differs from all the previous studies, which used radio-isotopically labelled precursors that were fed to a plant homogenate or a cell-free preparation). Quite remarkably, feeding experiments with labeled dihydroartemisinic acid and artemisinic acid have resulted in incorporation of label into roughly half of all the amorphane and cadinane sesquiterpenes which were already known from phytochemical studies of *A. annua.* These findings strongly support the hypothesis that many of the highly oxygenated sesquiterpenoids from this species arise by oxidation reactions involving allylic hydroperoxides, which seem to be such a defining feature of the chemistry of *A. annua*. In the particular case of artemisinin, these *in vivo* results are also supported by *in vitro* studies, demonstrating explicitly that the biosynthesis of artemisinin proceeds via the tertiary allylic hydroperoxide, which is derived from oxidation of dihydroartemisinic acid. There is some evidence that the autoxidation of dihydroartemisinic acid to this tertiary allylic hydroperoxide is a non-enzymatic process within the plant, requiring only the presence of light; and, furthermore, that the series of spontaneous rearrangement reactions which then convert this allylic hydroperoxide to the 1,2,4-trioxane ring of artemisinin are also non-enzymatic in nature.

1. Introduction
  1.1 Malaria
  1.2 *Artemisia annua* (Qinghao)
  1.3 Artemisinin (Qinghaosu)
2. The Phytochemistry of *Artemisia annua* L. (Qinghao)
  2.1 Aliphatic hydrocarbons, alcohols, aldehydes and acids
  2.2 Aromatic alcohols, ketones and acids
  2.3 Phenylpropanoids
  2.4 Flavonoids
  2.5 Monoterpenoids
    2.5.1 Regular acyclic monoterpenes
    2.5.2 Irregular acyclic monoterpenes
    2.5.3 Monocyclic monoterpenes
    2.5.4 Bicyclic monoterpenes
  2.6 Sesquiterpenoids
    2.6.1 Farnesane sesquiterpenes
    2.6.2 Monocyclic sesquiterpenes
    2.6.3 Bicyclic sesquiterpenes
    2.6.4 Tricyclic sesquiterpenes
  2.7 Higher terpenoids
    2.7.1 Diterpenes
    2.7.2 Triterpenes and sterols
  2.8 Nitrogen-containing natural products
3. The Biosynthesis of artemisinin (Qinghaosu)
  3.1 Phase 1 (isopentenyl pyrophosphate to amorpha-4,11-diene)
  3.2 Phase 2 (amorpha-4,11-diene to dihydroartemisinic acid)
  3.3 Phase 3 (dihydroartemisinic acid to artemisinin)
4. Strategies for the production of artemisinin from *A. annua* and derived systems
  4.1 Plant breeding programmes
  4.2 Plant tissue culture
  4.3 Endophytic fungi
  4.4 Genetic engineering
5. Acknowledgements
6. References

## 1. Introduction

### 1.1. Malaria

Malaria is an infectious disease which has affected human beings since the dawn of recorded history. By the middle of the last century, however, many felt that malaria was on the retreat and that one day it might even be vanquished. Two factors were primarily responsible for this perceived reduction in the severity of the malarial threat. Firstly, the *Anopheles* mosquito, which transmits the disease to humans, could at last be controlled by widespread application of the insecticide DDT. Secondly, the *Plasmodium* parasite, which causes malaria (four members of the genus infect humans: *P. falciparum, P. vivax, P. malariae* and *P. ovale*), could also be effectively be controlled by the use of synthetic analogues of quinine (itself also a natural product, obtained from the bark of the cinchona tree), such as chloroquine, which had been developed before World War II.

By the 1960’s, however, malaria was back with a vengeance. The mosquitoes were developing resistance to DDT, which was soon to be banned in any case because of environmental concerns. The *Plasmodium falciparum* parasite, which is responsible for cerebral malaria, an often fatal complication, was also developing resistance to chloroquine. Thailand and South America were the first regions to be affected, but resistance to chloroquine soon spread to many other parts of the World. Nowadays, it is particularly serious in South East Asia. It was against this background of increasing resistance, and of the on-going wars in neighboring Cambodia and Vietnam, that the Chinese government began a major initiative to discover new antimalarials from plants used in Traditional Chinese Medicine (TCM). 

### 1.2. Artemisia annua (Qinghao)

The herb “Qinghao” first appeared in a book entitled “Wu Shi Er Bing Fang” (Prescriptions for Fifty-Two Ailments) more than two thousand years ago. The earliest reported use for Qinghao was for the treatment of haemorroids; but “Zhou Hou Bei Ji Fang” (Handbook of Prescriptions for Emergency Treatment), written in 340 AD, describes the use of Qinghao as a treatment for fevers [1]. The first text in which Qinghao might specifically be identified as a remedy for malaria is “Ben Cao Gang Mu” (1596) in which the herb is described as “a treatment for hot and cold due to intermittent fever illness”. These old pharmacopeias describe preparations in which the leaves (collected in summer or spring) are pounded with a pestle and mortar in order to express the “juice”. This procedure was, perhaps, intended to improve the recovery of essential oils from trichomes on the leaf surface, in which the active principal, artemisinin, is now thought be contained.

It is not entirely clear whether “Qinghao” which is referred to in these ancient texts is solely *Artemisia annua* L. [2]; and it has sometimes been suggested that the term may also have been used synonomously for *Artemisia apiacea* Hance [1]. In the modern pharmacopeia of the People’s Republic of China, however, Qinghao is now officially listed as the aerial parts of *A. annua* L. [3] (5g dried herb/1L water is suggested for the preparation of a decoction). In the TCM system, *A. annua* is cool in nature (yin) and is therefore suitable for treating “heat” syndromes (yang), such as malaria; other applications described for this cooling herb, such as the relief of symptoms of febrile diseases, tidal fever, low grade fever and summer heat stroke, are also consistent with this classification.

### 1.3. Artemisinin (Qinghaosu)

As has been noted, after a brief respite lasting only a few decades, malaria was once again on the increase in the 1960s. In 1967, the Chinese government launched a program to discover new antimalarial drugs based on a systematic investigation of indigenous plants used in TCM. 

When the Chinese scientists made infusions of *A. annua* with hot or boiling water, as described in the ancient texts, they observed no activity against mice infected with *Plasmodium berghei* (a rodent malarial parasite). However, thanks to the insight of Prof. Tu, cold ethereal extracts of *A. annua* were also tested and these did show encouraging activity, leading to the isolation of the active principle, artemisinin, in 1972 (the original reports of this new drug referred to “Qinghaosu”, meaning “principle from Qinghao” [4]). Nowadays, artemisinin is extracted from *A. annua* using diethyl ether, hexane, petroleum ether or even petrol as solvent [5].

At first sight, the lack of effectiveness for hot water extracts from *A. annua* appears to be at variance with the reports in the traditional pharmacopeias (in addition, we now know that artemisinin is virtually insoluble in water). One possible explanation is that the original recipes normally required that *A. annua* preparations be made in conjunction with other herbs (so-called “minister” or “guide” herbs). For example, in the herbal “Wen Bing Tiao Bian” (1798), an infusion of *A. annua* together with *Amyda sinensis*, *Rehmannia glutinosa*, *Anemarrhena asphodeloides* and *Paeonia suffructicosa* is described. It is quite possible that saponins, or other components from the latter three herbs which are capable of acting as detergents, might be able to assist the dissolution of artemisinin in water (*cf.* the suggestion in Section 2.4 that flavonoids, which are present both in *A. annua* and other herbs, may be able to potentiate the antimalarial effect of artemisinin in crude plant preparations). 

**Figure 1:**
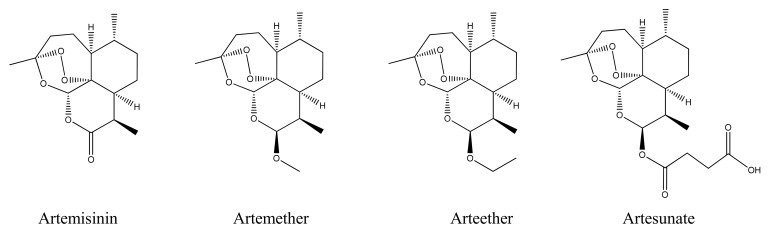
Artemisinin and its semi-synthetic derivatives, which are currently used in the treatment of malaria.

Clinical studies in the late 1970s with patients infected with *P. vivax* or *P. falciparum* demonstrated that artemisinin could kill the malarial parasite very quickly at the schizont stage of the parasite’s life cycle (*i.e.* while it infected the human red blood cell) and with no obvious side effects [6]. Most importantly, artemisinin was completely effective in the treatment of chloroquine-resistant *Falciparum* malaria [7]. The discovery of this new and potent antimalarial attracted the attention of the World Health Organization (WHO), which had the resources to develop artemisinin globally; and both *A. annua* and artemisinin soon became well known outside of China. However, the therapeutic value of artemisinin was limited by its low solubility in both oil and water, and this has lead to the development of semi-synthetic drugs with pharmacological properties superior to those of the parent [8]. The most important such derivatives are artemether, arteether and artesunate (Figure 1), which exhibit greater potency than artemisininin itself, as well as improved solubility, and favourable metabolic and hydrolytic stabilities. Formulations based on these drugs are now at the heart of the WHO’s global fight against malaria The entire world production of such semi-synthetic artemisinin derivatives is currently reliant on harvesting and extraction of artemisinin from *A. annua* plants, which is practiced on a multi-tonne scale in countries such as China and Vietnam.

Malaria is now the most serious infectious disease in the World, with at least 300 million cases reported every year. It is estimated to be responsible for up to 2 million deaths annually - mainly amongst children - with more than half of the deaths occurring amongst the poorest 20% of the World’s population. The importance of artemisinin has been founded on a continuing lack of resistance almost three decades after its introduction – although, very recently, reports of the emergence of resistance have indeed begun to appear [7,8] This slow onset of resistance may be a consequence of the unique mechanism of action for this drug, which is associated with the unusual endoperoxide group. It is thought that artemisinin becomes activated when its endoperoxide group comes into contact with Fe(II) in free haem groups, which have been liberated by the parasite’s digestion of the haemoglobin contained in the red blood cell. 

The appearance of resistance to artemisinin could be a devastating blow for many parts of South East Asia and Africa, where artemisinin-based drugs are currently the *only* effective treatment for malaria (resistance to the older generation of quinine-derived antimalarials having already become endemic to these regions). In an attempt to forestall the emergence of resistance, the WHO have been recommending that artemisnin should be taken in combination with another antimalarial drug – so-called Artemisinin Combination Therapy (ACT). This strategy is designed to slow the development of resistance, because during treatment with two drugs, the chance of a mutant emerging which is resistant to both is the product of the probabilities of resistance arising to either drug separately. It seems that, in the continuing absence of an effective malaria vaccine, the development of new antimalarial drugs – most likely derived from, or inspired by, artemisinin – will continue to be our primary weapon in the fight against malaria. 

## 2. The Phytochemistry of *Artemisia annua* L. (Qinghao)

*Artemisia annua* Linn. (sweet wormwood; Chinese wormwood) is a member of the Asteraceae family of plants (formerly Compositae). It is a native of China, originally found in the steppes of Chahar and Suiyuan Provinces, but is now widespread in many parts of the World, and is cultivated in countries such as Vietnam, Thailand, Burma, Madagascar, Malaysia, USA, Brazil, Australia (Tasmania), Holland, Switzerland, France and Finland. Most phytochemical investigations of *A. annua* have employed the aerial parts (leaves and/or stems - sometimes also including the flowers) although one report has concentrated on the seeds [9]. The phytochemistry of *A. annua* is dominated by terpenoids (in particular sesquiterpene lactones), flavonoids, coumarins and other shikimate metabolites, as is the case for many other members of the genus *Artemisia*. The sesquiterpene, artemisinin, is however unique to *A. annua* – it has been searched for on several occasions in other species in the genus *Artemisia*, always without success [10,11]. The artemisinin content from *A. annua* is highly variable, ranging anywhere between 0.01% and 1%, depending on variety, and can even be as high as 1.4% in some cultivated strains.

There have been several reviews of the phytochemistry of *A. annua* [12,13,14,15]. In this section, an attempt has been made to provide a comprehensive review of the original phytochemical literature concerning *A. annua* up to 2009. Almost six hundred secondary metabolites are described, which have been divided into eight sections (Section 2.1, Section 2.2, Section 2.3, Section 2.4, Section 2.5, Section 2.6, Section 2.7 and Section 2.8) and further sub-divided into more than forty Tables. The grouping of these natural products from *A. annua* is based on the (largely biogenetic) classification adopted by a standard reference work, the Dictionary of Natural Products [16]. Thus, the structurally most simple group of metabolites – derivatives of aliphatic hydrocarbons (branched, unbranched, saturated or unsaturated) with varying levels of oxidation at C-1 (alcohol, aldehyde, ketone, acid or ester) - are described first in Section 2.1. The next major group to be covered in Section 2.2 comprises derivatives of simple aromatic hydrocarbons. The phenylpropanoids, in which a C_3_ substituent is attached to the aromatic unit (C_6_), form a biosynthetically distinct group of aromatic metabolites, which is described in Section 2.3. The flavonoids, which are biosynthetically derived from this same C_6_C_3_ precursor, constitute the second largest group of metabolites from *A. annua*, and are described separately in Section 2.4. By far the largest group of metabolites from *A. annua* is the terpenoids, which are biosynthetically derived from a branched isoprenoid unit (C_5_). They have been further subdivided into monoterpenoids (C_10_; *i.e.* 2 × C_5_); sesquiterpenoids (C_15_ ; *i.e.* 3 × C_5_); and higher terpenoids (which include both diterpenes (C_20_), triterpenes (C_30_) and sterols (C_29_). These large groupings are covered in Section 2.5, Section 2.6 and Section 2.7, respectively. Finally, a very small number of miscellaneous nitrogen-containing natural products appear in Section 2.8.

The essential oil of *A. annua* was first studied as long ago as 1917 [17,18]. Researchers have continued to analyse the volatile constituents of this essential oil over the past century, primarily utilising gas chromatography (GC) and the hyphenated analytical technique of gas chromatography-mass spectrometry (GC-MS) [19,20,21,22,23,24,25,26,27,28,29,30,31,32,33,34,35]. GC/GC-MS studies of the essential oil of *A. annua* have been reported from countries as diverse as France [36], Finland [37], Hungary [38], Romania [39], Kazakhstan [40], Iran [41,42], India [43,44,45], China [46,47,48,49,50] and Vietnam [51]. A detailed study of the essential oils from Chinese and Vietnamese varieties of *A. annua* demonstrated that the Chinese variety contained predominantly artemisia ketone, while the Vietnamese oil was dominated by camphor and germacrene D [21] (others have confirmed the absence of artemisia ketone from Vietnamese oil) [52]. Interestingly, this difference was also reflected in the artemisinin content of Chinese and Vietnamese plants at 0.17% and 1.0% dry weight, respectively. Others have reported a similar variation in essential oil content between varieties of *A. annua* [53,54] and have concurred that differences in the essential oil composition should be ascribed to the existence of chemotypes (or chemical races) in this species [43] (see also Section 4.1).

GC-MS studies are particularly suited to the analysis of the more volatile components of the plant metabolome, such as the monoterpenes (Section 2.5) and some of the unfunctionalized sesquiterpene hydrocarbons which are reported in Section 2.6. The identification of a metabolite by GC-MS generally requires that its retention time and mass spectrum be matched with that of a known standard, which is recorded in a database. Using this thechnique, it is therefore possible to make a very rapid analysis of a large number of compounds employing a relatively small amount of plant material. Many of the simple aliphatic and aromatic metabolites which are reported in Section 2.1 and Section 2.2 are actually comparatively minor components of the essential oil of *A. annua*, that have been identified solely on this basis. Although it is also possible to analyse more highly oxygenated sesquiterpenes, such as artemisinin and its biosynthetic precursors by GC-MS [55] (Section 2.6), these less volatile components are generally more easily isolated by liquid chromatography (LC) [15]. LC is definitely the technique of choice for the more non-volatile compounds of the extract of *A. annua*, which include flavonoids (Section 2.4), and many triterpenoids and sterols (Section 2.7). In the preparative mode, the LC techniques [56] of column chromatography and high performance liquid chromatography (HPLC) can provide sufficient material to allow for the subsequent structural elucidation of completely novel metabolites, when used in conjunction with techniques such as nuclear magnetic resonance (NMR) spectroscopy and X-ray crystallography. Most of the structures of the more highly oxygenated cadinane and amorphane sesquiterpenoids from *A. annua* which are reported in Section 2.6.3, were determined by this more powerful, but also more laborious approach. The majority of these components are unique to this species and several have turned out to be relevant to the biosynthesis of artemisinin. 

The highly oxygenated nature of many of the terpenoid metabolites from *A. annua* has been stressed throughout this review, because of its perceived relevance to the biosynthesis of artemisinin. In particular, the reader’s attention has been drawn to the unusually wide diversity of terpenoid allylic hydroperoxides and endoperoxides which have been recorded from this species. It is suggested that several of these peroxides result from the reaction of molecular oxygen with the tri-substituted double bond of an appropriate (and frequently abundant) mono- sesqui- or diterpene precursor. Thus, allylic hydroperoxides are found for monoterpenes (**264** and **265**; Section 2.5.1), sesquiterpenes (**414** and **481**; Section 2.6.3) and diterpenes (**553**; Section 2.7.1), all of which might be derived from abundant hydrocarbon precursors; in addition to one monoterpene endoperoxide (**329**; Section 2.5.3) and three sesquiterpene endoperoxides (**465**, **495** and **497**; Section 2.6.3). These reactive hydroperoxides might then be responsible for the formation of a large number of the highly-oxygenated terpenes reported from *A. annua*.

Finally, it has recently been proposed that the the yin-yang nature of Chinese herbal medicine might equate to antioxidation-oxidation in modern parlance [57]. If this is true, then the wide-ranging antioxidant properties associated with the various terpenoidal components from *A. annua* (*i.e.* their propensity to undergo spontaneous autoxidation) would be entirely consistent with its classification as a cooling herb in the TCM system.

### 2.1. Aliphatic Hydrocarbons, Alcohols, Aldehydes and Acids

All possible saturated unbranched hydrocarbons between C_16_ [hexadecane; (**5**)] and C_26_ [hexacosane; (**14**)] have now been reported from GC-MS studies of *A. annua* (Table 1).

**Table 1:** Saturated Unbranched Hydrocarbons.

Structure	Name	CAS Number	References
CH_3_(CH_2_)_3_CH_3_	Pentane (**1**)	[109-66-0]	[58]
CH_3_(CH_2_)_4_CH_3_	Hexane (**2**)	[110-54-3]	[22]
CH_3_(CH_2_)_10_CH_3_	Dodecane (**3**)	[112-40-3]	[24]
CH_3_(CH_2_)_11_CH_3_	Tridecane (**4**)	[629-50-5]	[24]
CH_3_(CH_2_)_14_CH_3_	Hexadecane (**5**)	[544-76-3]	[24]
CH_3_(CH_2_)_15_CH_3_	Heptadecane (**6**)	[629-78-7]	[27]
CH_3_(CH_2_)_16_CH_3_	Octadecane (**7**)	[593-45-3]	[32,43]
CH_3_(CH_2_)_17_CH_3_	Nonadecane (**8**)	[629-92-5]	[27,32,43]
CH_3_(CH_2_)_18_CH_3_	Eicosane (**9**)	[112-95-8]	[32,43]
CH_3_(CH_2_)_19_CH_3_	Heneicosane (**10**)	[629-94-7]	[32,34]
CH_3_(CH_2_)_21_CH_3_	Tricosane (**11**)	[638-67-5]	[32]
CH_3_(CH_2_)_22_CH_3_	Tetracosane (**12**)	[646-31-1]	[32]
CH_3_(CH_2_)_23_CH_3_	Pentacosane (**13**)	[629-99-2]	[32]
CH_3_(CH_2_)_24_CH_3_	Hexacosane (**14**)	[630-01-3]	[32]
CH_3_(CH_2_)_27_CH_3_	Nonocosane (**15**)	[630-03-5]	[58]
CH_3_(CH_2_)_32_CH_3_	Tetratriacontane (**16**)	[14167-59-0]	[59]

**Table 2:** Saturated Unbranched Alcohols, Aldehydes and Ketones.

Structure	Name	Alternative Name(s)	CAS Number	References
*Alcohols*				
CH_3_(CH_2_)_3_CH_2_OH	1-Pentanol (**17**)	Pentyl alcohol	[71-41-0]	[24]
CH_3_(CH_2_)_4_CH_2_OH	*n*-Hexanol (**18**)		[111-27-3]	[22, 32, 43]
CH_3_(CH_2_)_4_CH_2_O-(C=O)CH_2_CH(CH_3_)_2_	*n*-Hexyl isovalerate (**19**)	3-Methylbutyric acid hexyl ester	[10032-13-0]	[32, 43]
CH_3_(CH_2_)_4_CH_2_O-(C=O)C(CH_3_)=CHCH_3_	*n*-Hexyl tiglate (**20**)	(2*E*)- 2-Butenoic acid, 2-methyl-, hexyl ester	[16930-96-4]	[32, 43]
CH_3_(CH_2_)_6_CH_2_OH	1-Octanol (**21**)	Caprylic alcohol	[111-87-5]	[24]
CH_3_(CH_2_)_7_CH_2_OH	*n*-Nonyl alcohol (**22**)	1-Nonanol	[143-08-8]	[24]
CH_3_(CH_2_)_26_CH_2_OH	Octacosanol (**23**)		[557-61-9]	[59, 60, 61]
CH_3_(CH_2_)_27_CH_2_OH	Nonacosanol (**24**)		[6624-76-6]	[59, 62]
*Aldehydes and Ketones*				
CH_3_COCH_3_	Acetone (**25**)	2-Propanone	[67-64-1]	[24]
CH_3_(CH_2_)_2_CHO	Butanal (**26**)	Butyraldehyde		[50]
CH_3_(CH_2_)_4_CHO	Hexanal (**27**)	Caproic aldehyde	[66-25-1]	[23]
CH_3_CO(CH_2_)_4_CH_3_	2-Heptanone (**28**)	Methyl pentyl ketone	[110-43-0]	[45]
CH_3_(CH_2_)_6_CHO	Octanal (**29**)	Capric aldehyde	[124-13-0]	[32]
CH_3_(CH_2_)_11_CHO	Tridecanal (**30**)	Tridecyl aldehyde	[10486-19-8]	[24]

The saturated fatty acids listed in Table 3 are likely to be the biogenetic parents of the saturated hydrocarbons, alcohols, aldehydes and ketones which appear in Table 1 and Table 2. Saturated fatty acids have been obtained from *A. annua* as a continuous series between C_12_ [dodecanoic acid; (**36**)] and C_20_ [eicosanoic acid; (**48**)]. Hexadecanoic acid (**41**; C_16_) and octadecanoic acid (**45**; C_18_) are the most dominant saturated lipids from *A. annua* (Table 3), while oleic acid (**80**) (C_18_) is the most abundant unsaturated fatty acid (Table 5).

**Table 3:** Saturated Unbranched Carboxylic Acids and Esters.

Structure	Name	Alternative Name(s)	CAS Number	References
CH_3_CH_2_OCHO	Ethyl formate (**31**)		[109-94-4]	[50]
CH_3_CH_2_CO_2_CH_2_CH_3_	Propanoic acid, ethyl ester (**32**)	Ethyl propionate	[105-37-3]	[50]
CH_3_(CH_2_)_3_CO_2_H	Pentanoic acid (**33**)	Valeric acid	[109-52-4]	[24]
CH_3_(CH_2_)_3_CO_2_C(CH_3_)_3_	Pentanoic acid, *tert*-butyl ester (**34**)	Pentanoic acid, 1,1-dimethylethyl ester	[23361-78-6]	[50]
CH_3_(CH_2_)_8_CO_2_H	Decanoic acid (**35**)	Capric acid	[334-48-5]	[63, 64]
CH_3_(CH_2_)_10_CO_2_H	Dodecanoic acid (**36**)	Lauric acid	[143-07-7]	[63, 64]
CH_3_(CH_2_)_10_CO_2_CH_2_CH_3_	Dodecanoic acid, ethyl ester (**37**)	Ethyl laurate	[106-33-2]	[24]
CH_3_(CH_2_)_11_CO_2_H	Tridecanoic acid (**38**)		[638-53-9]	[63]
CH_3_(CH_2_)_12_CO_2_H	Tetradecanoic acid (**39**)	Myristic acid	[544-63-8]	[23, 63, 64]
CH_3_(CH_2_)_13_CO_2_H	Pentadecanoic acid (**40**)		[1002-84-2]	[63]
CH_3_(CH_2_)_14_CO_2_H	Hexadecanoic acid (**41**)	Palmitic acid	[57-10-3]	[20, 23, 24, 27, 32, 43, 63, 64, 65]
CH_3_(CH_2_)_14_CO_2_CH_3_	Hexadecanoic acid, methyl ester (**42**)	Methyl hexadecanoate Methyl palmitate	[112-39-0]	[27]
CH_3_(CH_2_)_14_CO_2_CH_2_CH_3_	Hexadecanoic acid, ethyl ester (**43**)	Ethyl palmitate	[628-97-7]	[24]
CH_3_(CH_2_)_15_CO_2_H	Heptadecanoic acid (**44**)	Margaric acid	[506-12-7]	[63]
CH_3_(CH_2_)_16_CO_2_H	Octadecanoic acid (**45**)	Stearic acid	[57-11-4]	[27, 43, 63, 64]
CH_3_(CH_2_)_16_CO_2_CH_3_	Octadecanoic acid, methyl ester (**46**)	Methyl octadecanoate Methyl stearate	[112-61-8]	[27]
CH_3_(CH_2_)_17_CO_2_H	Nonadecanoic acid (**47**)		[646-30-0]	[20]
CH_3_(CH_2_)_18_CO_2_H	Eicosanoic acid (**48**)	Arachidic acid	[506-30-9]	[64]
CH_3_(CH_2_)_20_CO_2_H	Docosanoic acid (**49**)	Behenic acid	[112-85-6]	[64]
CH_3_(CH_2_)_22_CO_2_H	Tetracosanoic acid (**50**)	Lignoceric acid	[557-59-5]	[64]
CH_3_(CH_2_)_28_CO_2_(CH_2_)_30_CH_3_	Hentriacontanyl triacontanoate (**51**)	Triacontanoic acid hentriacontyl ester	[135729-36-1]	[59, 62]

**Table 4:** Unbranched Alkenic Hydrocarbons and Alcohols.

Structure	Name	Alternative Name(s)	CAS Number	References
*Hydrocarbons*				
CH_2_=CHCH=CHCH_3_	1,3-Pentadiene (**52**)	1-Methyl-1,3-butadiene	[504-60-9]	[50]
CH_3_CH=CHCH=CHCH_3_	*trans,trans*-2,4-Hexadiene (**53**)		[5194-51-4]	[50]
H_2_C=CHCH=CHCH=CHCH_3_	*trans,trans*-1,3,5-Heptatriene (**54**)		[17679-93-5]	[50]
H_2_C=CH(CH_2_)_4_CH=CH_2_	1,7-Octadiene (**55**)		[3710-30-3]	[50]
H_2_C=CH(CH_2_)_2_CH=CH(CH_2_)_2_CH=CH_2_	*trans*-1,5,9-Decatriene (**56**)		[39139-91-8]	[50]
*Alcohols*				
CH_2_=CHCH_2_CH(OH)CH_3_	4-Pentene-2-ol (**57**)	1-Penten-4-ol	[625-31-0]	[25]
CH2=CH(CH_2_)_3_O-(C=O)CH_2_CH_3_	4-Penten-1-ol, propionate (**58**)	4-Pentenyl propionate	[30563-30-5]	[43]
CH_3_(CH_2_)_2_CH=CHCH_2_OH	(*E*)-2-Hexenol (**59**)	2-Hexen-1-ol	[928-95-0]	[45]
CH_3_CH_2_CH=CHCH_2_CH_2_OH	(*E*)-3-Hexen-1-ol (**60**)		[928-97-2]	[19]
CH_3_CH_2_CH=CHCH_2_CH_2_OH	(*Z*)-3-Hexen-1-ol (**61**)	Phyllol	[928-96-1]	[22, 32, 43]
CH_3_CH_2_CH=CHCH_2_CH_2_O-(C=O)CH_3_	(*E*)-3-Hexen-1-ol, acetate (**62**)		[3681-82-1]	[19]
CH_3_CH_2_CH=CHCH_2_CH_2_O-(C=O)CH_2_CH_3_	(*Z*)-3-Hexenyl propanoate (**63**)		[33467-74-2]	[20]
CH_3_CH_2_CH=CHCH_2_CH_2_O-(C=O)CH_2_CH_2_CH_3_	3-Hexenyl butanoate (**64**)		[2142-93-0]	[23]
CH_3_CH_2_CH=CHCH_2_CH_2_O-(C=O)(CH_2_)_4_CH_3_	3-Hexenyl hexanoate (**65**)		[84434-19-5]	[24]
CH_3_CH_2_CH=CHCH_2_CH_2_O-(C=O)CH_2_CH(CH_3_)_2_	(*Z*)-3-Hexenyl isovalerate (**66**)		[35154-45-1]	[32]
CH_3_CH_2_CH=CHCH_2_CH_2_O-(C=O)C(CH_3_)=CHCH_3_	(*Z*)-3-Hexenyl tiglate (**67**)		[67883-79-8]	[43]
H_2_C=CHCH(OH)(CH_2_)_3_CH_3_	1-Hepten-3-ol (**68**)		[4938-52-7]	[24]
H_2_C=CHCH(OH)(CH_2_)_4_CH_3_	1-Octen-3-ol (**69**)		[3391-86-4]	[31,32,43]
H_2_C=CH(CH_2_)_7_CH_2_OH	9-Decen-1-ol (**70**)		[13019-22-2]	[32, 43]

**Table 5:** Unbranched Alkenic Ketones, Aldehydes, Carboxylic acids and Esters.

Structure	Name	Alternative ame(s)	CAS Number	References
*Ketones and aldehydes*				
H_2_C=CHCH_2_CH_2_CHO	4-Pentenal (**71**)		[2100-17-6]	[50]
CH_3_(CH_2_)_2_CH=CHCHO	2-Hexenal (**72**)	Leaf aldehyde	[505-57-7] [6728-26-3]	[24, 31, 43]
CH_3_(CH_2_)_3_CH=CHCHO	2-Heptenal (**73**)		[2463-63-0]	[24]
CH_3_(CH_2_)_5_CH=CHCHO	(*Z*)-2-Nonenal (**74**)		[60784-31-8]	[50]
CH_3_(CH_2_)_3_CH=CHCH=CHCHO	(2*E*,4*E*)- Nonadienal (**75**)		[5910-87-2]	[19]
CH_3_(CH_2_)_4_CH=CHCOCH_3_	3-Nonen-2-one (**76**)		[14309-57-0]	[24]
CH_3_(CH_2_)_6_CH=CHCHO	2-Decenal (**77**)		[3913-71-1]	[24]
CH_3_(CH_2_)_4_CH=CHCH=CHCHO	2,4-Decadienal (**78**)		[2363-88-4]	[24]
H_2_C=CH(CH_2_)_8_CHO	10-Undecenal (**79**)		[112-45-8]	[24]
*Carboxylic acids and esters*				
CH_3_(CH_2_)_7_CH=CH(CH_2_)_7_CO_2_H	Oleic acid (**80**)	(*Z*)-9-Octadecanoic acid	[112-80-1] [27104-13-8]	[28, 63, 64]
CH_3_(CH_2_)_7_CH=CH(CH_2_)_7_CO_2_CH_3_	Methyl 9-octadecenoate (**81**)	Methyl elaidate	[2462-84-2]	[24]
CH_3_(CH_2_)_7_CH=CH(CH_2_)_7_CO_2_CH_2_CH(OH)CH_2_OH	9-Octadecenoic acid, 2,3-dihydroxypropyl ester (**82**)		[251983-54-7]	[24]
CH_3_(CH_2_)_4_CH=CHCH_2_CH=CH(CH_2_)_7_CO_2_H	Linoleic acid (**83**)	(*Z*,*Z*)-9,12-Octadecadienoic acid	[60-33-3] [27213-43-0] [28984-77-2]	[20, 63]
CH_3_CH_2_CH=CHCH_2_CH=CHCH_2_CH=CH(CH_2_)_7_CO_2_H	α-Linolenic acid (**84**)	(*Z*,*Z*,*Z*)-9,12,15-Octadecatrien-oic acid	[463-40-1]	[20, 63]

**Table 6:** Branched Aliphatic Hydrocarbons and Alcohols.

Structure	Name	Alternative Name(s)	CAS Number	References
*Hydrocarbons*				
CH_3_(CH_2_)_8_CH(CH_3_)CH_2_CH_2_CH(CH_3_)_2_	Tetradecane, 2,5-dimethyl- (**85**)		[56292-69-4]	[24]
(CH_3_)_2_CH(CH_2_)_26_CH(CH_3_)_2_	Triacontane, 2,29-dimethyl- (**86**)	2,29-Dimethyltriacontane	[135729-37-2]	[62]
(CH_3_)_2_CHC(CH_3_)_3_	2,2,3-Trimethylbutane (**87**)		[464-06-2]	[50]
CH_3_(CH_2_)_3_CH(CH_3_)(CH_2_)_7_CH_3_	Tridecane, 5-methyl- (**88**)	5-Methyltridecane	[25117-31-1]	[24]
*Alcohols*				
(CH_3_)_2_CHCH_2_CH_2_O-(C=O)CH_3_	3-Methyl-1-butanol, acetate (**89**)	Isoamyl acetate	[123-92-2]	[43]
(CH_3_)_2_CHCH_2_O-(C=O)CH_2_CH_3_	2-methylpropylpropionate (**90**)	Isobutyl propionate	[540-42-1]	[50]
(CH_3_)_2_C(OH)(CH_2_)_2_CH_3_	2-Methyl-2-pentanol (**91**)		[590-36-3]	[25]
(CH_3_)_2_C(OH)(CH_2_)_3_CH_3_	2-Methyl-2-hexanol (**92**)		[625-23-0]	[24]
(CH_3_)_2_CH(CH_2_)_3_CH_2_OH	5-Methyl-1-hexanol (**93**)	1-Hexanol, 5-methyl-	[627-98-5]	[24]
*Aldehydes and Ketones*				
(CH_3_)_2_CHCH_2_CHO	3-Methylbutanal (**94**)	Isovaleraldehyde	[590-86-3]	[24]
CH_3_CH_2_CH(CH_3_)CH_2_CHO	3-Methylpentanal (**95**)		[15877-57-3]	[20]
(CH_3_)_2_CHCH_2_COCH_3_	4-Methyl-2-pentanone (**96**)	Isobutyl methyl ketone	[108-10-1]	[24]
(C_6_H_5_)CH_2_CH(CHO)(CH_2_)_5_CH_3_	2-Benzyloctanal (**97**)	Benzenepropanal, α-hexyl-	[161403-65-2]	[24]
(CH_3_)_2_CH(CH_2_)_5_C=O(CH_2_)_14_CH_2_OH	8-Tricosanone, 23-hydroxy-2-methyl- (**98**)		[135729-35-0]	[59, 62]

**Table 7:** Branched Aliphatic Carboxylic Acids, Esters and Anhydrides.

Structure	Name	Alternative Name(s)	CAS Number	References
CH_3_CH_2_CH(CH_3_)CO_2_H	2-Methylbutanoic acid (**99**)		[868-57-5]	[22]
CH_3_CH_2_CH(CH_3_)CO_2_CH_2_CH_3_	2-Methyl butanoic acid, ethyl ester (**100**)	Ethyl 2-methylbutyrate	[7452-79-1]	[22, 32]
(CH_3_CH_2_CH(CH_3_)CO_2_)O	2-Methylbutanoic acid anhydride (**101**)	2-Methylbutyryl anhydride	[1519-23-9]	[50]
CH_3_CH_2_CH(CH_3_)CO_2_(CH_2_)_4_CH_3_	Amyl 2-methylbutyrate (**102**)	Pentyl 2-methylbutanoate	[68039-26-9]	[43]
CH_3_CH_2_CH(CH_3_)CO_2_CH_2_CH(CH_3_)CH_2_CH_3_	2-Methyl-butanoic acid, 2-methylbutyl ester (**103**)	2-Methylbutyl 2-methylbutyrate	[2445-78-5]	[31]
(CH_3_)_2_CHCH_2_CO_2_CH_2_CH_3_	3-Methylbutanoic acid, ethyl ester (**104**)	Ethyl 3-methylbutanoate Ethyl isovalerate	[108-64-5]	[23]
(CH_3_)_2_CHCH_2_CO_2_(CH_2_)_3_CH_3_	3-Methylbutanoic acid, butyl ester (**105**)	Butyl-3-methylbutanoate	[109-19-3]	[25]
(CH_3_)_2_CHCH_2_CO_2_CH_2_CH_2_C(=CH_2_)CH_3_	3-Methylbutanoic acid, 3-methyl-3-butenyl ester (**106**)	3-Methyl-3-butenyl 3-methylbutyrate	[54410-94-5]	[20]
CH_3_CH_2_CH(CH_2_CH_3_)CO_2_CH_3_	2-Ethylbutanoic acid, methyl ester (**107**)	2-Methyl-ethylbutanoate	[816-11-5]	[25]

**Table 8:** Branched Alkenic Hydrocarbons and Aldehydes.

Structure	Name	Alternative Name(s)	CAS Number	References
*Hydrocarbons*				
H_2_C=CHC(CH_3_)_3_	3,3-Dimethyl-1-butene (**108**)	*tert-*Butylethylene	[558-37-2]	[20]
H_2_C=CHCH(CH_3_)_2_	2,4-Dimethyl-2-pentene (**109**)		[625-65-0]	[24]
CH_3_CH=CHCH(CH_3_)CH_2_CH_3_	*trans*-4-Methyl-2-hexene (**110**)		[3683-22-5]	[20]
*Aldehydes*				
CH_3_(CH_2_)_4_CH=CH(CHO)(CH_2_)_3_CH_3_	2-Butyl-2-octenal (**111**)		[13019-16-4]	[24]
(CH_3_)_2_C=CHCH=CH(C=O)CH_3_	6-Methyl-3,5-heptadien-2-one (**112**)		[16647-04-4]	[66]

*Artemisia annua* contains relatively few polyacetylenes by comparison with other members of the genus *Artemisia* (and also as compared to other members of the Asteraceae family). The only two such compounds to be reported to date are ponticaepoxide (**121**) and annuadiepoxide (**122**), which have been isolated from both leaves [67] and seeds [9] of *A. annua*.

**Table 9:** Acetylenic Hydrocarbons.

Structure	Name	Alternative Name(s)	CAS Number	References
CH_3_C≡CCH_2_OMe	1-Methoxy-2-butyne (**113**)		[2768-41-4]	[50]
H_2_C=CH(CH_2_)_3_C≡CH	1-Hepten-6-yne (**114**)		[65939-59-5]	[50]
CH_3_O(C=O)C≡C(CH_2_)_5_CH_3_	2-Nonynoic cid, methyl ester (**115**)	Methyl 2-nonynoate	[111-80-8]	[50]
CH_3_(CH_2_)_9_C≡CH	1-Dodecyne (**116**)	Decylacetylene	[765-03-7]	[23]
CH_3_C≡C(CH_2_)_8_CH_2_OH	10-Dodecyn-1-ol (**117**)		[69221-99-4]	[23]
(CH_3_)_2_CHC≡CCH=CHCH(CH_3_)_2_	3-Octen-5-yne, 2,7-dimethyl- (**118**)		[91400-77-0]	[50]
CH_3_(CH_2_)_6_CH=CHC≡CH_3_	3-Undecen-1-yne (**119**)		[74744-32-4] [91250-91-8]	[23]
(C_6_H_5_)CH_2_C≡C-C≡C-CH_3_	Capillene (**120**)	2,4-Hexadiynylbenzene	[520-74-1]	[30]
	Ponticaepoxide (**121**)	2-Ethenyl-3-(1-nonen-3,5,7-triynyl)oxirane 2-(1-Nonen-3,5,7-triynyl)-3-vinyloxirane 3,4-Epoxy-1,5-tridecadiene-7,9,11-triyne	[3562-36-5]	[9, 67]
	Annuadiepoxide (**122**)	1,3,5-Tridecatriene-7,9,11-triyne (*E*,*E*), 3,4:5,6-diepoxide 3,4:5,6-Diepoxy-1-tridecene-7,9,11-triyne	[139122-80-8]	[9, 67]

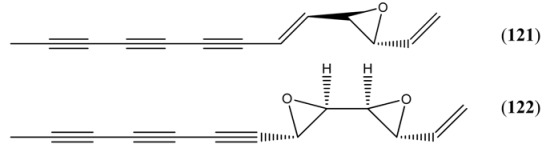

**Table 10:** Cyclic Hydrocarbons.

Name	Alternative Name(s)	CAS Number	References
1,1’-Bicyclopropyl, 2,2’-dimethyl (**123**)		[1975-84-6]	[23]
Bicyclo[2.2.2]octa-2,5-diene, 1,2,3,6-tetramethyl- (**124**)		[62338-43-6]	[20]
3,5-Cycloheptadienone (**125**)		[1121-65-9]	[28]
Cyclooctane, 1,4-dipropyl- (**126**)		[251983-53-6]	[24]
Cyclopropane, (1-methyl-1,2-propadien-1-yl)- (**127**)	3-Cyclopropyl-1,2-butadiene	[51549-86-1]	[50]
Cyclopropene, 3-ethenyl-3-methyl-(**128**)	3-Methyl-3-vinylcyclopropene	[71153-30-5]	
1,1-Dicyclopropylethylene (**129**)	Cyclopropane, 1,1’-ethenylidenebis-	[822-93-5]	[50]
Hexylcyclohexane (**130**)		[4292-75-5]	[20]
Jasmone (**131**)	3-Methyl-2-(2-pentenyl)-2-cyclopenten-1-one	[488-10-8]	[22, 32, 43]
Methyl cyclopentane (**132**)		[96-37-7]	[22]

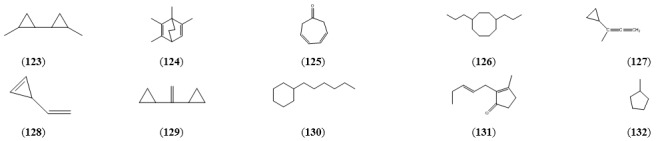

**Table 11:** Furans.

Name	Alternative Name(s)	CAS Number	References
2,5-Dihydro-3-methylfuran (**133**)		[1708-31-2]	[50]
2-Ethylfuran (**134**)		[3208-16-0]	[50]
4-Methyl-2,3-dihydrofuranfuran (**135**)		[34314-83-5]	[34]
3-Methylfuran (**136**)		[930-27-8]	[34]
5-Methyl-2-furancarboxyaldehyde (**137**)	5-Methylfurfural	[620-02-0]	[24]

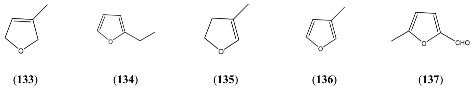

### 2.2. Aromatic Alcohols, Ketones and Acids

The simple aromatic compounds described in Table 12, Table 13 and Table 14 form a relatively small group of natural products from *A. annua*. Metabolites in this section are probably biosynthesised by both the polyketide and shikimate pathways (some simple aromatics which are derived from the terpenoid pathway are discussed separately in Section 2.5.3).

**Table 12:** Simple Phenols and Benzylic Alcohols.

Name	Alternative Name(s)	CAS Number	References
Anisole (**138**)	Phenyl methyl ether	[100-66-3]	[48]
Benzyl isovalerate (**139**)	3-Methylbutanoic acid, benzyl ester	[103-38-8]	[22, 23, 25, 32, 43]
Benzyl 2-methyl butyrate (**140**)	2-Methylbutanoic acid, benzyl ester	[56423-40-6]	[41]
Benzyl phenylacetate (**141**)	Benzeneacetic acid, phenylmethyl ester	[102-16-9]	[50]
Benzyl valerate (**142**)	Benzyl pentanoate	[10361-39-4]	[19, 23]
5-Nonadecylresorcinol-3-*O*-methyl ether (**143**)	Phenol 3-methoxy-5-nonadecyl		[68]

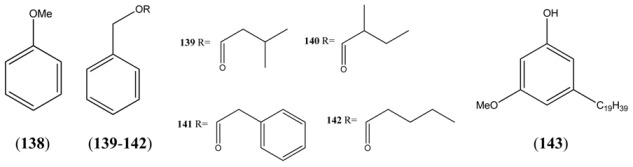

**Table 13:** Simple Aryl Ketones.

Name	Alternative Name(s)	CAS number	References
2’,4’,6’-Trihydroxyacetophenone 2’,4’-dimethyl ether (**144**)	2-Hydroxy-4,6-dimethoxyacetophenone		[68]
2’,4’,6’-Trihydroxyacetophenone 2’-methyl ether (**145**)	2’,4’-dihydroxy-6’-methoxyacetophenone	[3602-54-8]	[68, 69]
2’,4’,6’-Trihydroxyacetophenone 2’-methyl ether 4’-*O*-β-D-glucopyranosde (**146**)	Annphenone	[61775-18-6]	[70]
2’,4’,6’-Trihydroxyacetophenone 4’-methyl ether 2-*O*-β-D-glucopyranoside (**147**)	Domesticoside	[24587-97-1]	[15]

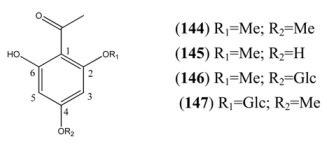

**Table 14:** Simple Benzoic Acids and their Homologues.

Name	Alternative Name(s)	CAS Number	References
Benzoic acid (**148**)		[65-85-0]	[9, 24]
Salicylic acid (**149**)	2-Hydroxybenzoic acid	[69-72-7]	[15]
Methyl salicylate (**150**)	Methyl-2-hydroxybenzoate	[119-36-8]	[27, 32]
2-Hydroxybenzoic acid, 3-methylbutyl ester (**151**)	Isoamyl salicylate	[87-20-7]	[24]
3,4-Dihydroxybenzoic acid (**152**)	Benzoic acid, 3,4-dihydroxy- Protocatechuic acid	[99-50-3]	[71]
Protocatechuic acid 4-glucoside (**153**)	Benzoic acid, 4-(β-D-glucosyloxy)-3-hydroxy-	[7361-59-3]	[71]
Phenylacetic acid (**154**)		[103-82-2]	[24]
Phenylpropanoic acid (**155**)			[9]
Benzenepropanoic acid, 3-cyanophenyl ester (**156**)		[40123-39-5]	[50]

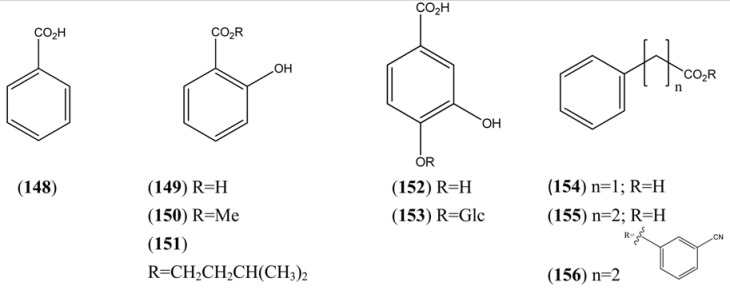

### 2.3. Phenylpropanoids

The phenylpropanoids, all of which contain a C_3_ substituent fused to a benzene ring (C_6_), are produced by the shikimate pathway, which is unique to plants. Most of the simple phenylpropanoids reported in Table 15 were described from GC-MS studies. Compounds **166**-**182** (Table 16), which are esters formed by various combinations of ferulic and cinnamic acid with the four hydroxyl groups of quinic acid, were isolated from a single HPLC-MS study [71]. Some of the coumarins reported in Table 17 have also been obtained from undifferentiated tissue cultures (callus and suspension) of *A. annua* (see Section 4.2). The structures of both of the 2,2-dihydroxychromene natural products **190** and **191**, which appear in Table 17, are questionable on thermodynamic grounds - one might expect both to lose a molecule of water thereby forming a more highly conjugated coumarin. 

**Table 15:** Simple Phenylpropanoids.

Name	Alternative Name(s)	CAS Number	References
*p*-Allylanisole (**157**)	1-Methoxy-4-(2-propenyl)benzene	[140-67-0]	[29, 72, 73]
Anethole (**158**)	1-Methoxy-4-(1-propenyl)benzene	[4180-23-8]	[32, 43, 50]
3-Allyl-6-methoxyphenol (**159**)	4-Allyl-2-hydroxyl-1-methoxybenzene	[501-19-9]	[48]
Eugenol (**160**)	2-Methoxy-4-(2-propenyl)phenol	[97-53-0]	[19, 32, 41, 43, 74]
Methyl eugenol (**161**)	1,2-Dimethoxy-4-(2-propenyl)benzene	[93-15-2]	[43, 48]
Eugenyl isovalerate (**162**)	2-Methoxy-4-(2-propenyl)phenol 3-methylbutanoyl	[61114-24-7]	[34]
2-Methoxy-3-(2-propenyl)phenol (**163**)		[1941-12-4]	[23]

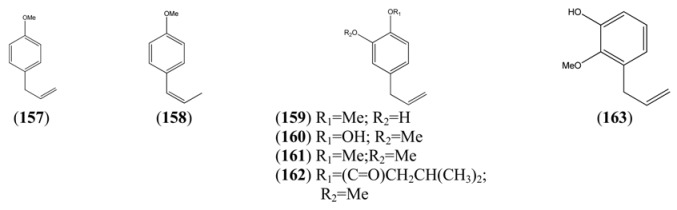

**Table 16:** Cinnamyl, Caffeoyl and Ferruloyl esters.

Name	Alternative Name(s)	CAS Number	Refs
Methyl cinnamate (**164**)	3-phenyl-2-propenoic acid methyl ester	[103-26-4]	[24]
Benzyl cinnamate (**165**)	3-Phenyl-2-propenoic acid benzyl ester	[103-41-3]	[24]
Chlorogenic acid (**166**)	3-(3,4-Dihydroxycinnamoyl)quinic acid	[327-97-9]	[71]
Cyclohexanecarboxylic acid, 1,3,4-trihydroxy-5-[[3-(4-hydroxy-3-methoxyphenyl)-1-oxo-2-propenyl]oxy]-, (**167**)		[53905-80-9]	[71]
Cyclohexanecarboxylic acid, 3-[[3-(3,4-dihydroxyphenyl)-1-oxo-2-propenyl]oxy]-1,4,5-trihydroxy-, (**168**)		[342811-68-1]	[71]
Isochlorogenic acid B (**169**)	3,4-Di-*O*-caffeoylquinic acid	[4534-61-3]	[71]
3-Caffeoyl-4-feruloylquinic acid (**170**)	4-*O*-Feruloyl-5-*O*-caffeoylquinic acid	[125132-81-2]	[71]
3,4-Diferuoylquinic acid (**171**)		[342811-70-5]	[71]
Isochlorogenic acid A (**172**)	3,5-*bis*-(3,4-Dihydroxycinnamoyl)quinic acid)	[2450-53-5]	[71]
3-Caffeoyl-5-feruloylquinic acid (**173**)		[478156-24-0]	[71]
3-Feruloyl-5-caffeoylquinic acid (**174**)		[1039007-73-2]	[71]
3,5-Diferuoylquinic acid (**175**)		[333753-65-4]	[71]
Isochlorogenic acid C (**176**)	4,5-Di-*O*-caffeoylquinic acid	[57378-72-0]	[71]
4-Caffeoyl-5-feruloylquinic acid (**177**)		[478156-25-1]	[71]
4-Feruloyl-5-caffeoylquinic acid (**178**)		[882535-14-0]	[71]
4,5-Diferuoylquinic acid (**179**)		[342811-69-2]	[71]
Cyclohexanecarboxylic acid, 3-[[3-(3,4-dihydroxyphenyl)-1-oxo-2-propenyl]oxy]-4,5-dihydroxy-1-[[3-(4-hydroxy-3-methoxyphenyl)-1-oxo-2-propenyl]oxy]-, (**180**)		[865095-58-5]	[71]
1-Caffeoyl-5-feruoylquinic acid (**181**)		[865095-57-4]	[71]
Cyclohexanecarboxylic acid, 3,4,5-tris[[3-(3,4-dihydroxy phenyl)-1-oxo-2-propenyl]oxy]-1-hydroxy-(**182**)		[437611-66-0]	[71]

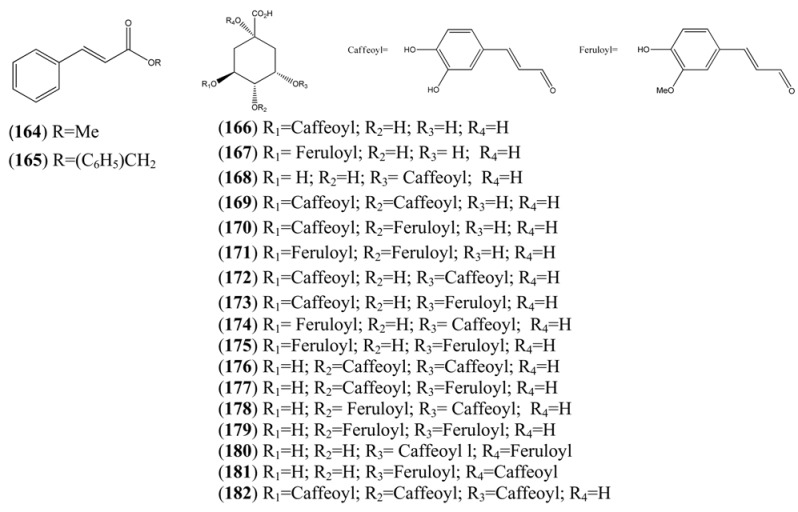

**Table 17:** Coumarins (*2H*-1-Benzopyran-2-one, 9CI).

Name	Alternative Name(s)	CAS Number	References
Coumarin (**183**)	*2H*-1-Benzopyran-2-one	[91-64-5]	[56, 65, 68, 75, 76, 77, 79, 80]
Scopoletin (**184**)	7-Hydroxy-6-methoxycoumarin 7-Hydroxy-6-methoxy-*2H*-1-benzopyran-2-one	[92-61-5]	[77, 79, 80, 79, 80, 81, 82, 83, 84, 85, 86, 87]
Scoparone (**185**)	6,7-Dimethoxycoumarin 6,7-Dimethoxy-*2H*-1-benzopyran-2-one	[120-08-1]	[69, 88, 89]
Scopolin (**186**)	Scopoletin-*O*-β-D-glucopyranoside 7-Hydroxy-6-methoxycoumarin-*O*-β-D-glucopyranoside	[531-44-2]	[15, 71, 79, 89, 90]
Isofraxidin (**187**)	6,8-Dimethoxy-7-hydroxy coumarin	[486-21-5]	[79, 86, 87, 89]
Tomentin (**188**)	5,6,7-Trihydroxy-*2H*-1-benzopyran-2-one 6,7-dimethyl ether	[28449-62-9]	[89]
6,7-Dimethoxydihydrocoumarin (**189**)	2H-1-Benzopyran-2-one,3,4 3,4-Dihydro-6,7-dimethoxy-coumarin	[56680-28-5]	[88]
2,2,6-Trihydroxychromene (**190**)	*2H*-1-Benzopyran-2,2,6-triol	[161585-88-2]	[89]
2,2-Dihydroxy-6-methoxy-*2H*-1-benzopyran (**191**)	6-Methoxy-*2H*-1-benzopyran-2,2-diol *2H*-1-Benzopyran-2,2,6-triol 6-methyl ether	[161585-87-1]	[89]

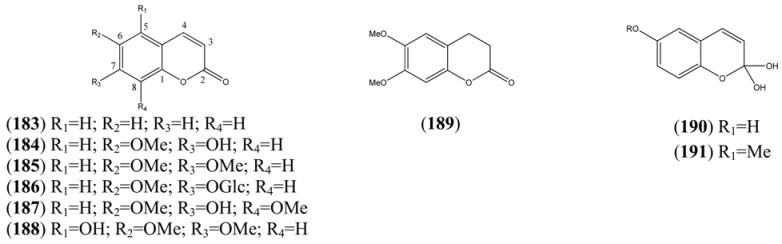

### 2.4. Flavonoids

*A. annua* L. is a rich source of flavonoids, as are many other members of the Asteraceae family. It has been suggested that some of the more abundant methoxylated flavonoids from *A. annua* may potentiate the antimalarial activity of artemisinin in crude extracts of this plant. These flavones include: casticin (**227**), artemetin (**228**) [91], chrysosplenol D (**225**) and chrysoplenetin (**226**) [92] (interstingly, the latter two flavonoids are also reported to potentiate the activity of berberine and norfloxacin against a resistant strain of *Staphylococcus aureus*). Perhaps for this reason, phytochemical studies have sometimes sought to determine the distribution of bioactive flavonoids (such as chrysoplenetin (**226**), casticin (**227**), eupatin (**232**) and artemetin (**228**)) in conjunction with that of artemisinin [93].

The flavonoids are produced from the cyclization of a C_15_ chalcone precursor, which is in turn derived from malonyl coenzyme A and *p*-coumaryl coenzyme A (see Section 2.3). A biogenetic classification has been adopted in this section. Thus, this large group of flavonoids has been further sub-divided into flavonones (Table 18), which are produced directly by cyclization of this precursor; and flavonols (3-hydroxy-2-phenyl-*4H*-1-benzopyran-4-ones), which have undergone subsequent oxygenation at the 3-position (Table 19, Table 20, Table 21 and Table 22). The flavonols have been further grouped into tetrahydroxyflavonols in Table 19 [many are related to kaempferol (**203**)]; pentahydroxyflavonols in Table 20 [many are based on quercetin (**207**)]; hexahydroxyflavonols, based on quercetagetin (**221**) in Table 21; and miscellaneous hexahydroxyflavonols (Table 22). 

**Table 18:** Flavones (2-Phenyl-*4H*-1-benzopyran-4-one).

Name	Alternative Name(s)	CAS Number	References
Apigenin (**192**)	4’,5,7-Trihydroxyflavone 5,7-Dihydroxy-2-(4-hydroxyphenyl)-*4H*-1-benzopyran-4-one	[520-36-5]	[71, 89]
*Luteolin tetrahydroxyflavones*			
Luteolin (**193**)	3’,4’,5,7-Tetrahydroxyflavone 2-(3,4-Dihydroxyphenyl)-5,7,-dihydroxy-*4H*-1-benzopyran-4-one	[491-70-3]	[84, 89]
Luteolin-7-methyl ether (**194**)	3’,4’,5-Trihydroxy-7-methoxyflavone 2-(3,4-Dihydroxyphenyl)-5-hydroxy-7-methoxy-*4H*-1-benzopyran-4-one	[20243-59-8]	[89]
Glucoluteolin (**195**)	3,4’,5,7-Tetrahydroxyflavone-7-*O*-β-D-glucopyranoside Luteolin 7-glucoside	[5373-11-5]	[84, 89]
Chrysoeriol (**196**)	4’,5,7-Trihydroxy-3’-methoxyflavone 5,7-Dihydroxy-2-(4-hydroxy-3-methoxyphenyl)-*4H*-1-benzopyran-4-one	[491-71-4]	[76, 94]
*Other tetrahydroxyflavones*			
Cirsimaritin (**197**)	4’,5-Dihydroxy-6,7-dimethoxyflavone 5-Hydroxy-2-(4-hydroxyphenyl)-6,7-dimethoxy-*4H*-1-benzopyran-4-one	[6601-62-3]	[76, 94]
*Pentahydroxyflavones*			
Cirsiliol (**198**)	3’,4’,5,6,7-Pentahydroxyflavone 6,7-dimethyl ether 3’,4’,5-Trihydroxy-6,7-dimethoxyflavone	[34334-69-5]	[76, 94]
Eupatorin (**199**)	6-Methoxy luteolin 7,4’-dimethyl ether 3’,5-Dihydroxy-4’,6,7-trimethoxyflavone 5-Hydroxy-2-(3-hydroxy-4-methoxyphenyl)-6,7-dimethoxy-*4H*-1-benzopyran-4-one	[855-96-9]	[76, 94, 95]
5-Hydroxy-3’,4’,6,7-tetramethoxyflavone (**200**)	3’,4’,5,6,7-Pentahydroxyflavone 3’,4’,6,7-tetra methyl ether	[21763-80-4]	[96]
*4H*-1-Benzopyran-4-one, 2-(2,4-dihydroxyphenyl)-5-hydroxy-6,7-dimethoxy- (**201**)		[101909-51-7]	[76]
*Hexahydroxyflavones*			
2,4’,5’-Trihydroxy-5’6,7-trimethoxyflavone (**202**)			[94]

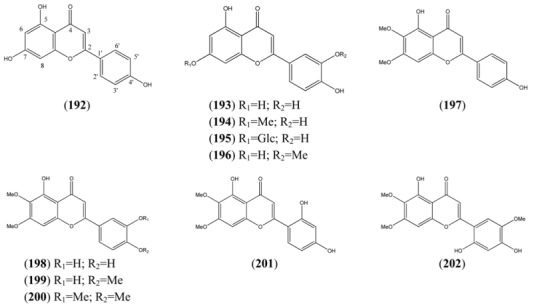

**Table 19:** Tetrahydroxy Flavonols.

Name	Alternative Name(s)	CAS Number	References
*Kaempferols*			
Kaempferol (**203**)	3,4’,5,7-Tetrahydroxyflavone 3,5,7-Trihydroxy-2-(4-hydroxyphenyl)-*4H*-1-benzopyran-4-one 4’,5,7-Trihydroxyflavonol	[520-18-3]	[84, 89]
Kaempferol-3-*O*-glucoside (**204**)	Astragalin 3-*O*-β-D-Glucopyranosyloxy-4’,5,7-trihydroxyflavone	[480-10-4]	[84, 89]
Rhamnocitrin (**205**)	3,4’,5-Trihydroxy-7-methoxyflavone 3,5-Dihydroxy-2-(4-hydroxyphenyl)-7-methoxy-*4H*-1-benzopyran-4-one 4’,5-Dihydroxy-7-methoxyflavonol	[569-92-6]	[76, 94]
*Other Tetrahydroxyflavonols*			
*4H*-1-Benzopyran-4-one, 3-hydroxy-6,7-dimethoxy-2-(4-methoxyphenyl)- (**206**)		[77184-81-7]	[71]

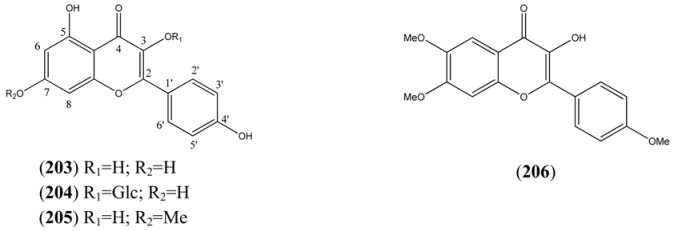

**Table 20:** Pentahydroxy Flavonols.

Name	Alternative Name(s)	CAS Number	Refs
*Quercetin*			
Quercetin (**207**)	3,3’,4’,5,7-Pentahydroxyflavone 2-(3,4-Dihydroxyphenyl)-3,5,7-trihydroxy-*4H*-1-benzopyran-4-one 3’,4’,5,7-Tetrahydroxyflavonol	[117-39-5]	[84]
Quercetin 3-methyl ether (**208**)	3’,4’,5,7-Tetrahydroxy-3-methoxyflavone 2-(3,4-Dihydroxyphenyl)-5,7-dihydroxy-3-methoxy-*4H*-1-benzopyran-4-one	[1486-70-0]	[76, 94]
Isoquercitrin (**209**)	Quercetin-3-glucofuranoside 2-(3,4-Dihydroxyphenyl)-3-(β-D-glucopyranosyloxy)-5,7-dihydroxy-*4H*-1-benzopyran-4-one 3-Glucopyranosyloxy-3’,4’,5,7-tetrahydroxyflavoneQuercetin-3-glucopyranoside	[21637-25-2] [482-35-9]	[71, 89]
Quercetin 3-rutinoside (**210**)		[153-18-4]	[84]
Isorhamnetin 3-glucoside (**211**)	3-Glucopyranosyloxy-4’,5,7-trihydroxy-3’-methoxyflavone	[5041-82-7]	[71]
Rhamnetin (**212**)	3,3’,4’,5-Tetrahydroxy-7-methoxyflavone 2-(3,4-Dihydroxyphenyl)-3,5-dihydroxy-7-methoxy-*4H*-1-benzopyran-4-one 3’,4’5-Trihydroxy-7-methoxyflavonol	[90-19-7]	[76, 94]
Quercimeritrin (**213**)	7-*O*-β-D-glucopyranosyloxy-3,3’,4’,5-tetrahydroxyflavone Quercetin 7-glucoside	[491-50-9]	[89]
Isorhamnetin (**214**)	3,4’,5,7-Tetrahydroxy-3’-methoxyflavone 3,5,7-Trihydroxy-2-(4-hydroxy-3-methoxyphenyl)-*4H*-1-benzopyran-4-one 4’,5,7-Trihydroxy-3’-methoxyflavonol Quercetin 3’-methyl ether	[480-19-3]	[89]
Quercetin 3’-glucoside (**215**)	3,3,’4’,5,7-Pentahydroxyflavone 3’-*O*-β-D-glucopyranoside	[19254-30-9]	[84, 89]
Tamarixetin (**216**)	3,3’,5,7-Tetrahydroxy-4’-methoxyflavone 3,5,7-Trihydroxy-2-(3-hydroxy-4-methoxyphenyl)-*4H*-1-benzopyran-4-one 3’,5,7-Trihydroxy-4’-methoxyflavonol	[603-61-2]	[76, 94]
*Other pentahydroxyflavonols*			
Eupalitin (**217**)	3,4’,5-Trihydroxy-6,7-dimethoxyflavone 3,5-Dihydroxy-2-(4-hydroxyphenyl)-6,7-dimethoxy-*4H*-1-benzopyran-4-one 4’,5-Dihydroxy-6,7-dimethoxyflavonol	[29536-41-2]	[96]
Penduletin (**218**)	3,4’,5,6,7-Pentahydroxyflavone 3,6,7-trimethyl ether 4’,5-Dihydroxy-3,6,7-trimethoxyflavone	[569-80-2]	[76, 94, 97]
3,4’,5,6,7-Pentahydroxyflavone 3,4’,6,7-tetramethyl ether (**219**)	5-Hydroxy-3,4’,6,7-tetramethoxyflavone	[14787-34-9]	[61, 75, 78, 97]
Mikanin (**220**)	3,4’,5,6,7-Pentahydroxyflavone 4’,6,7-trimethyl ether 3,5-Dihydroxy-4’,6,7-trimethoxyflavone	[4324-53-2]	[71]

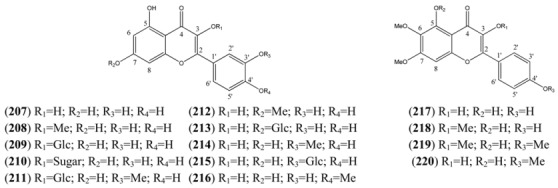

**Table 21:** Quecetagetin (Hexahydroxy) Flavonols.

Name	Alternative Name(s)	CAS Number	References
Quercetagetin 3-methyl ether (**221**)	3’,4’,5,6,7-Pentahydroxy-3-methoxyflavone 2-(3,4-Dihydroxyphenyl)-5,6,7-trihydroxy-3-methoxy-*4H*-1-benzopyran-4-one	[64190-88-1]	[89]
Axillarin (**222**)	Quercetagetin 3,6-dimethyl ether 3’,4’,5,7-Tetrahydroxy-3,6-dimethoxyflavone 2-(3,4-Dihydroxyphenyl)-5,7-dihydroxy-3,6-dimethoxy-*4H*-1-benzopyran-4-one	[5188-73-8]	[76, 84]
Quercetagetin-3,4’-dimethyl ether (**223**)	3’,5,6,7-Tetrahydroxy-3,4’-dimethoxyflavone 3,3’,4’,5,6,7-Hexahydroflavone 3,4’-di-methyl ether	[59171-34-5]	[76]
Bonanzin (**224**)	5,7-Dihydroxy-3,3’,4’,6-tetramethoxyflavone 2-(3,4-Dimethoxyphenyl)-5,7-dihydroxy-3,6-dimethoxy-*4H*-1-benzopran-4-one	[35688-42-7]	[96]
Chrysosplenol D (**225**)	2-(3,4-Dihydroxyphenyl)-5-hydroxy-3,6,7-trimethoxy-*4H*-1-benzopyran-4-one	[14965-20-9]	[15, 76, 84, 92, 94, 95, 98]
Chrysosplenetin (**226**)	Chrysosplenol B 5,4’-Dihydroxy-3,6,7,3’-tetramethoxyflavone 5-Hydroxy-2-(4-hydroxy-3-methoxyphenyl)-3,6,7-trimethoxy-*4H*-1-benzopyran-4-one	[603-56-5]	[15, 76, 80, 84, 91, 92, 95, 96, 99, 100, 101]
Casticin (**227**)	Quercetagetin 6,3,7,4’-tetramethyl ether 3’,5-Dihydroxy-3,4’,6,7-tetramethoxyflavone 5-Hydroxy-2-(3-hydroxy-4-methoxyphenyl)-3,6,7-trimethoxy-*4H*-benzopyran-4-one	[479-91-4]	[76, 84, 91, 94, 95, 98, 99, 100, 103]
Artemetin (**228**)	5-Hydroxy-3,6,7,3’,4’-Pentamethoxyflavone 2-(3,4,-Dimethoxyphenyl)-5-hydroxy-3,6,7-trimethoxy-*4H*-1-benzopyran-4-one	[479-90-3]	[15, 75, 77, 78, 80, 94, 95, 96, 98, 100, 105, 106]
Patuletin-3-*O*-glucoside (**229**)	Quercetagetin 6-methyl ether 3-*O*-glucoside 6-Methoxykaempferol -3-*O*-glucoside	[19833-27-3]	[84]
Patuletin (**230**)	3,3’,4’,5,7-Pentahydroxy-6-methoxyflavone 2-(3,4-Dihydroxyphenyl)-3,5,7-trihydroxy-6-methoxy-*4H*-1-benzopyran-4-one 3’,4’,5,7-Tetrahydroxy-6-methoxyflavonol	[519-96-0]	[84]
Cirsilineol (**231**)	3’,4’,5,6,7-Pentahydroxyflavone 3’,6,7-tri methyl ether 4’,5-Dihydroxy-3’,6,7-trimethoxyflavone	[41365-32-6]	[76, 94, 95, 98]
Eupatin (**232**)	3,3’,5-Trihydroxy-4’,6,7-trimethoxyflavone 3,5-Dihydroxy-2-(3-hydroxy-4-methoxyphenyl)-6,7-dimethoxy-*4H*-1-benzopyran-4-one 3’,5-Dihyhdroxy-4’,6,7-trimethoxyflavonol Quercetagetin 4’,6,7-trimethyl ether	[19587-65-6]	[61, 77, 98]
Quercetagetin-6,7,3’,4’-tetramethylether (**233**)	3,5-Dihydroxy-3’,4’,6,7-tetramethoxyflavone 3,3’,4’,5,6,7-Hexahydroflavone 3’,4’,6,7-tetra methyl ether	[57296-14-7]	[61, 71, 77, 105, 107]
Quercetagetin 4’-methyl ether (**234**)	3,3’,4’,5,6,7-Hexahydroxyflavone 4’-methyl ether 3,3’,5,6,7-Pentahydroxy-4’-methoxyflavone 3’,5,6,7-Tetrahydroxy-4’-methoxyflavonol	[161585-86-0]	[89]

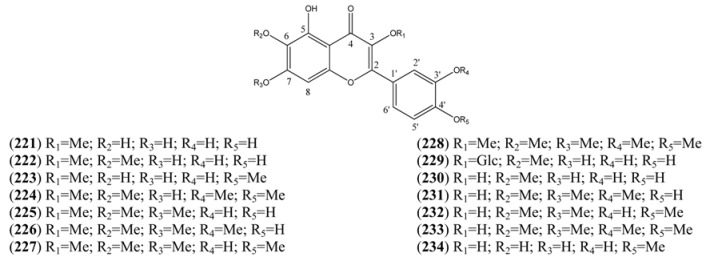

**Table 22:** Other Hexahydroxy Flavonols.

Name	Alternative Name(s)	CAS Number	References
*4H*-1-Benzopyran-4-one 5-hydroxy-2-(2-hydroxy-3,4-di-methoxyphenyl)-3,7-dimethoxy (**235**)		[1186306-45-5]	[15]
*4H*-1-Benzopyran-4-one, 2-(3,5-dihydroxy-4-methoxy-phenyl)-3-(β-D-glucopyranosyloxy)-5,7-dihydroxy- (**236**)		[230283-37-1]	[71]
Mearnsetin (**237**)	3,3’,5,5’,7-Pentahydroxy-4’-methoxy-flavone 2-(3,5-Dihydroxyphenyl-4-methoxy-phenol)-3,5,7-trihydroxy-*4H*-1-benzopyran-4-one 3’,5’,5’,7-Tetrahydroxy-4’-methoxy-flavonol	[16805-10-0]	[71]
Chrysosplenol E (**238**)	2’,3,4’,5,5’,7-Hexahydroxyflavone 3,4’,5’,7-tetramethyl ether 2’,5-Dihydroxy-3,4’,5’,7-tetramethoxy-flavone	[23289-81-8]	[80]
5,3’-Dihydroxy, 3,6,7,5’-tetramethoxyflavone (**239**)			[99]
3’,5,7,8-Tetrahydroxy-3,4’-dimethoxyflavone (**240**)	3,3’,4’,5,7,8-Hexahydroxyflavone 3,4’-di-ethyl ether	[123563-74-6]	[76, 94]

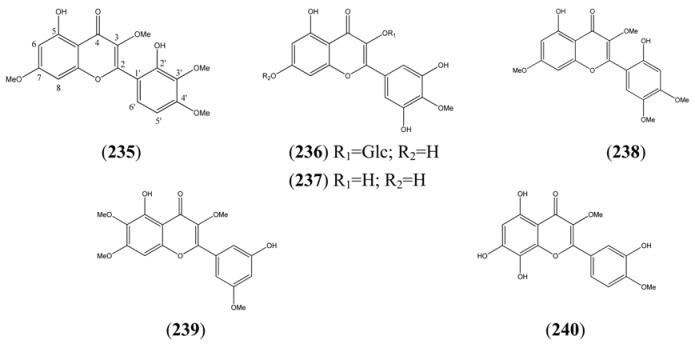

The miscellaneous polycyclic aromatic natural product acenaphthylene, 1,2,4,5-tetrahydro- ([54271-92-0]) (**241**) is also reported from *A. annua* [34].

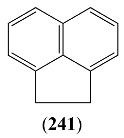



### 2.5. Monoterpenoids

**Figure 2:**
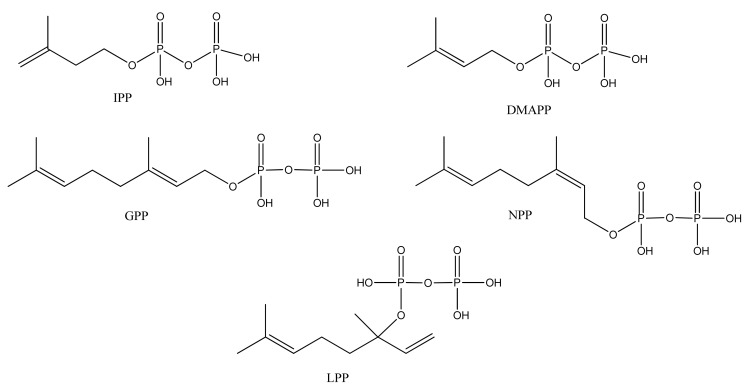
Structures of isopentenyl pyrophosphate (IPP), dimethylallyl pyrophosphate (DMAPP), geranyl pyrophosphate (GPP), neryl pyrophosphate (NPP) and linaloyl pyrophosphate (LPP) which are all possible precursors to monoterpenes from *A.annua*.

Monoterpenoids generally contain ten carbons (C_10_) and are the principal components of the essential oil of *A. annua* obtained by steam distillation (or other techniques that are selective for more volatile natural products). It is widely believed that monoterpenes are located in the glandular trichomes – small structures, which are loosely attached to the surfaces of the leaves and flowers [108]. The regular acyclic monoterpenes which are described in Section 2.5.1 consist of an eight-carbon chain, which is often functionalized at the 1-position, with methyl substituents at the 3- and 7-positions (Table 23). They are formed by ionization of the pyrophosphate group in one of the C_10_ precursors: geranyl pyrophosphate (GPP), neryl pyrophosphate (NPP) or linalyl pyrophosphate (LPP) (Figure 2) [these monoterpene precursors are, in turn, formed from the “head-to-tail” condensation of a “starter” molecule of dimetylallypyrophosphate (DMAPP) with a “chain extender” of isopentenyl pyrophosphate (IPP) (both C_5_)]. *A. annua* is also a rich source of irregular acyclic monoterpenoids (Section 2.5.2), which are derived from the “head-to-middle” condensation of two C_5_ precursors.

The “regular” acyclic monoterpenoid precursors can then undergo further intramolecular reactions to yield monocyclic monoterpenes (Section 2.5.3). The largest group of such monocyclic monoterpenes in *A. annua* is the *p*-menthane series (Table 25), which incorporate a single six-membered ring. Further cyclization produces bicyclic monoterpenes (Section 2.5.4), which may contain an additional five-membered ring (e.g., camphanes in Table 26); or a four-membered ring (pinanes, Table 27); or a three-membered ring (thujanes, Table 28). Studies with ^14^C-labeled LPP have shown that in *A. annua* this precursor is converted to cyclised monoterpenes such as 1,8-cineole (**326**) and α-pinene (**347**) with greater efficiency than the alternative precusors, NPP and GPP [109].

#### 2.5.1. Regular Acyclic Monoterpenes

A cDNA for (3*R*)-linalool synthase, which converts GPP to (3*R*)-linalool (**250**) by ionization of the pyrophosphate group, has been described recently from *A. annua* [110]. Most of the regular acyclic monoterpenes reported in Table 23 can be derived by further functional group modifications (dehydration, reduction or oxidation) of linalool (**250**) or its isomers, geraniol (**244**) and nerol (**248**).

**Table 23:** Regular Acyclic Monoterpenoids (2,6-Dimethyloctane, 9CI, 8CI).

Name	Alternative Name(s)	CAS number	References
Citronellol (**242**)	3,7-Dimethyl-6-octen-1-ol	[106-22-9]	[24]
Citronellal (**243**)	3,7-Dimethyl-6-octenal	[106-23-0]	[28]
Geraniol (**244**)	(*E*)-3,7-Dimethyl-2,6-octadien-1-ol	[106-24-1]	[22]
Geranyl acetate (**245**)	(*E*)-3,7-Dimethyl-2,6-octadien-1-ol acetate	[105-87-3]	[23, 51]
3,7-Dimethyl-2,6-octadienyl, isobutyric acid, ester (**246**)	(*E*)-Isobutyric acid, 3,7-dimethyl-2,6-octadienyl ester	[1188-06-3]	[50]
2,6-Octadien-1-ol, 2,6-dimethyl-8-[(tetrahydro-*2H*-pyran-2-yl)oxy]- (**247**)		[80444-67-3]	[23]
Nerol (**248**)	(*Z*)-3,7-Dimethyl-2,6-octadien-1-ol	[106-25-2]	[23, 58]
Neryl acetate (**249**)	(*Z*)-3,7-Dimethyl-2,6-octadien-1-ol acetate	[141-12-8]	[45]
Linalool (**250**)	3,7-Dimethyl-1,6-octadien-3-ol	[78-70-6]	[19, 28, 31, 32, 45, 51, 74, 111]
Linalyl acetate (**251**)	3,7-Dimethyl-1,6-octadien-3-ol acetate	[115-95-7]	[32, 43]
3,7-Octadien-2-ol, 2,6-dimethyl- (**252**)		[62911-76-6]	[23]
Myrcenol (**253**)	2-Methyl-6-methylene-7-octen-2-ol	[543-39-5]	[30]
Myrcene (**254**)	7-Methyl-3-methylene-1,6-octadiene	[123-35-3]	[19, 23, 29, 32, 33, 34, 43, 56, 50, 51, 73, 112, 113]
Ipsdienol (**255**)	2-Methyl-6-methylene-2,7-octadien-4-ol	[35628-00-3]	[28]
*allo*-Ocimene (**256**)	2,6-Dimethyl-2,4,6-octatriene	[673-84-7]	[22]
*trans*-α-Ocimene (**257**)	3,7-Dimethyl-1,3,7-Octatriene	[27400-72-2] [3779-61-1]	[22, 32, 111]
(*E*)- 3,7-Dimethyl-1,3,6-octatriene (**258**)		[3779-61-1]	[43]
(*Z*)-3,7-Dimethyl-1,3,6-octatriene (**259**)		[3338-55-4]	[32, 43]
2,6-Dimethyl-3,5,7-octatrien-2-ol (**260**)		[103272-78-2]	[23]
3,7-Dimethyl-1,5,7-octatrien-3-ol (**261**)		[29957-43-5]	[48]
2,6-Dimethyl-1,5,7-octatrien-3-ol (**262**)		[29414-56-0]	[23]
3,7-Octadien-2-ol, 2-methyl-6-methylene (**263**)		[22459-09-2]	[31]
β-myrcene hydroperoxide (**264**)	2-Methyl-6-methylene-3,7-octadiene-2-ol (*E*), 2-hydroperoxide		[9, 114]
α- myrcene hydroperoxide (**265**)	2-Methyl-6-methylene-1,7-octadiene, 3-hydro-peroxide		[9, 114]
1,6-Octadien-4-one, 7-methyl-3-methylene- (**266**)		[1079223-79-2]	[43]
1,7-Octadien-3-one, 2-methyl-6-methylene- (**267**)	2-Methyl-6-methylene-1,7-octadien-3-one	[41702-60-7]	[20]
*cis-*Epoxyocimene (**268**)	3,7-Dimethyl-1,3,6-Octatriene 6*R*,7-epoxide	[255832-06-5]	
2,6-Dimethyl-1,3,5,7-octatetraene (**269**)		[90973-78-7]	[50]
Perillene (**270**)	3-(4-Methyl-3-pentenyl)furan	[539-52-6]	[23, 43]
1,10-Oxy-α-myrcene hydroxide (**271**)			[9]
1,10-Oxy-β-myrcene hydroxide (**272**)			[9]

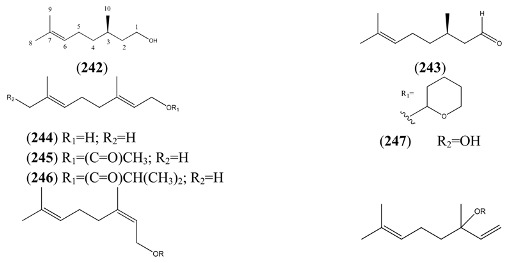
 
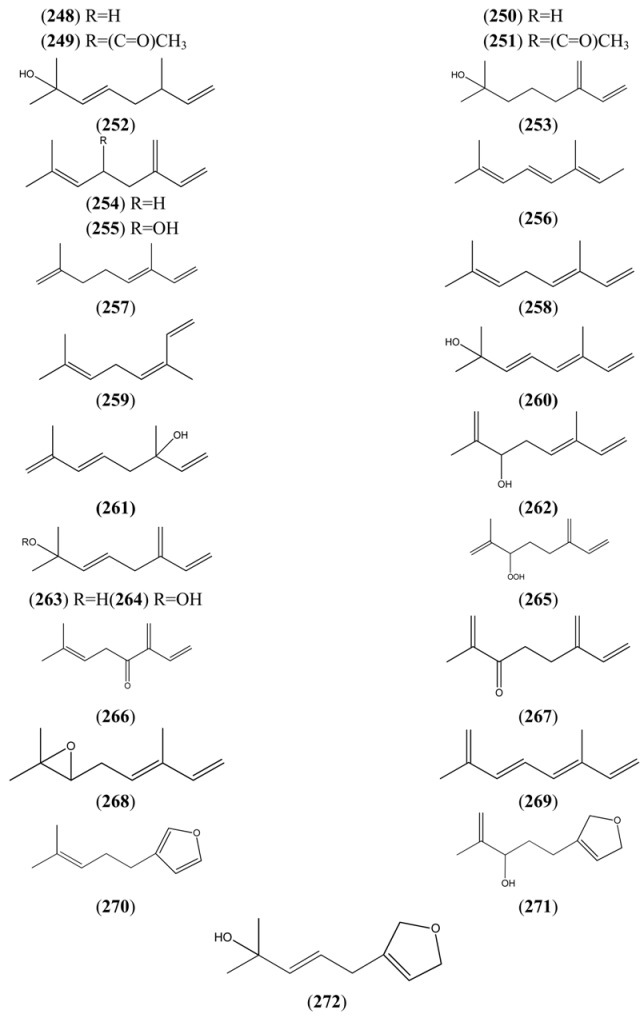

**Scheme 1:**
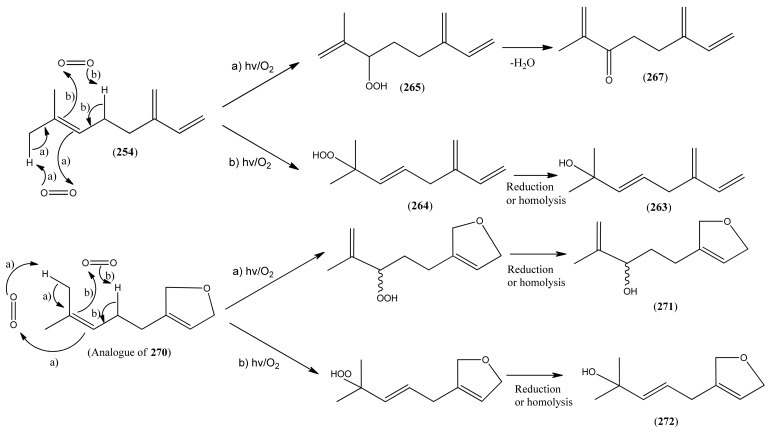
Postulated biosynthesis of allylic hydroperoxides: α-myrcene hydroperoxide (**265**) and β-myrcene hydroperoxide (**264**) via spontaneous autoxidation reactions at the tri-substituted bond of the precursor myrcene (**254**). Further reactions of such allylic hydroperoxides would account for the biogenesis of oxygenated monoterpenes such as (**267**), (**263**), (**271**) and (**272**).

Myrcene (**254**) is the most abundant regular acyclic monoterpene from *A. annua*, and can account for up to 5% of the essential oil [22,33,43]. The secondary and tertiary allylic hydroperoxides, α- and β-myrcene hydroperoxide (**265** and **264**), have been isolated from *A. annua* on more than one occasion [9,114], and are possibly formed by an “ene”-type reaction of molecular oxygen with the tri-substituted double bond in myrcene (**254**), occurring in the presence of light and a photosensitizer (*i.e.* singlet oxygen, ^1^O_2_), as is shown in Scheme 1. Further known reactions of these allylic hydroperoxides might account for the formation of other highly oxygenated myrcene monoterpenoids from *A. annua*, such as compounds **267** and **263** (Scheme 1). This propensity towards spontaneous autoxidation has also been suggested for several other terpenoidal hydroperoxides from *A. annua*, and it is repeatedly stressed in this review because of its perceived relvance to the biosynthesis of artemisinin (Section 3.3). Compounds **271** and **272** are also derivatives of myrcene, in which oxidation at C-1 and C-10 has produced a 1,4-dihydrofuran functionality, rarely found in natural products. It has been proposed that the biogenesis of the secondary and teriary allylic hydroxide functionality in this pair of compounds might also be explained by similar autoxidation reactions of a putatative monoterpene precursor (closely related to perillene (**270**)), which also proceeds *via* a pair of secondary and tertiary allylic hydroperoxide intermediates [9], as is shown in Scheme 1.

#### 2.5.2. Irregular Acyclic Monoterpenes

**Scheme 2:**
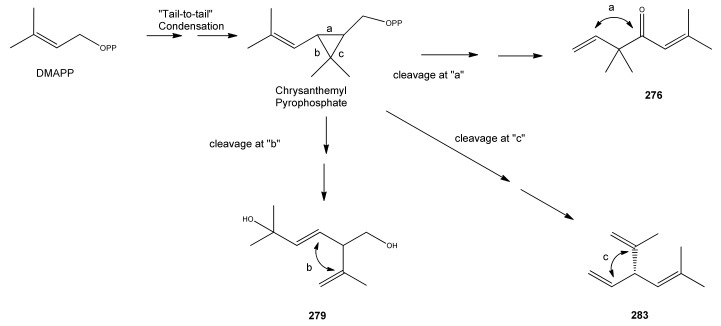
Formation of the irregular artemisyl, lavandulyl and santolinyl skeletons in *A. annua* by “head-to-middle” condensation of a DMAPP (C_5_) precursor and subsequent carbon-carbon cleavage reactions of the resulting intermediate, chryanthemyl pyrophosphate.

Phytochemists were interested in *A. annua* before the discovery of artemisinin because it is a rich source of unusual irregular acyclic monoterpenoids, such as artemisia ketone (**276**) [115,116,117,118,119,120], which is the major constituent of the essential oil in some varieties of *A. annua* [29,37,42,73], and can account for up to 50% of the total [22,25,33,43,45,53]. It is often found in conjunction with smaller amounts of artemisia alcohol (**273**) [22,33,38,121]. Artemisia ketone (**276**) is formed by a non-standard “head-to-middle” condensation of DMAPP [104,122,123,124,125,126], which is thought to proceed as shown in Scheme 2 *via* chrysanthemyl pyrophosphate, an intermediate containing a three-membered ring. The mechanism for the formation of this unusual intermediate [127], is believed to mimic the formation of presqualene, another naturally-occuring cyclopropane, which is involved in the biosynthesis of the triterpene precursor, squalene (see Section 2.7.2) [128]. Two other classes of irregular cyclic monoterpenoids, the lavandulanes **279**)–**281** and the santolinanes **282** and **283**, are also known from *A. annua*. The formation of all three skeletons has been explained in terms of different cleavage reactions occurring at each of the three carbon-carbon bonds in the cyclopropyl ring of the common precursor, chysanthemyl pyrophosphate (Scheme 2) [126].

**Table 24:** Irregular Acyclic Monoterpenoids.

Name	Alternative Name(s)	CAS Number	References
*Artemisanes*			
Artemisia alcohol (**273**)	3,3,6-Trimethyl-1,5-heptadien-4-ol	[29887-38-5] [27644-04-8] [77363-66-7]	[19, 23, 29, 33, 38, 40, 43, 45, 48, 51, 66, 73, 121]
Artemisyl acetate (**274**)	Artemisia alcohol acetate 3,3,6-Trimethyl-1,5-heptadien-4-ol acetate	[3465-88-1] [29887-38-5]	[25, 40, 43, 45, 72, 112, 113]
(*E*)-2-Butenoic acid, 2-methyl-, 2,2-dimethyl-1-(2-methyl-1-propenyl)-3-butenyl ester (**275**)		[62594-30-3]	[30]
Artemisia ketone (**276**)	3,3,6-Trimethyl-1,5-heptadien-4-one	[546-49-6]	[19, 21, 22, 23, 24, 26, 29, 31, 33, 36, 38, 40, 41, 42, 43, 44, 45, 49, 53, 66, 72, 73, 112, 113, 121]
Yomogi alcohol (**277**)	2,5,5-Trimethyl-3,6-heptadien-2-ol	[26127-98-0]	[19, 32, 38, 45]
Artemisiatriene (**278**)	2,5,5-Trimethyl-1,3,6-heptatriene	[29548-02-5]	[23]
*Lavandulanes*			
*trans-*5-Hydroxy-2-isopropenyl-5-methylhex-3-en-1-ol (**279**)	3-Hexene-1,5-diol, 5-methyl-2-(1-methyl-ethenyl)-	[403797-33-1]	[129]
4-Hydroxy-2-isopropenyl-5-methylene-hexan-1-ol (**280**)			[9]
Lavandulyl acetate (**281**)	5-Methyl-2-(1-methylethenyl)-4-hex-en-1-ol	[20777-39-3]	[19]
*Santolinanes*			
Santolina alcohol (**282**)	3-Ethenyl-2,5-dimethyl-4-hexen-2-ol	[35671-15-9]	[19, 32, 43]
Santolinatriene (**283**)	3-Ethenyl-2,5-dimethyl-1,4-hexadiene	[70005-95-7] [2153-66-4]	[23, 25, 43, 45, 50]

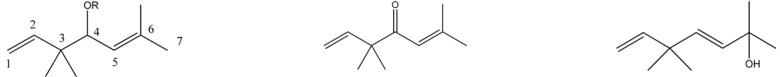
 
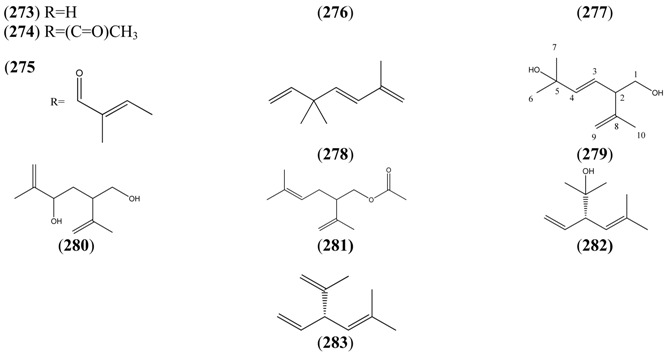

**Scheme 3:**
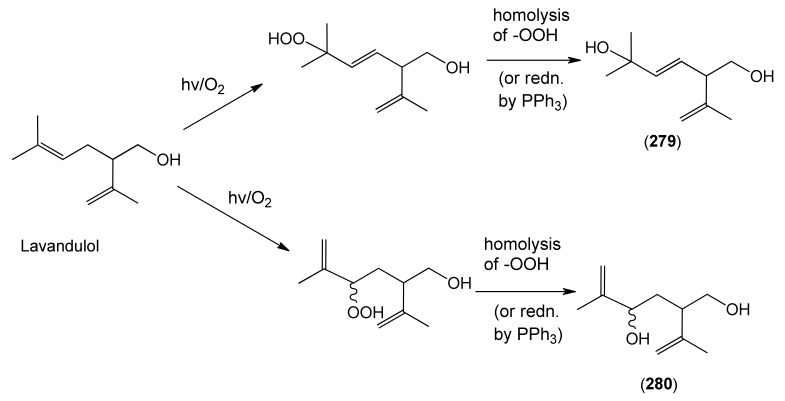
Proposed formation of lavandulanes **279** and **280** by spontaneous autoxidation reactions.

Both the lavandulane tertiary allylic alcohol **279** and its secondary allylic alcohol analogue **280** have been proposed to be derived from the precursor, lavandulol, by spontaneous autoxidation reactions which yield allylic hydroperoxide intermediates as shown in Scheme 3 [129]. This suggestion was supported by a biomimetic synthesis, in which photooxygenation of racemic lavandulol yielded both of the hydroperoxide intermediates expected from the reaction of singlet oxygen with the tri-substituted double bond in this precursor. Reduction of the tertiary allylic hydroperoxide product then resulted in a tertiary allylic alcohol identical with the natural product **279**.

#### 2.5.3. Monocyclic Monoterpenes

Two unusual cyclopentane monoterpeneoids, α-campholenal ([4501-58-0]) (**284**) [19] and cyclopentene, 4-ethenyl-1,5,5-trimethyl- ([1727-69-1]) (**285**) [23] are reported from *A. annua*. Both are probably derived from cleavage reactions of the corresponding bicyclic monoterpenoids (see Section 2.5.4). 2-Ochtoden-1-al [26532-25-2] (**286**) [25] is another unusual six-membered monoterpene, belonging to the ochtodane class of monoterpenoids (3-ethyl-1,1-dimethylcyclohexane, 9CI). The vast majority of monocyclic monoterpenes from *A. annua*, however, are *p*-menthanes (Table 25), incorporating a six-membered ring, which is formed directly by cyclization of an LPP/NPP precursor.

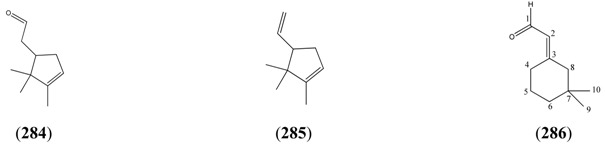



**Table 25:** *p-*Menthane Monoterpenoids, 8CI (1-Methyl-4-(1-methylethyl)cyclohexane, 9CI).

Name	Alternative Name(s)	CAS number	References
*p*-Menth-3-ene (**287**)		[500-00-5]	[50]
*p*-Mentha-2,4-diene (**288**)		[586-68-5]	[25]
α-Phellandrene (**289**)	*p*-Mentha-1,5-diene	[99-83-2]	[32]
β-Phellandrene (**290**)	*p*-Mentha-1(7),2-diene	[555-10-2]	[22, 25, 45]
α-Terpinene (**291**)	*p*-Menthan-1,3-diene	[99-86-5]	[19, 32, 40, 41, 45, 66, 74]
γ-Terpinene (**292**)	*p*-Menthan-1,4,diene	[99-85-4]	[19, 28, 32, 40, 41, 43, 66]
Terpinolene (**293**)	*p*-Mentha-1,4(8)-diene	[586-62-9]	[32, 43]
Limonene (**294**)	*p*-Mentha-1,8-diene	[138-86-3] [5989-27-5]	[24, 31, 34, 43, 45, 50, 66, 111]
*p*-Cymene (**295**)	1-Methyl-4-isopropyl benzene	[99-87-6]	[19, 24, 25, 28, 29, 32, 34, 38, 40, 43, 45, 48, 73, 74]
Cuminic alcohol (**296**)	*p*-Mentha-1,3,5-trien-7-ol 4-Isopropenylbenzyl alcohol	[536-60-7]	[51]
Cuminal (**297**)	Cuminaldehyde *p*-Menthan-1,3,5-trien-1-al 4-Isopropylbenzaldehyde	[122-03-2]	[23, 25, 32, 43, 45]
Carvacrol (**298**)	*p*-Mentha-1,3,5-trien-2-ol	[499-75-2]	[31, 32, 43]
Thymol (**299**)	*p-*Cymen-3-ol *p*-Mentha-1,3,5-trien-3-ol	[89-83-8]	[19, 32, 43]
*p*-Cymen-8-ol (**300**)	2-(4-Methylphenyl)-2-propanol	[1197-01-9]	[19, 25, 32]
Menthol (**301**)	*p*-Menthan-3-ol	[89-78-1]	[23, 25, 30, 60, 111]
β-Terpineol (**302**)	*p*-Menth-8-en-1-ol	[7299-41-4]	[43]
*cis*-*p*-Menth-2-en-1-ol (**303**)		[29803-81-4]	[19, 45]
*trans*-*p*-Menth-2-en-1-ol (**304**)		[29803-82-5]	[19, 45]
*p*-Menth-2,8-dien-1-ol (**305**)	1-Methyl-4-(1-methylethyl)-2-cyclohexen-1-ol	[3886-78-0]	[32]
*trans*-Carveol (**306**)	*p*-Mentha-1,8-dien-6-ol, *trans*-	[1197-07-5]	[19, 25, 41, 43, 45]
*cis*-Carveol (**307**)	*p*-Mentha-1,8-dien-6-ol, *cis*-	[1197-06-4]	[19, 41, 43]
*trans*-Carvyl acetate (**308**)	*p*-Mentha-6,8-dien-2-ol, acetate, *trans*-	[1134-95-8]	[19, 31]
*cis*-Carvyl acetate (**309**)	*p*-Mentha-6,8-dien-2-ol, acetate, *cis*-	[1205-42-1]	[19]
Carvone (**310**)	*p*-Mentha-1,8-dien-6-one	[99-49-0]	[19, 24, 25, 28,32, 43]
*p*-Mentha-1(7),5-dien-2-ol (**311**)		[30681-15-3]	[43]
*p*-Mentha-1(7),8-dien-2-ol (**312**)		[35907-10-9]	[51]
*p*-Menth-1-en-5-ol (**313**)		[55708-42-4]	[22]
*p*-Mentha-1,4(8)-dien-3-ol (**314**)		[6753-08-8]	[32]
3-Cyclohexene-1-methanol 2-hydroxy-α,α,4-trimethyl-, 1-acetate (**315**)		[138913-54-9]	[25]
*Iso*-menthone (**316**)	*p*-Menthan-3-one	[491-07-6]	[45]
Piperitone (**317**)	*p*-Menth-1-en-3-one	[89-81-6]	[24]
Terpinen-4-ol (**318**)	*p*-Menth-1-en-4-ol	[562-74-3]	[19, 30, 31, 32, 34, 41, 43, 45, 51, 66]
4-Terpinyl acetate (**319**)	*p*-Menth-1-en-4-ol acetate	[4821-04-9]	[34]
Phellandral (**320**)	*p*-Menth-1-en-7-al	[21391-98-0]	[23]
Perillaldehyde (**321**)	*p*-Mentha-1,8-dien-7-al	[2111-75-3]	[45]
α-Terpineol (**322**)	l-α-Terpineol *p*-Menth-1-en-8-ol	[98-55-5] [10482-56-1]	[19, 22, 23, 28, 32, 40, 43, 45, 74]
δ-Terpineol (**323**)	*p*-Menthen-1(7)-en-8-ol	[7299-42-5]	[19]
Limonene-1,2-epoxide (**324**)	Limonene oxide 1,2-Epoxy-*p*-menth-8-ene	[1195-92-2]	[23, 51]
1,4-Cineole (**325**)	1,4-Epoxy-*p*-menthane	[470-67-7]	[112]
1,8-Cineole (**326**)	Eucalyptol 1,8-Epoxy-*p*-menthane	[470-82-6]	[19, 22, 23, 24, 25, 28, 29, 32, 33, 34, 36, 37, 38, 40, 41, 43, 45, 48, 49, 50, 51, 53, 66, 72, 73, 74, 112, 113]
2,3-Dihydro-1,8-cineole (**327**)	1,8-Epoxy-*p*-menth-2-ene	[92760-25-3]	[19, 32, 41, 43]
2-α-Hydroxy-1,8-cineole (**328**)		[60761-00-4]	[113]
Ascaridole (**329**)	1,4-Epidioxy-*p-*menth-2-ene	[512-85-6]	[28]
2-Cyclohexen-1-one, 2-methyl-5-(1-methylcyclopropyl)- (**330**)		[26541-44-6]	[43]

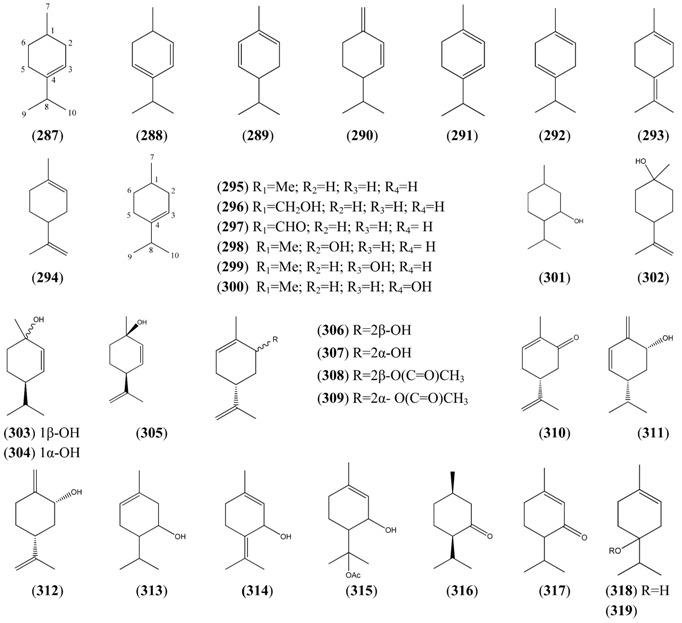
 
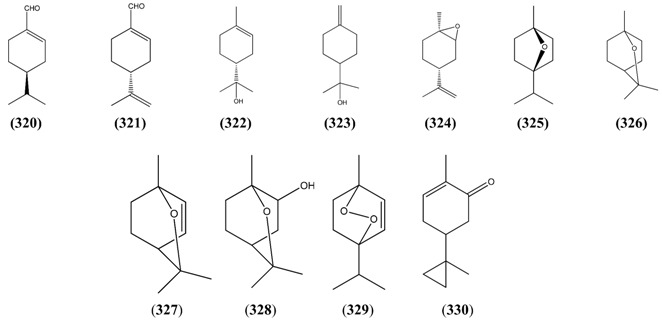

1,8-Cineole (**326**) is the most abundant *p-*menthane monoterpene from *A. annua* [22,29,40,42,43,45,73], accounting for as much as 10-30% of the essential oil [25,28,33,34,41,53]. 1,8-Cineole is sometimes accompanied by significant quantities of *p*-cymene (**295**) [28,34] and α-terpineol (**322**) [28], which could be derived from opening of the ether linkage in 1,8-cineole (**326**).

Only two *o*-menthanes: *o*-cymene (1-isopropyl-2-methylbenzene [527-84-4] [25155-15-1]) (**331**) [41] and *o*-mentha-1(7),8-dien-3-ol ([15358-81-3]) (**332**) [23]; and one *m*-menthane: *m*-cymene (β-cymene; 1-methyl-3-(1-methylethyl)benzene [535-77-3]) (**333**) [31] are reported from *A. annua*. These unusual skeletons presumably arise by migration of a methyl group in the corresponding *p*-menthane precursor (*i.e.* **331** and **333** are perhaps derived from **295**).

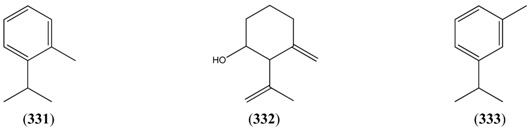



#### 2.5.4. Bicyclic Monoterpenes

Further cyclization of the carbocation which produces the *p*-menthane skeleton results in bicyclic monoterpenes. In the camphane monoterpenes listed in Table 26, the second cyclization has produced an additional five-membered ring. The most abundant member of this class of monoterpenoids is camphor (**341**) [25,29,37,45], which can account for up to 10-30% of the essential oil [19,22,41,42]. Borneol (**334**) [46,73] and bornyl acetate (**335**) [29] are sometimes reported together with camphor (**351**) as significant constituents of the essential oil.

**Table 26:** Camphane Monoterpenoids (1,7,7-Trimethylbicyclo-[2.2.1]heptane, 9CI).

Name	Alternative Name(s)	CAS Number	References
Borneol (**334**)		[464-43-7] [507-70-0] [124-76-5]	[19, 25, 30, 31, 34, 40, 41, 43, 45, 72, 73, 111, 112]
Bornyl acetate (**335**)		[76-49-3] [92618-89-8]	[25, 31, 40, 45]
Borneol isobutyrate (**336**)		[24717-86-0]	[45]
Bornyl valerate (**337**)	Bornyl pentanoate	[7549-41-9]	[31]
2-Butenoic acid, 3-methyl-(1*S*,2*R*,4*S*)-1,7,7-trimethylbicyclo[2.2.1]hept-2-yl ester (**338**)		[91404-82-9]	[31]
Cyclopentanecarboxylic acid, 3-methylene-, 1,7,7-trimethylbicyclo-[2.2.1]hept-2-yl ester (**339**)		[74793-59-2]	[31]
Isobornyl acetate (**340**)	2-Bornanol acetate	[125-12-2]	[19]
Camphor (**341**)	1,7,7-Trimethylbicyclo[2.2.1] heptan-2-one	[76-22-2] [464-48-2]	[19, 23, 25, 26, 29, 32, 34, 36, 37, 38, 40, 41, 42, 43, 44, 45, 48, 49, 51, 53, 66, 72, 73, 74, 112, 113]
*endo*-Dehydronorborneol (**342**)	Bicyclo[2.2.1]hept-5-en-2-ol	[694-97-3]	[23]

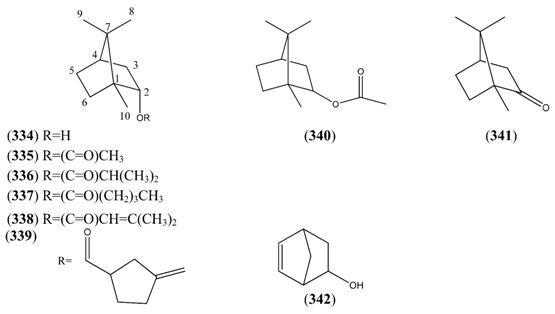

Other miscellaneous bicyclic monoterpenoids which contain both 5- and 6-membered rings are: camphene (2,2-dimethyl-3-methylenebicyclo[2.2.1]heptane [79-92-5]) (**343**) [19,23,25,26,28,29,31,32, 34,36,37,40,41,43,45,51,73,74,130]; camphene hydrate ([465-31-6]) (**344**) [19]; fenchol (fenchyl alcohol; 1,3,3-trimetylbicyclo[2.2.1]heptan-2-ol [1632-73-1]) (**345**) [66]; and *trans*-pinene hydrate (2-norbornanol, 2,7,7-trimethyl-, endo-; bicyclo[2.2.1]heptan-2-ol, 2,7,7-trimethyl-, endo-; [3247-40-3]) (**346**) [25]. Camphene (**343**) has been obtained as both its (+)- and (-)-enantiomeric forms [37].

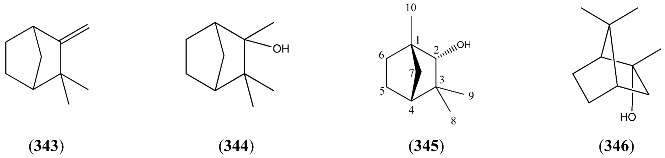



In the pinane monoterpenes (Table 27), the second cyclization of the C_10_ *p*-menthane precursor has produced an additional four-membered ring. α-Pinene (**347**) [40,42,43] is the most abundant pinane monoterpene from *A. annua* (up to 10%) [33], and sometimes co-occurrs with significant amounts of either β-pinene (**356**) [101] or pinocarvone (**360**) [42]. A β-pinene synthase, which converts GPP to both (-)-α-pinene (**347**) and (-)-β-pinene (**356**), has recently been described from *A. annua* [131]. α-Pinene (**347**) is known in both its (+)- and (-)-enantiomeric forms from this species [37].

**Table 27:** Pinane Monoterpenoids (2,6,6-Trimethylbicyclo[3.1.1]heptane, 9CI).

Name	Alternative Name(s)	CAS Number	References
α-Pinene (**347**)	2,6,6-Trimethylbicyclo[3.1.1]hept-2-ene	[80-56-8] [7785-26-4] [7785-70-8]	[19, 22, 25, 28, 29, 32, 33, 34, 36, 37, 38, 40, 41, 42, 43, 44, 45, 50, 51, 66, 73, 74, 112, 113, 120, 132]
Verbenyl acetate (**348**)	Verbenol acetate 2-Pinen-4-ol, acetate	[33522-69-9]	[23]
*trans*-Chrysanthenol (**349**)		[38043-83-3]	[43]
*cis*-Chrysanthenyl acetate (**350**)		[67999-48-8]	[19]
Myrtenol (**351**)	2-Pinen-10-ol	[515-00-4] [564-94-3]	[19, 23, 32, 41, 43, 66]
(-)-Myrtenyl acetate (**352**)	(-)-*O*-Acetylmyrtenol	[36203-31-3]	[23]
Verbenone (**353**)	2-Pinen-4-one	[80-57-9]	[19, 25, 34, 43, 45]
Chrysanthenone (**354**)	2-Pinen-6-one	[473-06-3]	[32, 43, 66]
Myrtenal (**355**)	2-Pinen-10-al	[23727-16-4]	[66]
β-Pinene (**356**)	6,6-Dimethyl-2-methylenebicyclo[3.1.1]-heptane	[127-91-3] [1330-16-1]	[19, 22, 23, 25, 26, 27, 28, 32, 33, 34, 36, 38, 40, 43, 45, 51, 66, 73, 74, 112, 113, 120, 132]
(-)-*trans*-Pinocarveol (**357**)	2(10)-Pinen-3-ol	[547-61-5] [3917-59-7]	[19, 23, 25, 32, 33, 40, 43]
*cis*-Pinocarveol (**358**)	Isopinocarveol 2(10)-Pinen-3-ol, *cis*-	[6712-79-4] [5947-36-4]	[30, 43]
Pinocarvyl acetate (**359**)	2(10)-Pinen-3-ol, acetate	[1078-95-1]	[30]
Pinocarvone (**360**)	2(10)-Pinen-3-one	[30460-92-5] [19890-00-7]	[19, 23, 25, 28, 30, 32, 33, 42, 43, 45, 66]
3-Pinanol (**361**)	2,6,6-Trimethylbicyclo[3.1.1]heptan-3-ol	[25465-95-6]	[30]
β-Pinene oxide (**362**)	2,10-Epoxypinane	[6931-54-0]	[19]
Bicyclo[3.1.1]heptan-3-one, 2,6,6-trimethyl-4-methylene- (**363**)		[62594-31-4]	[30]

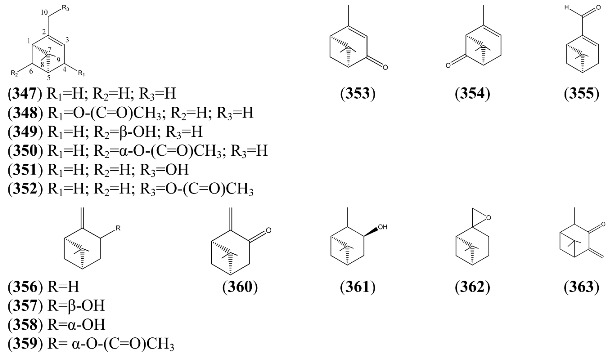

Only two caranes (3,7,7-trimethylbicyclo[4.1.0]heptane, 9CI): α-carene ([13466-78-9]) (**364**) [32,41] and 2-acetyl-3-carene ([40824-36-0]) (**365**) [23] are known from *A. annua*: the majority of monoterpenes which incorporate an additional three-membered ring belong to the thujane class (Table 28). Two of the most abundant thujanes are sabinene hydrate (**373**) [34] and sabinol (**371**) [43], both of which can be present at around levels of 7% of the total essential oil.

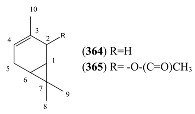



**Table 28:** Thujane Monoterpenoids (4-Methyl-1-(1-methylethyl)-bicyclo[3.1.0]hexane, 9CI).

Name	Alternative Name(s)	CAS Number	References
α-Thujene (**366**)	3-Thujene 2-Methyl-5-(1-methylethyl)bicyclo[3.1.0]hex-2-ene	[2867-05-2]	[28, 34, 41, 43, 45, 66]
3-Thujen-2-ol (**367**)	Bicyclo[3.1.0]hex-3-en-2-ol, 4-methyl-1-(1-methylethyl)	[3310-03-0]	[25]
3-Thujen-10-al (**368**)		[57129-54-1]	[50]
(-)-α-Thujone (**369**)		[546-80-5]	[45, 66, 111]
Sabinene (**370**)	4(10)-Thujene Bicyclo[3.1.0]hexane,4-methylene-1-(1-methylethyl)-	[2009-00-9] [3387-41-5] [204524-73-2]	[19, 23, 31, 32, 34, 40, 41, 43, 45, 51, 66, 74, 113]
Sabinol (**371**)	4(10)-Thujen-3-ol	[471-16-9]	[32, 43]
*trans*-Sabinyl acetate (**372**)	Bicyclo[3.1.0]hexan-3-ol, 4-methylene-1-(1-methylethyl)-, 3-acetate	[139757-62-3] [3536-54-7]	[43]
β-Sabinene hydrate (**373**)	4-Thujanol	[546-79-2] [15537-55-0] [17699-16-0]	[19, 25, 32, 34,41, 43, 45]
Sabina ketone (**374**)	Didehydrosabina ketone Bicyclo[3.1.0]hexan-2-one, 5-(1-methylethyl)-, 5-Isopropyl- bicyclo[3.1.0]hexan-2-one	[513-20-2] [110716-99-9] [147043-52-5]	[19, 43]

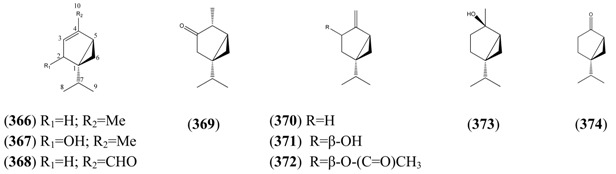

Tricyclene ([508-32-7] [160339-05-9]) (**375**) [25,32,41,43,45] is the only tricyclic monoterpenoid known from *A. annua*. 

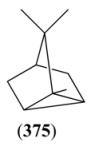



### 2.6. Sesquiterpenoids

Sesquiterpenoids (C_15_) constitute the most abundant and most diverse group of natural products from *A. annua*. All are produced from farnesyl pyrophosphate (FPP; **378**), which is the product of a “head-to-tail” condensation of three C_5_ units (IPP and DMAPP; see Figure 2). Sesquiterpene hydrocarbons are generally more volatile than their highly oxygenated counterparts and are more suited to study by GC-MS (*cf*. monoterpenes in Section 2.5, which are the other major components of the essential oil from *A. annua*). More highly oxygenated sesquiterpenes, such as artemisinin (**495**), are best analysed by liquid chromatography. 

#### 2.6.1. Farnesane Sesquiterpenes

The farnesanes are the structurally simplest group of sesquiterpenes. This acyclic group of natural products is produced by ionization of the pyrophosphate group in the 15-carbon precursor, FPP (**378**), followed either by quenching with water or loss of a proton, which results in the variety of structures shown in Table 29. Alternatively, FPP can undergo further cyclization to mono-cyclic sesquiterpenes (Section 2.6.2), bicyclic sesquiterpenes (Section 2.6.3) or tricyclic sesquiterpenes (Section 2.6.4), according to enzymatically-catalysed mechanisms which bear close analogies with the biosynthesis of the cyclized monoterpenoids (Section 2.5.3 and Section 2.5.4).

β-Farnesene (**382**) is the most abundant member of this class of sesquiterpenes [22,43], constituting up to 10% of the essential oil [19,27]. A cDNA clone encoding (*E*)-β-farnesene synthase, which catalyzes the formation of β-farnesene (**382**) from FPP (**378**), has recently been isolated from *A. annua* [133]. Interestingly, the enzyme amorpha-4,11-diene synthase, which catalyses the formation of the bicyclic sesquiterpene amorpha-4,11-diene, (Section 2.6.3) also produces small amounts of acyclic sesquiterpenes such as *trans*-nerolidol (**380**) and (*E*)-1,3(15),6,10-farnesatetraene (**382**), both of which are reported as natural products from *A. annua* [134] (Table 29).

**Table 29:** Farnesane Sesquiterpenoids (2,6,10-Trimethyldodecane, 9CI).

Name	Alternative Name(s)	CAS Number	References
Farnesol (**376**)	2,6,10-Farnesatrien-1-ol 3,7,11-Trimethyl-2,6,10-dodecatrien-1-ol	[4602-84-0] [106-28-5]	[23, 27, 135]
2,6,10-Farnesatrien-1-ol acetate (**377**)		[4128-17-0]	[27]
Farnesyl pyrophosphate (**378**)	Farnesyl diphosphate 2,6,10-Dodecatrien-1-ol, 3,7,11-trimethyl-, trihydrogen pyrophosphate Diphosphoric acid, mono (3,7,11-trimethyl-2,6,10-dodecatrienyl) ester	[13058-04-3]	[135, 136]
Farnesal (**379**)	2,6,10-Farnesatrien-1-al 3,7,11-Trimethyl-2,6,10-dodecatrienal	[19317-11-4]	[27]
*trans*-Nerolidol (**380**)	3,6,10-Farnesatrien-3-ol 3,7,11-Trimethyl-1,6,10-dodecatrien-3-ol	[7212-44-4] [3790-78-1]	[19, 23, 27, 32, 43, 45, 134]
(*E*)-Nerolidyl acetate (**381**)	(*E*)-Nerolidol acetate 1,6,10-Dodecatrien-3-ol, 3,7,11-trimethyl-, 3-acetate	[85611-33-2]	[27]
*trans*-β-Farnesene (**382**)	(*E*)-1,3(15),6,10-Farnesatetraene (*E*)-7,11-Dimethyl-3-methylene-1,6,10-dodecatriene	[77129-48-7] [18794-84-8]	[19, 22, 27, 30, 32, 43, 48, 50, 51, 66, 134, 135]
(*Z*)-1,3(15),6,10-Farnesatetraene (**383**)		[28973-97-9]	[23, 31, 32]
α-Farnesene (**384**)	1,3,6,10-Farnesatetraene 3,7,11-Trimethyl-1,3,6,10-dodecatetraene	[502-61-4] [125037-13-0]	[27, 41, 48, 50, 74, 111]

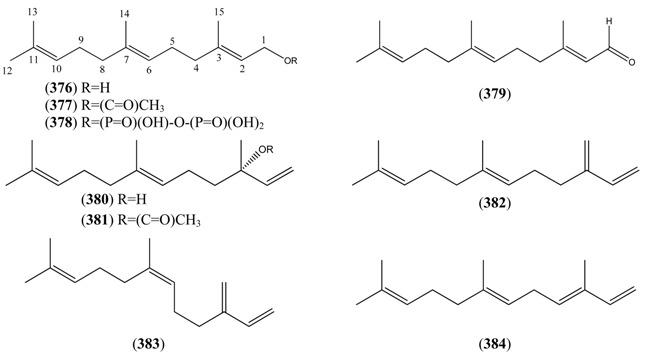

#### 2.6.2. Monocyclic Sesquiterpenes

The six-membered ring in the bisabolane sesquiterpenes from *A. annua* (Table 30) is formed by cyclization of the C_15_ precursor, FPP (**378**), in much the same way that the *p*-menthane monoterpenes (Section 2.5.3) arise from the corresponding C_10_ precursor. Alternative cyclizations of FPP can lead to the ten-membered germacrane sesquiterpenes, which are described in Table 31, and the eleven-membered humulane sesquiterpenes in Table 33. The bicyclic caryophyllanes, which are formed by a further cyclization of a humulane precursor, have also been included in Table 33.

##### 2.6.2.1. Bisabolanes

**Figure 3:**
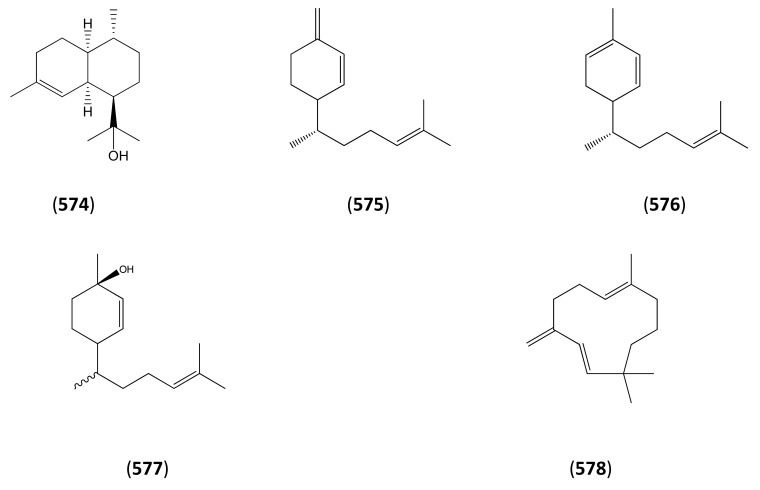
Minor products from the cyclization of FPP (**378**) by ADS, which have not yet been reported as natural products from *A. annua*. Amorph-4-en-11-ol (**574**) is probably formed by quenching of the C-11 amorphane cation by water; β-sesquiphellandrene (**575**) by elimination of H-15 from the C-1 bisabolyl cation; zingiberene (**576**) from elimination of H-4 from the C-1 biasabolyl cation; zingiberenol (**577**) by quenching of the allylic C-1 bisabolyl cation by water; and γ-humulene (**578**) by an alternative cyclization of nerolidyl pyrophosphate.

α- and β-Bisabolene are the most abundant bisabolane sesquiterpenes from *A. annua*, constituting up to 5% of the essential oil [28,46]. The bisabolane sesquiterpene, α-bisabolol (**385**), has been found as a minor product from the enzyme amorpha-4,11-diene synthase from *A. annua* (Section 2.6.3) [136]. Several other bisabolanes, which have not yet been reported as natural products from *A. annua*, were also obtained from the cyclization of FPP which is catalysed by this enzyme [134] [these included zingiberene; β-sesquiphellandrene; and zingiberenol (see Figure 3, Section 3.1)].

**Table 30:** Bisabolanes Sesquiterpenoids (1-(1,5-Dimethylhexyl)-4-methylcyclohexane, 9CI).

Name	Alternative Name(s)	CAS Number	References
α-Bisabolol (**385**)		[515-69-5]	[31, 32, 43, 45, 134]
*cis-*Lanceol (**386**)	2,7(14),10-Bisabolatrien-12-ol	[147129-37-1]	[46]
2,7,10-Bisabolatriene (**387**)		[58845-44-6]	[28]
2,3-Epoxy-7,10-bisaboladiene (**388**)		[111536-37-9]	[23]
7-Oxabicyclo[4.1.0]heptane, 4-(1,5-dimethyl-4-hexen-1-ylidene)-1-methyl-, (1*R*,4*Z*,6*S*)- (**389**)		[94347-02-1]	[31]

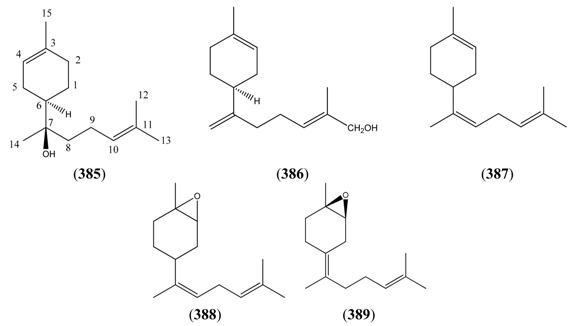

##### 2.6.2.2. Germacranes and Elemanes

Germacrene D (**392**) is the most abundant member of the germacrane class of sesquiterpenes [22,37,46] and can constitute between 5-20% of the essential oil [19,42,43]. Germacrene A synthase, the enzyme which catalyzes the cyclization of FPP (**378**) to germacrene A (**390**), has recently been isolated from the glandular trichomes of *A.* a*nnua* [137]. Compound (**395**) ([24703-35-3]) is the only example of a bicyclogermacrane sesquiterpene so far described from *A. annua* [19,32,43,45,74]. 

**Table 31:** Germacrane Sesquiterpenoids (1,7-Dimethyl-4-(1-methylethyl)cyclodecane, 9CI).

Name	Alternative Name(s)	CAS Number	References
(+)-Germacrene A (**390**)	Germacrene (1(10)*E*,4(*E*))-Germacra-1(10),4,11-triene	[28028-64-0]	[74]
Germacrene B (**391**)	(1(10)*E*,4(E)-1(10),4,7(11)-Germacratriene	[15423-57-1]	[23, 28, 46]
Germacrene D (**392**)	1(10),4(15),5-Germacratiene	[23986-74-5]	[19, 23, 31, 32, 33, 36, 37, 42, 43, 45, 48, 66, 135]
1β-Hydroxy-4(15),5(*E*),10(14)-germacratriene (**393**)			[9]
Pregeijerene (**394**)	11,12,13-tri-*nor*-1(10),4,6,-Germacratriene 1,5-Dimethyl-1,5,7-cyclodecatriene	[20082-17-1]	[41]

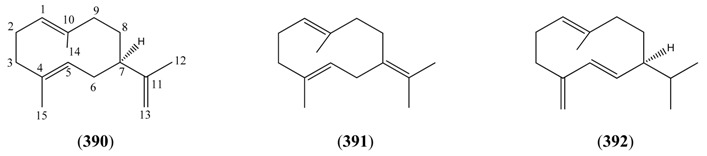
 
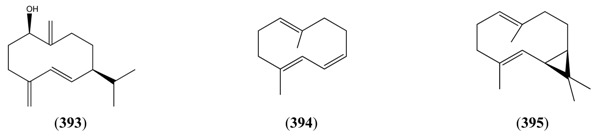

The ten-membered germacrane ring frequently occurs with unsaturation at the 1(10) and 4-positions, as is apparent from Table 31. The six-membered ring in the elemane sesquiterpenes (Table 32) is thought to be formed by Cope rearrangement of such 1(10),4-germacradienes. Elemanes may thus be artifacts which are introduced during the extraction and isolation process, especially, perhaps, as a result of the high temperatures often associated with GC-MS analysis (e.g., **397** may be formed thermally from **391**; and **396** may be formed from **390** by just such a pericyclic reaction).

**Table 32:** Elemane Sesquiterpenoids (1-Ethyl-1-methyl-2,4-bis-(1-methylethyl)cyclohexane, 9CI).

Name	Alternative Name(s)	CAS Number	References
β-Elemene (**396**)	1,3,11-Elematriene	[33880-83-0] [515-13-9]	[27, 31, 34, 40, 43, 45]
γ-Elemene (**397**)	1,3,7(11)-Elematriene	[29873-99-2] [3242-08-8]	[23, 28, 31]
δ-Elemene (**398**)	1,3,6-Elemantriene	[20307-84-0]	[32, 48]
Elemol (**399**)	1,3-Elemadien-11-ol	[639-99-6]	[27]
Elemyl acetate (**400**)	Elemol acetate	[60031-93-8]	[19]

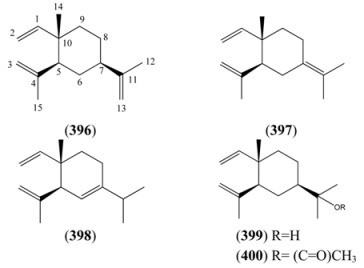

##### 2.6.2.3. Humulanes and Caryophyllanes

β-Caryophyllene (**405**) is the most abundant member of this group of sesquiterpenes [26,37,40,43,46] and can be found at levels of up to 5-10% of the total essential oil [19,22,42]. A cDNA clone for β-caryophyllene synthase, the sesquiterpene cyclase which converts FPP (**378**) to β-caryophyllene (**405**) has now been isolated from *A. annua* [139]. The β-caryophyllene synthase gene was found to be expressed widely in most plant tissues during early development, and could be induced in mature tissue in response to a fungal elicitor. This was interpreted as evidence that β-caryophyllene (**405**) might play a role in plant defense [139]. The enzyme amorpha-4,11-diene synthase from *A. annua* has been observed to produce small amounts of γ-humulene (isomeric with α-humulene (**401**) in Table 33 – see also Figure 3, Section 3.1), as well as the expected bicyclic product [134].

**Table 33:** Humulane Sesquiterpenoids (1,1,4,8-Tetramethylcycloundecane, 9CI) and Caryophyllane Sesquiterpenoids (2,6,10,10-Tetramethylbicyclo[7.2.0]undecane, 9CI).

Name	Alternative Name(s)	CAS Number	References
*Humulanes*			
α-Humulene (**401**)	2,6,9-Humulatriene	[6753-98-6]	[19, 27, 28, 32, 40, 43, 66]
14-Hydroxy-α-humulene (**402**)		[108043-85-2]	[27]
Humulene epoxide I (**403**)	2,3-Epoxy-6,9-humuladiene	[19888-33-6]	[45]
Humulene epoxide II (**404**)	6,7-Epoxy-2,9-humuladiene	[19888-34-7]	[27, 45]
*Caryophyllanes*			
β-Caryophyllene (**405**)	(*E*)-3(15),6-Caryophylladiene	[87-44-5]	[19, 22, 23, 24, 26, 27, 29, 32, 36, 37, 40, 41, 42, 43, 45, 48, 49, 51, 66, 72, 73, 74, 112, 140]
γ-Caryophyllene (**406**)	Isocaryophyllene (*Z*)-3(15),6-Caryophylladiene	[118-65-0]	[31]
(1*R*,3*Z*,9*S*)-Bicyclo[7.2.0]undec-3-ene, 4,11,11-trimethyl-8-methylene- (**407**)		[136296-35-0]	[50]
Caryophylladienol I (**408**)	Caryophyllenol Bicyclo[7.2.0]undecan-5-ol, 10,10-dimethyl-2,6-bis(methylene)-, (1*S*,5*S*,9*R*)-Caryophylla-4(12),8(13)-dien-5β-ol	[19431-80-2] [38284-26-3]	[28, 32, 43]
Caryophylladienol II (**409**)	Caryophyllenol Bicyclo[7.2.0]undecan-5-ol, 10,10-dimethyl-2,6-bis(methylene)-, (1*S*,5*R*,9*R*)-Caryophylla-4(12),8(13)-dien-5α-ol	[19431-79-9] [38284-26-3]	[28, 32, 43]
Caryophyllene oxide (**410**)	Isocaryophyllene oxide 6,7-Epoxy-3(15)-caryophyllene	[1139-30-6] [113877-94-6] [17627-43-9]	[9, 19, 23, 25, 27, 30, 32, 34, 43, 45, 48, 49, 50, 66]
*cis*-Caryophyllene oxide (**411**)	5-Oxatricyclo[8.2.0.04,6]dodecane, 4,12,12-trimethyl-9-methylene-, (1*R*,4*S*,6*R*,10*S*)-	[60594-23-2]	[32]

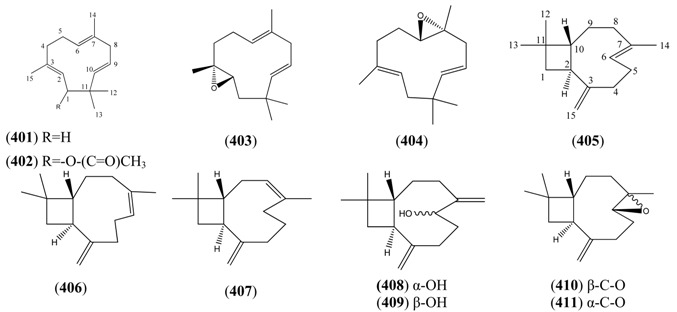

#### 2.6.3. Bicyclic Sesquiterpenes

##### 2.6.3.1. Eudesmanes and Eremophilanes

**Scheme 4:**
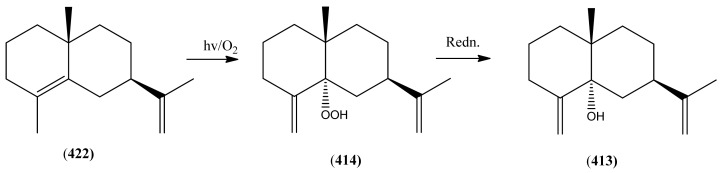
Postulated biogenesis of (**414**) and (**413**) by spontaneous autoxidation reaction of (**422**), followed by reduction.

The bicyclic eudesmane sesquiterpenes (Table 34) are thought to be derived from further cyclization of the monocyclic germacranes (Table 31, Section 2.6.2). Eudesmanes are sometimes referred to as selinanes in the older literature and the most abundant eudesmane from *A. annua* is β-selinene (**412**) [40,42]. The isolation of the eudesmane allylic hydroperoxide (**414**) from *A. annua* is significant, as its structure is suggestive of formation by spontaneous autoxidation of the hydrocarbon precursor selina-4,11-diene (**422**) [9]. It has been suggested that reduction of the hydroperoxide in **414** would then result in the allylic alcohol group observed in the natural product 5α-hydroxy-eudesma-4(15),11-diene (**413**), as shown in Scheme 4 [9].

**Table 34:** Eudesmane (Selinane) Sesquiterpenoids (Decahydro-1,4α-dimethyl-7-(1-methylethyl)-naphthalene, 9CI).

Name	Alternative Name(s)	CAS Number	References
β-Selinene (**412**)	4(15),11-Eudesmadiene	[17066-67-0]	[19, 23, 28, 30, 31, 32, 34, 40, 42, 43, 48, 113]
5α-Hydroxy-eudesma-4(15),11-diene (**413**)	4(15),11-Eudesmadien-5α-ol		[9, 97]
5α-Hydroperoxy-eudesma-4(15),11-diene (**414**)			[9]
1β,6α-Dihydroxy-4(15)-eudesmane (**415**)			[9]
1β-Hydroxy-4(15),5-eudesmadiene (**416**)			[9]
1β-Hydroxy-4(15),7-eudesmadiene (**417**)			[9]
γ-Selinene (**418**)	4(15),7(11)-Eudesmadiene 4(15),7(11)-Selinadiene	[515-17-3]	[45, 48]
β-Eudesmol (**419**)	4(15)-Eudesmen-11-ol	[473-15-4]	[45]
α-Selinene (**420**)	(5α,7β,10β)-α-Eudesmane 3,11-Eudesmadiene Selina-3,11-diene	[473-13-2]	[34, 111]
Kongol (**421**)	11-Eudesmen-4-ol(4α,5α,7β,10β) Selin-11-en-4α-ol	[16641-47-7]	[19, 27]
Selina-4,11-diene (**422**)	Eudesma-4,11-diene	[17627-30-4]	[135]
γ-Eudesmol (**423**)	4-Eudesmen-11-ol	[1209-71-8]	[27]
10-*epi*-γ-Eudesmol (**424**)	4-Eudemen-11-ol (7β,10α)	[15051-81-7]	[27, 45]
Occidentalol (**425**)	1,3-Eudesmadien-11-ol	[473-17-6]	[27]
Occidentalol acetate (**426**)		[346608-97-7]	[27]
Occidol (**427**)	1,2,3,4-Tetrahydro-α,α-5,8-tetramethyl-2-naphthalenemethanol	[5986-36-7]	[27]
Artemisin (**428**)			[141]
α-Hydroxysantonin (**429**)			[142]

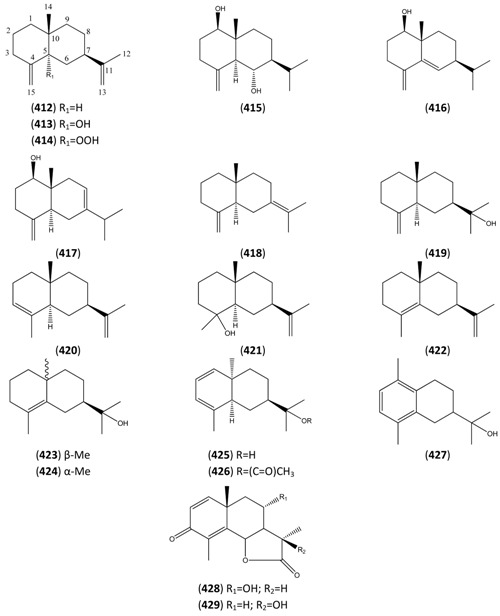

The eremophilane skeleton (decahydro-1,8a-dimethyl-7-(1-methylethyl)naphthalene, 9CI) is thought to be derived from the eudesmane skeleton by migration of the C-10 methyl group to C-5 [16]. Two eremophilanes: nootkatone (1(10),11-eremophiladiene-2-one [4674-50-4]) (**430**) [22,43] and valencene (1(10),11-eremophiladiene (4α,5α,7β) [4630-07-3]) (**431**) [28] are currently reported from *A. annua*. The aristolane sesquiterpenoid, β-gurjunene (1(10)-aristolene [73464-47-8]) (**432**) [34], is the only example of a 6,11-cycloeremophilane from this species.

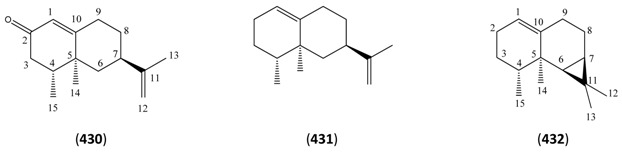



##### 2.6.3.2. Cadinanes, Muurolanes and Amorphanes

The amorphane/cadinane group of bicyclic sesquiterpenes is by far the largest class of sesquiterpenes found in *A. annua*. Unfortunately, the nomenclature and stereochemistry reported for cadinanes and amorphanes in the literature has sometimes become quite confusing. In this review, the relative stereochemistry at the 1-, 6- and 7-positions is used to define four skeletal types, according to guidelines which are set out in the Dictionary of Natural Products [16]. Thus, in the cadinane skeleton, the decalin ring is *trans*-fused (1α,6β,7β), while in the muurolane skeleton (1β,6β,7β), it is *cis*-fused. The cadinane and muurolane sesquiterpenes found in *A. annua* have been grouped together in Table 35. Amorphane sesquiterpenes also incorporate a *cis*-decalin ring junction, but differ from the muurolane sesquiterpenes in their relative stereochemistry at the 7-position. The very large group of amorphane sesquiterpenes from *A. annua* is listed in Table 36. (No representative of the bulgarane sesquiterpenes (1α,6β,7α), the fourth possible skeleton allowed by this classification scheme, is known from *A. annua*).

**Table 35:** Cadinane and Muurolane Sesquiterpenoids (Decahydro-1,6-dimethyl-4-(1-methylethyl)-naphthalene, 9CI).

Name	Alternative Name(s)	CAS number	References
*Cadinanes*			
Artemisinol (**433**)	12-Cadinanol	[82890-78-6]	[143]
δ-Cadinene (**434**)	1(10),4-Cadinadiene	[483-76-1]	[19, 23, 30, 31, 32, 40, 43, 48, 51]
14-Hydroxy-δ-cadinene (**435**)		[153408-92-5]	[27]
4(15),5,11-Cadinatriene (**436**)	1-*epi*-Bicyclosesquiphellandrene	[54274-73-6]	[48]
α-Cadinene (**437**)	4,9-Cadinadiene	[24406-05-1]	[111, 130]
β-Cadinene (**438**)	3,9-Cadinadiene	[523-47-7]	[22, 66]
γ-Cadinene (**439**)	4(10),15-Cadinadiene	[39029-41-9]	[32, 34, 40, 43]
α-Cadinol (**440**)	4-Cadinen-10-ol	[481-34-5]	[19, 43, 45]
γ-Cadinol (**441**)	2-Naphthalenol, 1,2,3,4,4a,7,8,8a-octahydro-2,5-dimethyl-8-(1-methylethyl)-	[50895-55-1]	[45]
*cis*-Calamenene (**442**)		[72937-55-4]	[32, 43]
Cubenol (**443**)	4-Cadinen-1-ol	[21284-22-0]	[19, 43]
*epi*-Cubenol (**444**)	4-Muurolen-1-ol	[19912-67-5]	[41]
*Muurolanes*			
γ-Muurolene (**445**)	4,10(14)-Muuroladiene	[30021-74-0]	[43]
δ-Muurolene (**446**)	4(15),10(14)-Muuroladiene	[1136-29-4]	[23]
4-Muurolen-10-ol (**447**)	Cedrelanol	[5937-11-1]	[25]
*t*-Muurolol (**448**)	4-Muurolen-10-ol (1β, 6β, 7β,10β)	[19912-62-0]	[19]

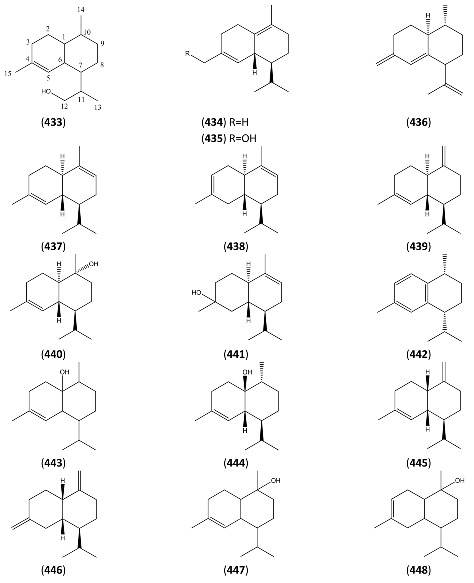

Both the synthesis [144] and NMR properties [145,146] of amorphane and cadinane sesquiterpenes from *A. annua* have been reviewed. Artemisinic acid (**473**) [105,147,148], arteannuin B (**462**) [105,147,149] and artemisinin (**495**) [105,147,150] are the most abundant representatives of this class of natural products and were amongst the first sesquiterpenes to be reported from *A. annua*. Several of the amorphane sesquiterpenes in Table 36 have since been implicated as biosynthetic precursors to artemisinin (**495**), which has been classified as a *seco*-cadinane in Table 37 (the prefix “*seco*-“ indicates that carbon-carbon bond cleavage has occurred – in this case between C-4 and C-5).

The amorphane sesquiterpene, artemisinic acid (**473**) (Table 36) was first isolated in 1981 by Prof Tu’s group [151]. Its structure was confirmed both by X-ray crystallography [148,152] and by NMR spectroscopy [153]; and subsequently by synthesis [154,155]. Depending on the chemotype of *A. annua* being studied, artemisinic acid (**473**) can be present at ten times the concentrations of artemisinin (**495**). For this reason, much research has been undertaken into the chemical conversion of artemisinic acid (**473**) to artemisinin (**495**), which can be achieved with an efficiency of greater than 40% [156,157] (see also Section 4.4 for an application of this conversion to the production of artemisinin). By varying the conditions for the oxidation step, artemisinic acid (**473**) can be converted to various other sesquiterpenes from *A. annua*, including: arteannuin B (**462**) [158, 159], deoxyarteannuin B (**477**) [160] and *epi-*deoxyarteannuin B (**478**) [161,162] (note that deoxyarteannuin B (**477**) [129,161], *epi*-deoxyarteannuin (**478**) [68,158,160,163,164] and 6, 7-dehydroartemisinic acid (**476**) [165] have all also been obtained independently by chemical synthesis).

**Scheme 5:**
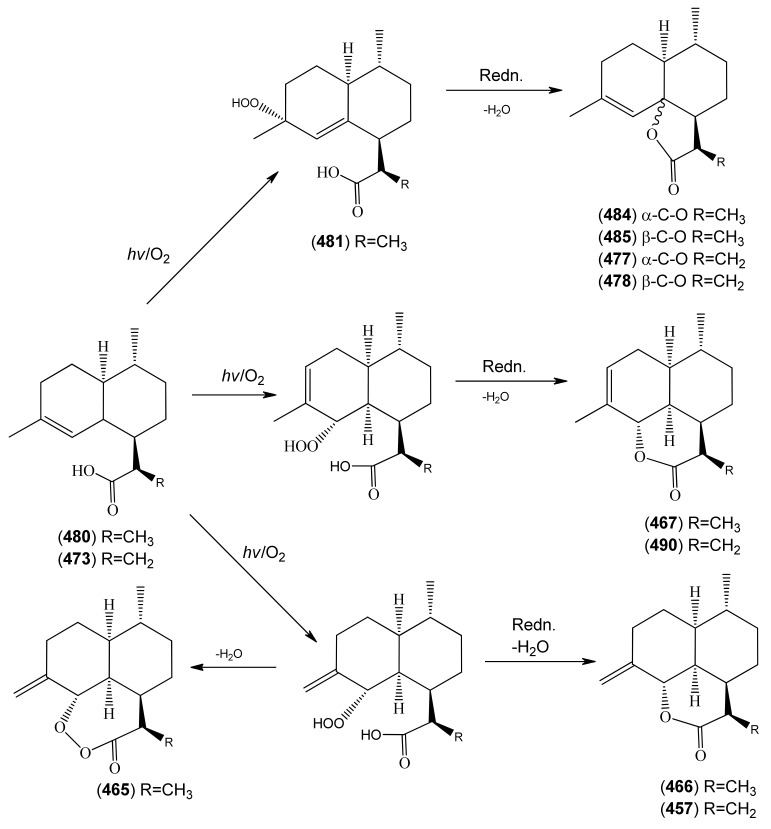
Proposed biogenesis of arteannuin H (**465**) and other amorphane sesquiterpenes from *A. annua* via tertiary and allylic secondary hydroperoxides which are derived from spontaneous autoxidation of dihydroartemisinic acid (**480**)/artemisinic acid (**473**).

Dihydroartemisinic acid (**480**), which is the 11,13-dihyro analogue of artemisinic acid (**473**) in Table 36, was first isolated as a natural product several years after artemisinic acid [166,167] and it has also been chemically synthesised [168,169]. It is particularly significant that dihydroartemisinic acid hydroperoxide (**481**), the tertiary allylic hydroperoxide from dihydroartemisinic acid, has also been isolated as a natural product from *A. annua* [170]. This has led to the suggestion that dihydroartemisinic acid (**480**) might be converted to its tertiary hydroperoxide (**481**) in the living plant by a non-enzymatic process as shown in Scheme 5. This hypothesis has apparently been confirmed by recent *in vivo* and *in vitro* experiments [155,185] (see Section 3.3) which also suggested that (**481**) can undergo further non-enzymatic conversion to (**495**).

Although both artemisinic acid (**473**) and dihydroartemisinic acid (**480**) are the most signifcant amorphane sesquiterpenes from *A. annua* in regard of the biosynthesis of artemisinin (**495**), several other amorphanes from this species have also been implicated in this process (see Section 3.3). These amorphane sesquiterpenes appear amongst the alphabetical listings in Table 36. Arteannuin A (**461**) was one of the first sesquiterpenes to be reported from *A. annua* and it has since been synthesized on two occasions [171,172]. The structure of arteannuin B (**462**) was determined in 1972 by X-ray crystallography [173] in combination with 1D- [174] and 2D- [175] NMR spectroscopy, and it has also been confirmed by chemical reactions [176]. Several syntheses of arteanuin B are reported [158,171,177,178,179171,177-179] and arteannuin C [180] is now thought to be identical with arteannuin B [175]. Syntheses have also been reported of arteannuin E (**463**) [181] and arteannuin F (**464**) [182] (which is also referred to as artemisilactone) [171]. 

The structures of arteannuin H (**465**) [166,183], arteannuin I (**466**) [166], arteannuin J (**467**) [166], arteannuin K (**468**) [166,184], arteannuin L (**469**) [166,184] and arteannuin M (**470**) [166,184] were all deduced by 2D-NMR spectroscopy, when they were first described as natural products from *A. annua*. All of these compounds have been reported as metabolites of dihydroartemisinic acid (**480**) *in vivo* (Scheme 5) [185], and there is evidence from *in vitro* studies to support the biogenetic proposal that arteannuin H (**465**) might be produced by spontaneous autoxidation reactions involving a secondary allylic hydroperoxide, which is derived from dihydroartemisinic acid (**480**) as shown in Scheme 5 [183]. The stereochemistry of the 5-hydroxyl group was wrongly assigned when arteannuins K (**468**), L (**469**) and M (**470**) were first reported as natural products [166]. The correct stereochemistry at the 5-OH group has now been established as α (as drawn) by 2D-NMR studies involving derivitization of synthetic arteannuins K, L and M as their Mosher esters [184]; and by chemical synthesis of both natural (-)-arteannuin M [184] and its (+)-enantiomer [186,187,188]. The structure of the natural product arteannuin O (**471**), which is epimeric with arteannuin M (**470**) at the 4-position, was confirmed by X-ray crystallography; arteannuin O (**471**) has also been obtained by a reconstructive synthesis from artemisinin (**495**) *via* dihydro-*epi*-dexoyarteannuin B (**485**) [184].

All of the five-membered lactones, dihydro-deoxyarteannuin B (**484**) [129], dihydroarteannuin B (**479**) [166,189] and dihydro-*epi*-deoxyarteannuin B (**485**) [166,189] were also fully characterized by 2D-NMR when first reported as natural products. Dihydro-*epi*-deoxyarteannuin B (**485**) has since been obtained by synthesis on several occasions [68,169,190,191,192] and is probably derived from the allylic hydroperoxide (**481**) *in vivo* as shown in Scheme 5. 4α,5α-Epoxy-6α-hydroxy amorphan-12-oic acid (**489**) can be regarded as the lactone-ring opened analogue of dihydroarteannuin B (**479**) [193]; and α-epoxyartemisinic acid (**487**) has also been obtained by synthesis [194].

**Table 36:** Amorphane Sesquiterpenoids (Decahydro-1,6-dimethyl-4-(1-methylethyl)-naphthalene, 9CI).

Name	Alternative Name(s)	CAS Number	References
4,7(11)-Amorphadien-12-al (**449**)	Cadin-4,7(11)-dien-12-al (name ascribed by original authors)	[67604-12-0]	[66]
4(15),11-Amorphadien-9-one (**450**)	Cadin-4(15),11-dien-9-one (name ascribed by original authors)	[159662-31-4]	[66]
(-)-Amorpha-4,11-diene (**451**)	Naphthalene, 1,2,3,4,4a,5,6,8a-octahydro-4,7-dimethyl-1-(1-methylethenyl)-, (1*R*,4*R*,4a*S*,8a*R*)-	[92692-39-2]	[135, 136, 196]
4-Amorphene-3,7-diol (3α,7α) (**452**)			[97]
4-Amorphene-3,7-diol (3α,7α), acetate- (**453**)	7α-Dihydroxyamorph-4-ene 3-acetate		[9]
4-Amorphen,3,11-diol (**454**)	1-Naphthalenemethanol, 1,2,3,4,4a,5,6,8a-octahydro-6-hydroxy-α,α,4,7-tetramethyl-	[159662-32-5]	[66]
4-Amorphen,3,11-diol 3-(2-methylpropanoyl) (**455**)	3-Isobutylcadin-4-en-11-ol	[159662-30-3]	[66]
Amorph-4-en-7-ol (**456**)	1-Naphthalenol, 1,2,3,4,4a,5,6,8a-octahydro-4,7-dimethyl-1-(1-methylethyl)-, (1*R*,4*R*,4a*S*,8a*R*)-	[140385-39-3]	[134]
Annulide (**457**)	Naphtho[1,8-bc]pyran-2(*3H*)-one, decahydro-6-methyl-3,9-bis(methylene)-[3a*R*-3aα,6α,6aα,9aβ,9bα)-	[103739-95-3]	[97, 128, 129, 182, 197]
*trans*-Arteannuic alcohol (**458**)	Artemisinic alcohol Amorpha-4,11-dien-12-ol 1-Naphthaleneethanol, 1,2,3,4,4a,5,6,8a-octahydro-4,7-dimethyl-β-methylene-, (1*R*,4*R*,4a*S*,8a*R*)	[125184-95-4]	[27, 32, 43, 135, 196]
*cis*-Arteannuic alcohol (**459**)	4,11(13)-Cadinadien-12-ol	[147648-62-2]	[27, 32, 43]
Artemisinic aldehyde (**460**)	1-Naphthaleneacetaldehyde, 1,2,3,4,4a,5,6,8a-octahydro-4,7-dimethyl-α-methylene-, (1*R*,4*R*,4a*S*,8a*R*)-	[125276-60-0]	[135, 196]
Arteannuin A (**461**)	Artemisinin I Qinghaosu I	[82442-48-6]	[56,171,172, 198, 199]
Arteannuin B (**462**)	Qing Hau Sau II Arteannuin C	[50906-56-4]	[15, 20, 25, 55, 59, 69 96, 105, 147,149, 153]
Arteannuin E (**463**)	Qinghaosu V 4-Hydroxy-11(13)-amorphen-12,5-olide; 4β,5α	[82003-84-7]	[56, 132, 147, 150, 176, 180, 182, 199, 205]
Arteannuin F (**464**)	Artemisilactone 4-Hydroxy-11(13)-amorphen-12,5-olide 4α,5α	[92691-97-9]	[56, 99, 132, 147, 150,165, 176, 180, 199,205]
Arteannuin H (**465**)	Naphtho[1,8-cd]-1,2-dioxepin-3(*4H*)-one, decahydro-4,7-dimethyl-10-methylene- (4*R*,4a*R*,7*R*,7a*S*,10a*S*,10b*S*)-	[207446-83-1]	[166, 183]
Arteannuin I (**466**)	Naphtho[1,8-bc]pyran-2(*3H*)-one, decahydro-3,6-dimethyl-9-methylene- (3*R*,3a*R*,6*R*,6a*S*,9a*S*,9b*S*)-	[207446-85-3]	[129, 166]
Arteannuin J (**467**)	Naphtho[1,8-bc]pyran-2(*3H*)-one, 3a,4,5,6,6a,7,9a,9b-octahydro-3,6,9-trimethyl (3*R*,3a*R*,6*R*,6a*S.*9a*S*,9b*S*)-	[207446-87-5]	[129, 166, 201]
Arteannuin K (**468**)	*2H*-Naphtho[8a,1-b]furan-2-one, 3,3a,4,5,6,6a,7,10-octahydro-10-hydroxy-3,6,9-trimethyl-, (3*R*,3a*S*,6*R*,6a*S*,10*R*,10a*S*)-	[207446-88-6]	[166, 206]
Arteannuin L (**469**)	*2H*-Naphtho[8a,1-b]furan-2-one, decahydro-10-hydroxy-3,6-dimethyl-9-methylene-, (3*R*,3a*S*,6*R*,6a*S*,10*R*,10a*S*)-	[207446-89-7]	[166, 206]
Arteannuin M (**470**)	*2H*-Naphtho[8a,1-b]furan-2-one, decahydro-9,10-dihydroxy-3,6,9-trimethyl-, (3R,3aS,6R,6aS,9R,10R,10aS)-	[207446-90-0]	[166, 186, 187]
Arteannuin N (**472**)	5-Oxo-3-amorphen-12-oic acid	[207446-92-2]	[166]
Arteannuin O (**471**)	*2H*-Naphtho[8a,1-b]furan-2-one, decahydro-9,10-dihydroxy-3,6,9-trimethyl-, (3*R*,3a*S*,6*R*,6a*S*,9*S*,10*R*,10a*S*)-	[382600-19-3]	[184]
Artemisinic acid (**473**)	Arteannuic acid 4,11(13)-Amorphadien-12-oic acid Qing Hau acid	[80286-58-4]	[15, 20, 31, 55, 56, 59 61, 65, 69, 105, 143, 147, 148, 153, 160, 163, 164, 184, 198 200, 204, 207, 208, 209, 210, 211]
Artemisinic acid, methyl ester (**474**)	Methyl artemisinate 4,11(13)-Amorphadien-12-oic acid methyl ester	[82869-24-7]	[97, 143, 166, 212]
Artemisinin B (**475**)	1-Naphthaleneacetic acid, 1,2,3,4,4a,5,8,8a-octahydro-8,8a-dihydroxy-4,7-dimethyl-α-methylene-, (1*S*,4*R*,4a*S*,8*R*,8a*R*)-	[145941-07-7]	[65]
6,7-Dehydroartemisinic acid (**476**)	4,11(13)-Amorphadien-12-oic acid 6,7-didhydro 4,6,11(13)-Cadinatrien-12-oic acid	[120193-24-0]	[160, 163, 213]
Deoxyarteannuin B (**477**)	Deoxyisoartemisinin C	[128301-55-3]	[97, 129, 163, 175, 201, 214, 215]
*epi*-Deoxyarteannuin B (**478**)	Deoxyisoartemisinin B	[84237-06-9]	[68, 96, 97, 129, 158, 160, 163, 164, 197]
Dihydroarteannuin B (**479**)	3H-Oxireno[7,8]naphtho[8a,1-b]furan-3-one, decahydro-4,7,9a-trimethyl- [1aR-(1aα,1bR,4β,4aβ,7β,7aβ,9aα)]-	[64390-16-5]	[166]
11*R*-(-)-Dihydroartemisinic acid (**480**)	4,11(13)-Amorphadien-12-oic acid (11*R*,13-dihydro)	[85031-59-0]	[20, 32, 43, 135, 166, 167, 216]
Dihydroartemisinic acid hydroperoxide (**481**)	4-Hydroxyperoxy-5-amorphen-12-oic acid, 4α, 11*R*	[85031-60-3]	[167, 170, 185]
Dihydroartemisinic alcohol (**482**)	1-Naphthaleneethanol, 1,2,3,4,4a,5,6,8a-octahydro-α,4,7-trimethyl-, (1*R*,4*R*,4a*S*,8a*S*)-	[855425-50-2]	[135]
Dihydroartemisinic aldehyde (**483**)	1-Naphthaleneacetaldehyde, 1,2,3,4,4a,5,6,8a-octahydro-α,4,7-trimethyl-, (1*R*,4*R*,4a*S*,8a*S*)-	[855425-51-3]	[135]
Dihydro-deoxyarteannuin B (**484**)	*2H*-Naphtho[8a,1-b]furan-2-one, 3,3a,4,5,6,6a,7,8-octahydro-3,6,9-trimethyl- [3*R*-(3α,3aβ,6β,6aβ,9a*R*)]-	[89956-69-4]	[129]
Dihydro-*epi*-deoxyarteannuin B (**485**)	4-Cadinen-12,6-olide (6β,10βH,12αH)	[104196-16-9]	[60, 68, 129, 166]
Dihydroxycadinanolide (**486**)			[217]
α-Epoxyartemisinic acid (**487**)	α-Epoxy-arteannuic acid		[129, 160, 194]
α-Epoxy-dihydroartemisinic (**488**)			[9]
4α,5α-Epoxy-6α-hydroxy amorphan-12-oic acid (**489**)			[9]
Isoannulide (**490**)	Naphtho[1,8-bc]pyran-2(*3H*)-one, 3a,4,5,6,6a,7,9a,9b-octahydro-6,9-dimethyl-3-methylene-, [3a*R*-(3aα,6α,6aα,9aβ,9bα)]-	[103739-94-2]	[97, 128, 129, 182, 197]
2-Naphthalenol, decahydro-1-methyl-6-methylene-4-(1-methylethenyl)- (**491**)		[159662-33-6]	[66]
Verboccidentene (**492**)	Amorpha-4,7(11)-diene	[79982-58-4]	[134]

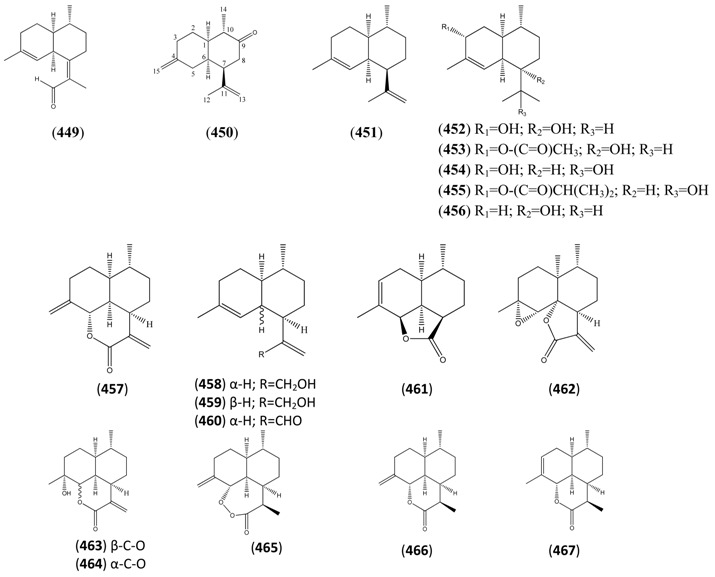
 
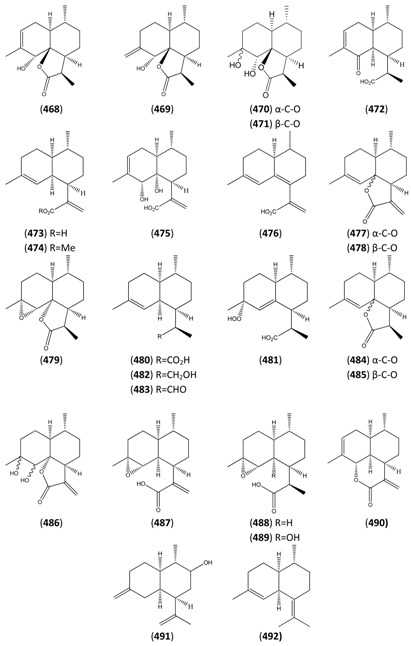

It is interesting to note that nine of the structures reported in Table 36 and Table 38 occur in both their 11,13-dihydro and 11,13-dehydro forms. These nine pairs are listed in Table 37.

**Table 37:** Amorphane and *seco*-amorphane natural products from *A. annua* which occur as both 11,13-dihydro and 11,13-dehydro forms.

11,13-Dihydro form	11,13-Dehydro form
Dihydroartemisinic acid (**480**)	Artemisinic acid (**473**)
Dihydroarteannuin B(**479**)	Arteannuin B (**462**)
Dihydro-*epi*-deoxyarteannuin B(**485**)	*epi*-Deoxyarteannuin B (**478**)
Dihydro-deoxyarteannuin B (**484**)	Deoxyarteannuin B (**477**)
α-Epoxy-dihydroartemisinic acid (**488**)	α-Epoxy-artemisinic acid (**487**)
Dihydro-*seco*-cadinane (**493**)	*seco*-Cadinane (**494**)
Arteannuin I (**466**)	Annulide (**457**)
Arteannuin J (**467**)	Isoannulide (**490**)
Artemisinin (**495**)	Artemisitene (**497**)

Feeding labelled dihydroartemisinic acid (**480**) to *A. annua* resulted in sixteen labelled amorphane and cadinane sesquiterpenes [185], which included all nine of the 11,13-dihydro forms in the “pairs” in Table 37 (*The complete list of metabolites of (**480**) is: artemisinin (**495**), dihydroarteannuin B (**479**), dihydro-*epi*-deoxyarteannuin B (**485**), arteannuin M (**470**), the *seco*-cadinane (**493**), the tertiary hydroperoxide of dihydroartemisinic acid (**481**), dihydro-deoxyarteannuin B (**484**), deoxyartemisinin (**499**), arteannuin K (**468**), arteannuin L (**469**), arteannuin H (**465**), arteannuin I (**466**), arteannuin J (**467**) and α-epoxy-dihydroartemisinic acid (**488**).

**Scheme 6:**
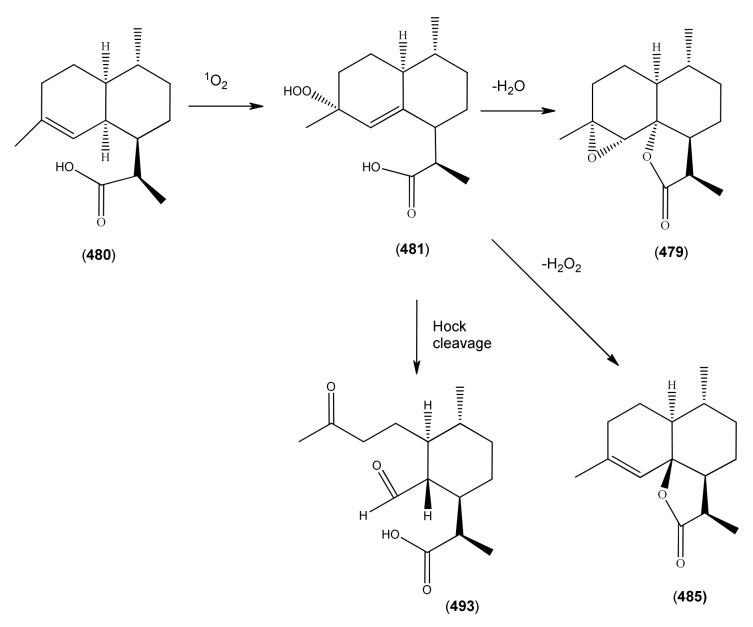
The most dominant products from metabolism of dihydroartemisinic acid (**480**) *in vivo* in *A. annua* plants.

* The most significant products from this feeding study were dihydroarteannuin B (**479**), dihydro-*epi*-deoxyarteannuin B (**485**) and the dihydro *seco*-cadinane (**493**). The pattern of transformations of dihydroartemisinic acid (**480**) which was observed *in vivo* very closely paralleled the spontaneous autoxidation chemistry for dihydroartemisinic acid which had previously been demonstrated *in vitro* [193,218]. This has lead to the proposal that the main metabolic route to all three of these metabolites and artemisinin (**495**) in *A. annua* involves the spontaneous autoxidation of dihydroartemisinic acid (**480**) and the subsequent chemical reactions of the derived tertiary allylic hydroperoxide (**481**), as is shown in Scheme 6. 

**Scheme 7:**
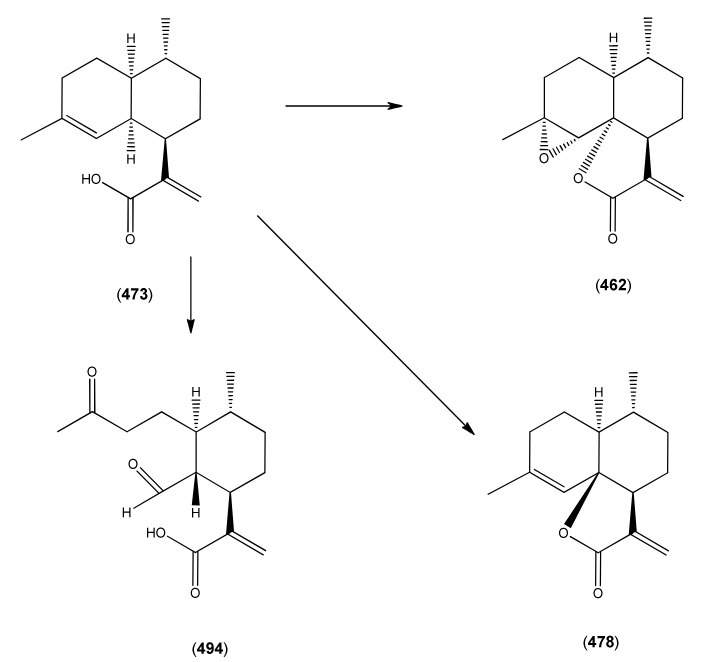
The most dominant products from metbolism of artemisinic acid (**473**) *in vivo* in *A. annua* plants.

When labeled artemisinic acid (**473**), the 11,13-dehydro analogue of dihydroartemisinic acid (**480**), was fed to intact *A. annua* plants, slightly fewer labeled metabolites were isolated [155]. However, all seven metabolites from this experiment are also known as natural products from *A. annua* and six of the seven feature in the pairs of metabolites discussed in Table 37. The most abundant metabolite from feeding artemisinic acid (**473**) was arteannuin B (**462**), followed by *epi*-deoxyarteannuin B (**478**) and the *seco*-cadinane (**494**) as shown in Scheme 7 (the remaining four metabolites are: annulide (**457**), isoannulide (**490**), deoxyarteannuin B (**477**) and artemisinic acid methyl ester (**474**), which were all isolated in trace amounts.

It is intriguing to note that there are exact structural homologies between six of the seven highly oxygenated 11,13-dehydro sesquiterpenes which have been isolated as metabolites of artemisinic acid (**473**) and a subset of the sixteen 11,13-dihydro metabolites which were obtained in the preceding study with dihydroartemisinic acid (**480**) (see Table 37). Clearly, the *in vivo* transformations of artemisinic acid (**473**) closely parallel those of its 11,13-dihydro analogue, dihydroartemisinic acid (**480**), as is shown by Scheme 5, Scheme 6 and Scheme 7. (However, note that in the case of artemisinic acid (**473**), no allylic hydroperoxide analogous to (**481**) in Scheme 6 was isolated *in vivo* as a natural product, and its existence as an intermediate in Scheme 7 must therefore be inferred. Such an allylic hydroperoxide can, however, be produced in the laboratory by chemical reactions with ^1^O_2_ and it is known to undergo *in vitro* several of the transformations which are depicted *in vivo* in Scheme 7).

It seems likely therefore that similar mechanisms are operative in the metabolism of both dihydroartemisinic acid (**480**) and artemisinic acid (**473**). These biological transformations have been proposed to involve spontaneous autoxidation of the Δ^4,5^ double bond in (**473**)/(**480**) and subsequent rearrangements of the resultant allylic hydroperoxides [155,185]. In addition, feeding experiments with both labeled precursors appear to show that artemisinic acid (**473**) and dihydroartemisinic acid (**480**) are NOT mutually interconvertible; rather, each is the committed precursor to the two large families of highly oxygenated 11,13-dehydro and 11,13-dihydro sesquiterpene metabolites which are known from this species (see, for example, the nine pairs of compounds in Table 37). This observation fits well with the reported occurrence of two chemical races of *A. annua*: a low-yielding-artemisinin chemotype, which is rich in artemisinic acid; and a high-yielding-artemisinin chemotype, which also contains significant quantities of dihydroartemisinic acid, as discussed in Section 4.1.

##### 2.6.3.3. Seco*-Cadinanes*

Artemisinin (**495**) is a *seco*-cadinane sesquiterpene, which was first isolated from *A. annua* in 1972. The unusual endoperoxide group in artemisinin has been confirmed by a variety of means [219,220,221,222,223,224,225,226] and the absolute stereochemistry of artemisinin has been established by X-ray crystallography [227]. Many total [171,228,229,230,231,232,233,234,235] and partial syntheses [236,237,238,239] of artemisinin have been reported and the various synthetic strategies have been reviewed [144]. Artemisitene (**497**), the 11,13-dehydro analogue of artemisinin, is present in *A. annua* at much lower levels than artemisinin (typically concentrations in the order of 0.01%, as compared with 1%) [240], although artemisitene can be obtained readily by chemical transformations of artemisinin [165,213,241,242]; and a partial synthesis of artemisitene from artemisinic acid (**473**) has also been reported [243]. 

Artemisinin (**495**) is believed to be localized primarily within the glandular trichomes of *A. annua*, which are loosely attached to the leaf surface [244]. Perhaps because of this highly accessible location, it has been claimed that artemisnin can be extracted in 97% yield simply by dipping a leaf in chloroform for 5 seconds! 

**Table 38:** *Seco*-Cadinane, *nor*-Cadinane and *abeo*-Cadinane Sesquiterpenoids.

Name	Alternative Name(s)	CAS Number	References
1α-Aldehyde-2β-[3-butanone]-3α-methyl-6β-[2-propanoic acid]-cyclohexane (**493**)			[9]
1α-Aldehyde-2β-[3-butanone]-3α-methyl-6β-[2-propenoic acid]-cyclohexane (**494**)	4,5-Dioxo-4,5-*seco*-11(13)cadinen-12-oic acid		[9, 217]
Artemisinin (**495**)	Arteannuin Qinghaosu Octahydro-3,6,9-trimethyl-3,12-epoxy-*12H*-pyrano[4,3-j]-1,2,benzodioxepin-10(*3H*)-one	[63968-64-9]	[4, 5, 15, 20, 34, 55, 56, 59, 69, 82, 91, 95, 96, 98, 105, 147, 150, 156, 171, 198, 200, 203, 204, 211, 213, 214, 215, 220, 230, 231, 234, 236, 239, 245, 246, 247, 248, 249, 250, 251, 252]
Arteannuin G (**496**)			[9, 56, 132, 147, 150, 176, 180, 182, 199, 205, 253]
Artemisitene (**497**)	Artemisinin, 11,13-didehydro	[101020-89-7]	[25, 55, 149, 200, 213, 250]
Arteannuin D (**498**)	3α-Hydroxy-deoxyartemisinin Qinghaosu IV Artemisinin IV	[82003-85-8]	[15, 56]
Deoxyartemisinin (**499**)	Deoxyarteannuin Qing Hau Sau III Artemisinin III Octahydro-3,6,9-trimethyl-10αH-9,10b-epoxy-pyrano[4,32-jk][2]benzoxepin-2(*3H*)-one	[72826-63-2]	[56,59,60,105,132,145,147,169,229,254]
3α-Hydroxy-4α,5α-epoxy-7-oxo-(8[7→6]-abeo-amorphane (**500**)			[9]
Norannuic acid (**501**)		[152135-59-6]	[199]
Norannuic acid formyl ester (**502**)			[9]
15-*nor*-10-Hydroxy-oplopan-4-oic acid (**503**)			[9]
1-Oxo-2β-[3-butanone]-3α-methyl-6β-[2-propanoic acid]-cyclohexane (**504**)			[9]
1-Oxo-2β-[3-butanone]-3α-methyl-6β-[2-propanol formyl ester]-cyclohexane (**505**)			[9, 218]

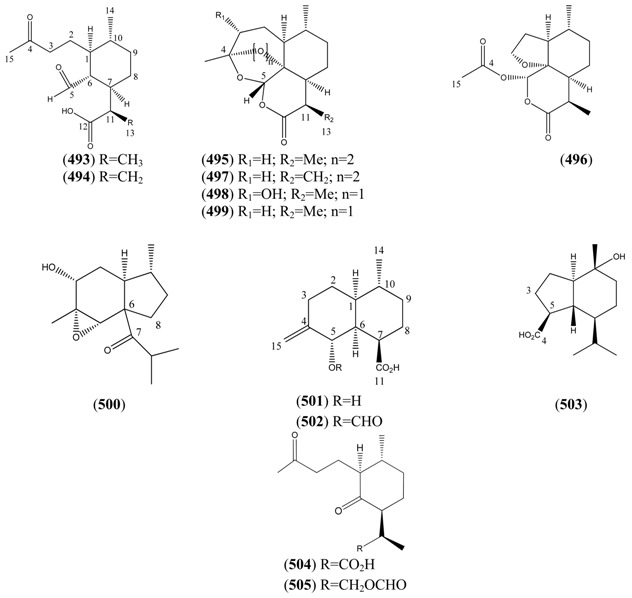

Deoxyartemisinin (**499**) is some 100-300 times less potent than artemisinin (**495**) as an antimalarial drug, which demonstrates that the peroxy linkage in artemisinin is indeed necessary for its biological activity. Several syntheses of deoxyartemisinin have been reported [171,229,236,254,255]. Fully assigned 2D-NMR data are available for artemisinin (**495**) [221], the *seco*-cadinane **493** [9] and deoxyartemisinin (**499**) [165].

The formyl ester in *nor*-amorphane **502** is thought to have been derived from the carbon at the 12-position of an amorphane precursor by oxidative rearrangements. This group has been lost altogether, presumably as the result of ester hydrolysis, in the bis-*nor*-amorphane sesquiterpene, norannuic acid **501** [66,199]. Similarly, the formyl group in 1-oxo-2β-[3-butanone]-3α-methyl-6β-[2-propanol formyl ester]-cyclohexane (**505**) [218] is probably also derived from oxidative rearrangements, this time deriving from the 5-position of an appropriate bicyclic precursor (the related *seco*-cadinane natural product **504** has also been reported from the *in vitro* autoxidation of dihydroartemisinic acid in organic solution) [193]. It seems likely that the ethyl substituent in artemisinin G (**496**) might be derived from the 4- and 15-positions of a conventional amorphane precursor. 

The novel carbon skeleton of compound **500** is thought to be derived from the amorphane skeleton by migration of C-8 from C-7 to C-6 (*i.e.*, compound **500** is an 8(7→6) *abeo* amorphane), which results in a contraction of the B ring from six atoms to five. Similarly, the unusual carbon skeleton of **503** might also have arisen from an amorphane precursor, in which the A ring has been contracted from six to five atoms, as a result of carbon-carbon bond migration of C-3 from C-4 to C-5 [9].

##### 2.6.3.4. Guaianes

Guaianes (Table 39) are bicyclic sesquiterpenes which contain fused 5- and 7-membered rings. Guaianes are often found together with eudesmanes (which contain two fused 6-membered rings – see Table 34) and may be derived by an alternative cyclization of the same germacrane precursor which gives rise to eudesmanes. 

**Table 39:** Guaiane Sesquiterpenoids (Decahydro-1,4-dimethyl-7-(1-methylethyl)azulene, 9CI).

Name	Alternative Name(s)	CAS Number	References
α-Guaiene (**506**)	1(5),11-Guaiadiene	[3691-12-1]	[28, 36]
β-Guaiene (**507**)	1(5),7(11)-Guaiadiene	[88-84-6]	[32]
γ-Gurjunene (**508**)	5,11-Guaiadiene	[22567-17-5]	[31, 34]
Guaiazulene (**509**)	2,4-Dimethyl-7-(1-methylethyl)azulene	[492-45-5]	[24]

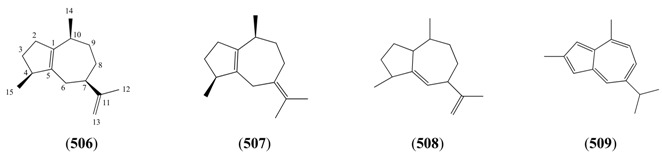

Aromadendrane sesquiterpenes (Table 40) are 6,11-cycloguaianes, in which an additional cyclopropyl ring has been formed by further cyclization of a guaiane precursor. The unusual metabolite, nortaylorione (15-*nor*-1,10-*seco*-1(5)-aromadendrene-4,10-dione [163128-16-3]) (**519**) [257], might be viewed as the product of oxidative rearrangements of such an aromadendrane precursor. The cubebane sesquiterpenoids (octahydro-3,7-dimethyl-4-(1-methylethyl)-*1H*-cyclo-penta[1,3]-cyclopropa-[1,2]-benzene, 9CI): α-cubebene (3-cubebene [17699-14-8]) (**520**) [51] and β-cubebene [4(15)-cubebene [13744-15-5], (**521**)] [19,38,51,66], are also representatives of guaianes which have undergone a further cyclization, with the cyclopropyl ring now being formed between positions 1- and 6-. α-Guaiane (**506**) is the most abundant guaiane sesquiterpene in *A. annua* [22], whilst spathulenol (**515**) is the most common aromadendrane [41] (both can reach levels up to 5% of the essential oil).

**Table 40:** Aromadendrane Sesquiterpenoids (Decahydro-1,1,4,7-tetramethyl-*1H*-cycloprop[e]azulene. 9CI).

Name	Alternative Name(s)	CAS Number	References
α-Aromadendrene (**510**)	*allo*-Aromadendrene 10(14)-Aromadendrene	[25246-27-9] [14682-34-9]	[23, 40, 43]
α-Gurjunene (**511**)	4-Aromadendrene	[489-40-7]	[48]
Globulol (**512**)	(1α,4α,5β,6α,7α,10α)-10-Aromadendranol	[489-41-8]	[19, 28, 34]
*epi*-Globulol (**513**)	*1H*-Cycloprop[e]azulen-4-ol, decahydro-1,1,4,7-tetramethyl-, (1a*R*,4*S*,4a*R*,7*R*,7a*S*,7b*S*)-	[88728-58-9]	[34]
Ledol (**514**)	10-Aromadendrol (1β,4α,5β,6β,7β,10α)	[577-27-5]	[28]
(-)-Spathulenol (**515**)	10(14)-Aromadendren-4-ol	[77171-55-2] [6750-60-3]	[19, 27, 31, 32, 41, 43, 45]
Cycloprop[7,8]azuleno[3a,4-b]oxirene, decahydro-1,4a,7,7-tetramethyl-, (1*R*,6a*R*,7a*R*,7b*S*)- (**516**)		[199983-75-0]	[34]
Aromadendrene epoxide (**517**)	Isoaromadendrene epoxide 10(14)-Aromadendrene 10β,14-epoxide	[85710-39-0] [499134-59-7]	[23, 34]
Cyclocolorenone (**518**)	4-Aromadendren-3-one	[489-45-2]	[45]

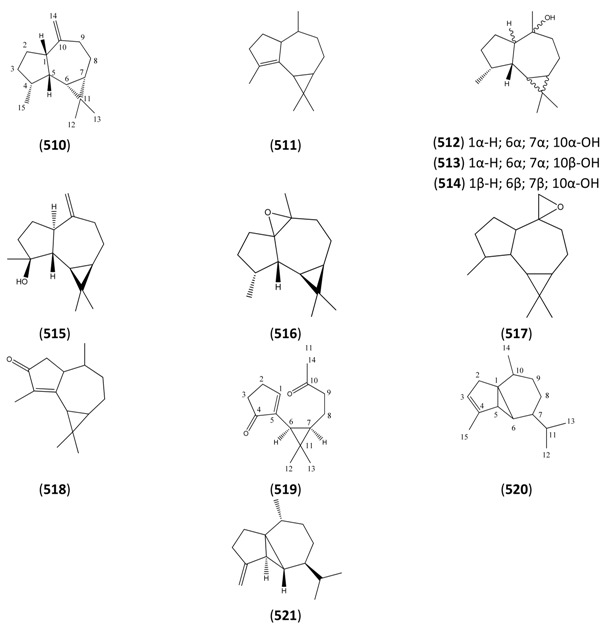

#### 2.6.4. Tricyclic Sesquiterpenes

Several tricyclic sesquiterpenes are known from *A. annua*, although none have been reported in very significant quantities*.* These include α-longipinene (**522**) (3-longipinene [5989-08-2]) [22,66], β-longipinene (**523**) ([41432-70-6]) [27] and *trans*-longipinocarveol (**524**) ([889109-69-7]) [23], which are representatives of the longipinane (2,6,6,9-tetramethyltricyclo[5.4.4.0 ^2,8^]undecane, 9CI) skeleton. Longifolene (junipene [475-20-7]) (**525**) [28] is a longifolane sesquiterpenoid (decahydro-4,8,8,9-tetramethyl-1,4-methanoazulene, 9CI).

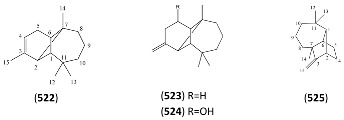



The acorane (1,8-dimethyl-4-(1-methylethyl)spiro[4.5]decane, 9CI) and chamigrane (11,5,9-tetramethylspiro[5,5]undecane, 9CI) sesquiterpenoids: β-acorenol (3-acoren-11-ol [28400-11-5]) (**526**) [19] and β-chamigrene (2,7(14)-chamigradiene [18431-82-8]) (**527**) [19] are also included here.

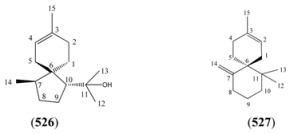



The largest group of tricyclic sesquiterpenes to be described rom *A. annua* is the cedranes (Table 41). A cDNA clone encoding *epi*-cedrol synthase has been shown to catalyze the formation of the oxygenated sesquiterpene *ep*i-cedrol from FPP (**378**) [258,259]. Trace amounts of the sesquiterpene hydrocarbons α-cedrene, β-cedrene, (E)-β-farnesene, α-acoradiene and (*E*)-β-bisabolene were also produced by the operation of this enzyme [258]. The structure of 3α,15-dihydroxycedrane (**534**) from *A. annua* has been rigorously determined both by 2D-NMR and by synthesis from (+)-β-cedrene [9].

**Table 41:** Cedrane Sesquiterpenoids (Octahydro-3,6,8,8-tetramethyl-*1H*-3α-7-methanoazulene).

Name	Alternative Name(s)	CAS Number	References
Cedrol (**528**)	3-Cedranol Cedran-8-ol 6-Isocedrol *epi*-Cedrol	[77-53-2] [19903-73-2]	[19, 27, 32, 43, 45, 48]
Cedryl acetate (**529**)	3-Cedranol acetate	[77-54-3]	[27]
Cedra-8(15)-en-9α-ol (**530**)	β-Cedren-9-ol Cedrenol	[13567-41-4] [28231-03-0]	[27, 43]
Cedra-8(15)-en-9α-ol acetate (**531**)		[65082-66-8]	[27, 32, 43]
3-Cedren-12-ol (**532**)		[18319-35-2]	[27]
Cedra-8-en-13-ol, acetate (**533**)		[18319-34-1]	[27]
3α,15-Dihydroxy cedrane (**534**)			[9]

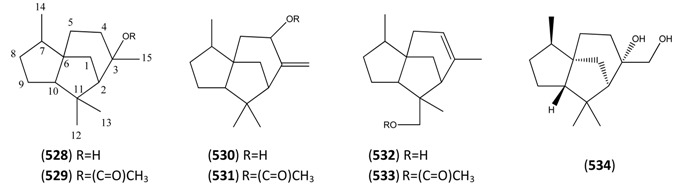

Other tricyclic sesquiterpenes from *A. annua* belong to the silphinane (decahydro-1,4,4,5a-tetra-methylcyclopenta[c]-pentalene); isocomane (dehydro-1,3α,4,5-tetramethylcyclopenta[c]pentalene); α-santalane (2,3-dimethyl-2-(4-methyl-pentyl)tricycle[2.2.1.0^2,6^]-heptane); copaane (1,3-dimethyl-8-(1-methylethyl)tricyclo[4.4.4.4^2,7^]decane, 9CI); bourbonane (decahydro-3a,6-dimethyl-1-(1-methylethyl)-cyclobuta[1,3:3,4]-dicyclopentene, 9CI); and α-*trans*-bergamotol classes of sesquiterpenoid. These include: silphinene (1-silphinene [74284-57-4]) (**535**) [27], α-isocomene (isocomene [65372-78-3]) (**536**) [27], β-isocomene (3(13)-isocomene [74311-15-2]) (**537**) [27], (*Z*)-α-santalol (α-santal-10-en-12-ol [115-71-9]) (**538**) [19], α-copaene (3-copaene [3856-25-5]) (**539**) [19, 22, 23, 24, 31, 43, 45, 48, 50, 66], β-copaen-4α-ol (tricyclo[4.4.0.0^2,7^]decan-4-ol, 1-methyl-3-methylene-8-(1-methylethyl)-, (1*R*,2*R*,4*S*,6*S*,7*S*,8*S*)- [124753-76-0]) (**540**) [43], α-ylangene ([14912-44-8]) (**541**) [25,30,40,113], β-bourbonene (4(15)-bourbonene [5208-59-3]) (**542**) [45], α-bergamentol [88034-74-6]) (**543**) [27, 32, 43], *trans*-α-bergamotyl acetate ([87978-33-4]) (**544**) [27, 43], *trans*-α-bergamotyl acetic anhydride ([960148-87-2]) (**545**) [32], α-neoclovene ([4545-68-0]) (**546**) [41,50], (-)-neoclovene-(II) [56684-96-9] (**547**) [46], neoisolongifolene [79982-57-3] (**548)** [48] and 4,4,8-trimethyltricyclo[6.3.1.0^(1,5)^]-dodecane-2,9-diol ([372968-04-2]) (**549**) [20].

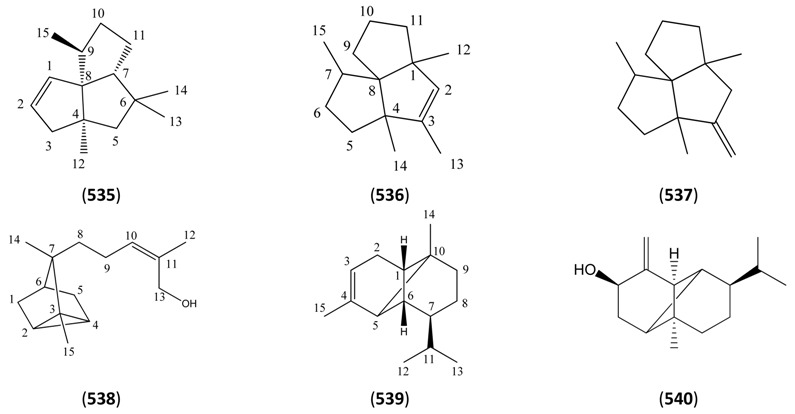

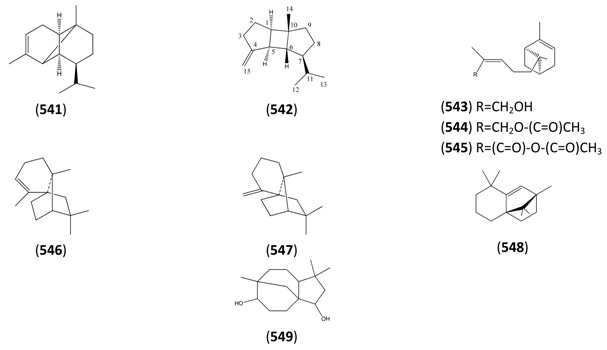





### 2.7. Higher Terpenoids

#### 2.7.1. Diterpenes

**Scheme 8:**
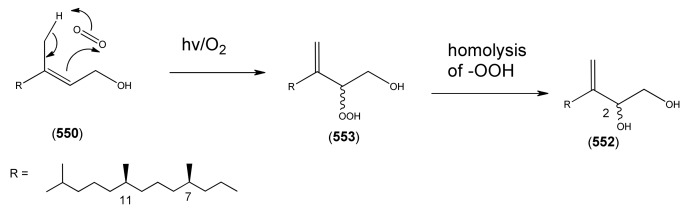
Proposed formation of phytene-1-ol-2-hydroperoxide (**553**) by spontaneous autoxidation of phytol (**550**) and subsequent homolysis/reduction of **553** to phytene-1,2-diol (**552**).

The structure of the diterpene phytene-1-ol-2-hydroperoxide (**553**) [9,260] was wrongly assigned as phytene-1,2-diol (**552**), when it was first described as a natural product from *A. annua* [261]. Structural revision was made on the basis of studies of the photo-oxygenation of commercially-available phytol, which produced both phytene-1,2-diol (**552**) (in racemic form), as well as its 2-hydroperoxy analogue (**553**) [260]. Both phytol (**550**) itself and an authentic sample of phytene-1,2-diol (**552**) were subsequently obtained as natural products from the seeds of *A. annua* [9]. Natural phytol is expected to have the 7*R,*11*R* absolute configuration [16], and natural phytene-1,2-diol (**552**) was therefore assigned as being a mixture of epimers at the 2-position on the basis of its NMR spectra and on the assumption that the configuration at the 7- and 11- positions of (**552**) remained fixed, as in phytol. The observance of epimers is most easily explained if both the hydroperoxide (**553**) and alcohol (**552**) are products of the spontaneous autoxidation of phytol (**550**), occurring within the tissues of *A. annua* plants as is shown in Scheme 8. 

**Table 42:** Phytane Diterpenoids (2,6,10,14-Tetramethylhexadecane).

Name	Alternative Name(s)	CAS Number	References
Phytol (**550**)	2-Phyten-1-ol (2*E*, 7*R*,11*R*)	[150-86-7]	[27, 31, 43, 45]
Isophytol (**551**)	1-Phyten-3-ol 3,7,11,15-Tetramethyl-1-hexadecen-3-ol	[505-32-8]	[27]
Phytene-1,2-diol (**552**)	3(20)-Phytene-1,2-diol (7*R*,*11*R)		[9, 260, 261]
Phytene-1-ol-2-hydroperoxide (**553**)			[9, 260, 261]
(2*E*)-Hexadecene (**554**)	3,7,11,15-Tetramethylhexadec-2-ene	[2437-936] [532426-78-1]	[20]
Phytone (**555**)	Hexahydrofarnesyl acetone 6,10,14-Trimethyl-2-pentadecanone	[502-69-2]	[23]

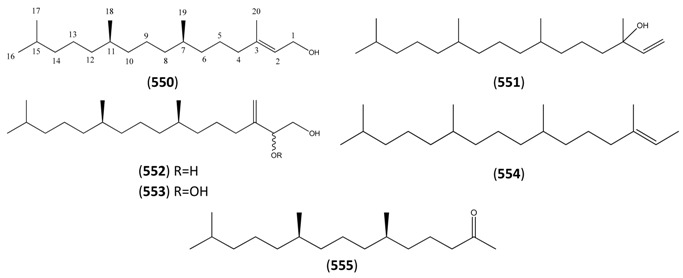

A handful of cyclic diterpenes are also known from *A. annua*, including 13-*epi*-manool (8(17),14-labdadien-13-ol [1438-62-6]) (**556)** [43], 8(14),15-isopimaradiene ([1686-56-2]) (**557**) [32] and abscisic acid ([21293-29-8]) (**558**) [262].

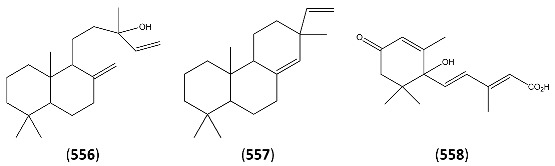



#### 2.7.2. Triterpenes and Sterols

The most abundant sterols from *A. annua* are stigmasterol (**570**) and sitosterol (**568**) [96], which are ubiquitous components of plant cell membranes. Squalene synthase (SQS) is the enzyme which catalyses the first committed step in the pathway leading from FPP (**378**) to triterpenes and phytosterols, such as these (Table 43). The SQS gene and cDNA have been successfully cloned and sequenced from *A. annua* on several occasions [263,264,265]. Much of the interest in SQS stems from its position at a key point in terpenoid biosynthesis, in which FPP (**378**) branches either to triterpenes or sesquiterpenes. Thus, it is possible that suppression of SQS expression [267] could be used to enhance the biosynthesis of artemisinin (**495**), which is a product of the alternative sesquiterpene pathway from FPP (**378**) [268].

A β-amyrin synthase, responsible for cyclization of squalene to the tritepene skeleton, has also been obtained from *A. annua*. It was possible to produce the triterpene β-amyrin (**559**) in significant amounts when this enzyme was engineered into *Saccharomyces cerevisiae* (two other enzymes in the pathway: 3-hydroxy-3-methylglutaryl-CoA reductase and lanosterol synthase were also manipulated in these experiments) [269].

**Table 43:** Triterpenoids and Sterols.

Name	Alternative Name(s)	CAS Number	References
*Oleananes*			
β-Amyrin (**559**)	12-Oleanen-3-ol	[559-70-6]	[58, 63, 66]
β-Amyrin 3-acetate (**560**)	12-Oleanen-3-ol acetate	[1616-93-9]	[58, 66, 198]
Oleanolic acid (**561**)	3β-Hydroxy-12-oleanen-28-oic acid	[508-02-1]	[58, 66, 96]
*Friedalanes*			
Friedelan-3-β-ol (**562**)	Epifriedelanol	[5085-72-3] [16844-71-6]	[105]
Friedelin (**563**)		[559-74-0]	[105]
*Ursanes*			
α-Amyrin (**564**)	12-Ursen-3-ol	[638-95-9]	[58, 63, 66, 198]
α-Amyrenone (**565**)	α-Amyrone 12-Urs-en-3-one	[638-96-0]	[58, 66]
*Taraxastanes*			
Taraxasterone (**566**)	20(30)-Taraxasten-3-one	[6786-16-9]	[58, 66]
Baurenol (**567**)	7-Bauren-3-ol	[6466-49-0]	[58, 66]
*Sterols*			
β-Sitosterol (**568**)	Stigmast-5-en-3-ol	[83-46-5]	[15, 59, 61, 63, 69, 75, 78, 96, 198]
Daucosterol (**569**)	Stigmast-5-en-3-ol *O*-beta-D-glucopyranoside	[474-58-8]	[15]
Stigmasterol (**570**)	Stigmast-5,22-dien-3-ol 3β (22*E*,24*S*)	[83-48-7]	[56, 59, 61, 63, 65, 69, 75, 78, 96, 105, 198]

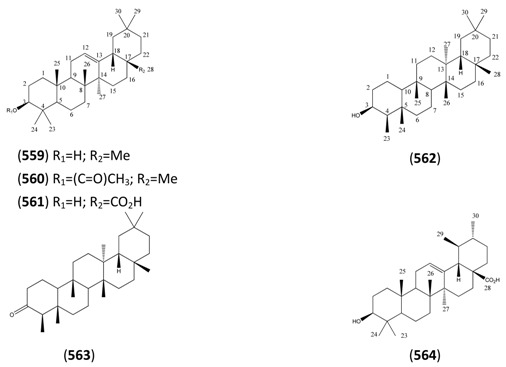
 
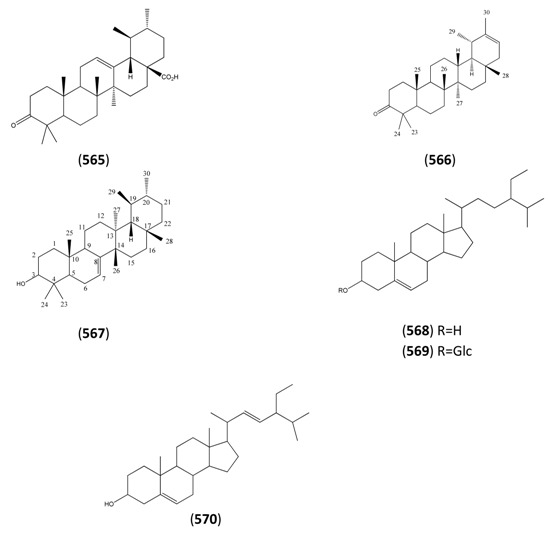

### 2.8. Nitrogen-Containing Natural Products

Only a small number of peptides and other nitrogen-containing natural products are reported from *A. annua*. These include: aurantinamide acetate ([97558-66-2]) (**571**)] [75], 6-amino-7,8-dihydro-2-hydroxypurine (7,8-dihydro-2-hydroxyadenine) (**572**) [270] and benzothiazole ([95-16-9]) (**573**) [24]. 

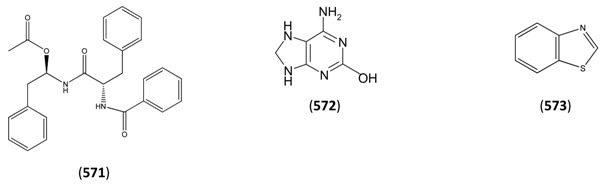



## 3. The Biosynthesis of Artemisinin (Qinghaosu)

**Scheme 9:**
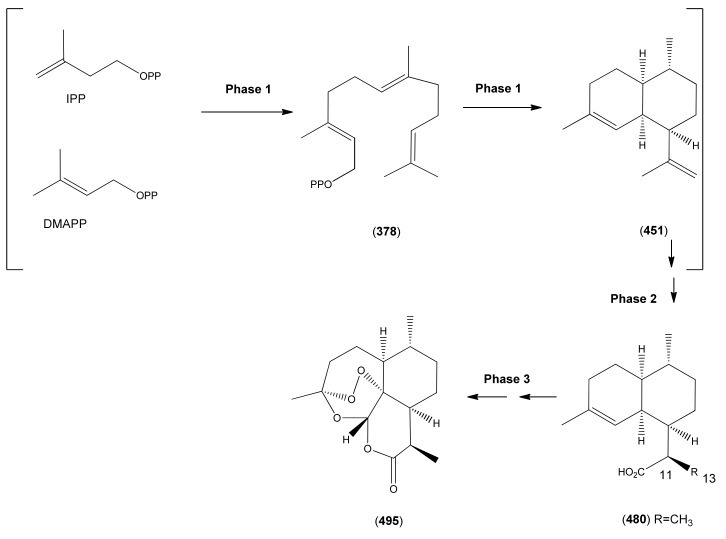
Three phases in the biosynthesis of artemisinin (**495**).

Terpene biosynthesis [271,272,273,274,275,276] and its regulation [277,278] in *A. annua* have been well reviewed, including the central role of amorpha-4,11-diene (**451**) in the biosynthesis of artemisinin (**495**) [279] and the likelihood that glandular trichomes are the location wherein artemisinin biosynthesis actually occurs [244,280] (indeed, it has recently been concluded that artemisinin biosynthesis occurs in the two outer apical cells of the glandular secretory trichomes [104] – the glandular trichomes of *A. annua* are comprised of 10 cells in total). The biosynthesis of artemisinin will be considered in three phases, as depicted in Scheme 9.

It should be emphasised that this view of the biosynthetic route to artemisinin is still not universally accepted. Although there is increasing experimental evidence in support of its general correctness, there are a significant number of experimental results (particularly in the “older” literature) which might appear to contradict Scheme 9. Thus, for many years, it was assumed that artemisinic acid (R=CH_2_ for **473** in place of R=CH_3_ for **480** in Scheme 9) [148,215,251,281,282,283,284], rather than dihydroartemisinic acid (**480**; R=CH_3_), was the late-stage precursor to artemisinin at the juncture between phases 2 and 3.

A large number of other cadinane and amorphane sesquiterpenes, including: arteannuin B (**462**) [248,285]; *epi*-deoxyarteannuin B (**478**) [286]; dihydroarteannuin B (**479**) [249]; dihydro-*epi*-deoxyarteannuin B (**485**) [286]; the *seco*-cadinane (**494**) [217]; and artemisitene (**497**) [211,248,284] have also been suggested as late-stage intermediates at or around this point in the biosynthesis (in addition, α-epoxyartemisinic acid (**487**) has been stated not to be a biosynthetic intermediate to artemisinin [194]). The experimental evidence for each of these precursors is discussed individually in some detail in Section 3.3 (phase 3 of the biosynthesis of artemisinin). However, as the length of the foregoing list shows, there have been so many different proposals for phase 3 of the biosynthesis, that not all of them can be correct. In fact, none of **462**, **478**, **479**, **485**, **494** or **497** feature in the most likely biogenetic route to artemisinin (**495**) which is discussed first in Section 3.3, although many of these compounds have been implicated as side-products, in reactions which diverge away from this main biosynthetic route to (**495**) (see Scheme 5, Scheme 6 and Scheme 7 in Section 2.6.3 for example).

### 3.1. Phase 1 (Isopentenyl Pyrophosphte to Amorpha-4,11-diene)

This first phase in the biosynthesis of artemisinin is the least controversial, and most of the enzymes involved in the conversion of isopentenyl pyrophosphate (IPP) and its isomer dimethylallyl pyrophosphate (DMAPP) to amorpha-4,11-diene (**451**) have now been isolated and characterized from *A. annua*. It has been proposed that the IPP used in the biosynthesis of artemisinin comes from both the mevalonate and the mevalonate-independent pathways [287], and it has recently been demonstrated that the cental isoprenoid unit in the FPP (**378**) precursor to artemisinin is predominantly biosynthesized from the non-mevalonate pathway [102]. ^14^C-Labelling studies have confirmed the C_5_ terpenoid precursor IPP as a starting point for the biosynthetic pathway to artemisinin [288] and ^13^C labeling studies have also been used to characterize the photosynthetic mechanism of *A. annua* as C3 [289].

The first step in phase 1 involves the conversion of IPP and DMAPP (both C_5_) to the C_15_ intermediate, farnesyl pyrophosphate (FPP; **378**) by the enzyme farnesyl diphosphate synthase (FPPS). A cDNA encoding this enzyme, which catalyses the “head-to-tail” chain extension of a DMAPP starter by two molecules of IPP, has now been cloned from *A. annua* [290]. FPP sits at a branch point in terpenoid metabolism. Further elaboration to triterpenes and plant sterols (Table 43) requires the “head-to-head” coupling of two molecules of FPP, which is catalysed by the enzyme squalene synthase (SQS), as discussed in Section 2.7.2. Conversion of FPP (**378**) to sesquiterpenes, on the other hand, requires the operation of ionase and cyclase enzymes, such as: (*E*)-β-farnesene synthase (Section 2.6.1); germacrene A synthase (Section 2.6.2); β-caryophyllene synthase (Secton 2.6.2); and *epi*-cedrol synthase (Section 2.6.4). The comitted intermediate in the biosynthesis of artemisinin (**495**) is the bicyclic sesquiterpene-amorpha-4,11-diene (**451**) [291], which is formed from FPP (**378**) by the action of the sesquiterpene cyclase, amorpha-4,11-diene synthase (ADS) [138,292].

**Scheme 10:**
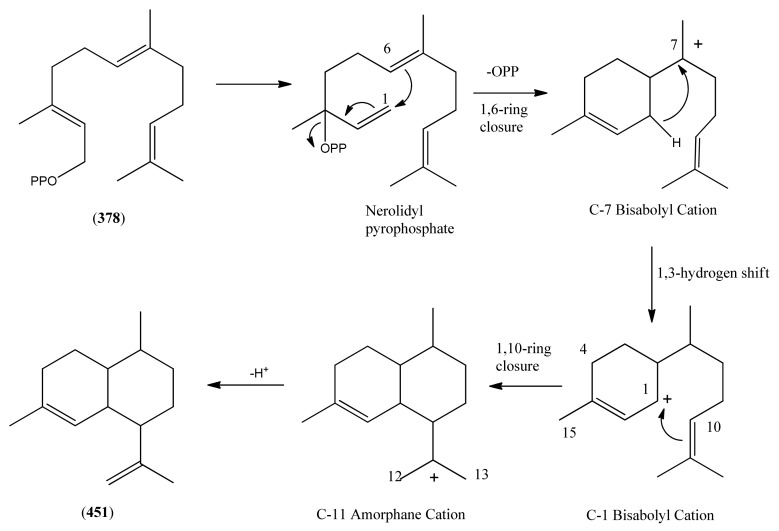
Cyclization of FPP (**378**) to amorpha-4,11-diene (**451**), catalysed by the enzyme ADS.

cDNAs encoding ADS from *A. annua* have been isolated, sequenced, and expressed [293,294]. The mechanism of this particular enzymatic cyclization of FPP has now been studied in detail using a recombinant amorpha-4,11-diene synthase from *A. annua*. It is proposed that FPP is first isomerized to nerolidyl diphosphate; ionization of nerolidyl pyrophosphate is followed by C-1,C-6-ring closure to generate a bisabolyl cation; next, this cation undergoes a 1,3-hydride shift [295] permitting a second ring closure between the 1- and 10-positions to generate the amorphane skeleton; and, finally, deprotonation at either C-12 or C-13 affords amorpha-4,11-diene (**451**) [136], as is shown in Scheme 10. This mechanism is supported by GC-MS analysis of several minor sesquiterpene products which are also produced by ADS. These include the known metabolites (*E*)-β-farnesene (**382**), α-bisabolol (**385**), amorph-4-en-7-ol (**456**) and amorpha-4-7,(11)-diene (**492**). Other minor products from ADS have not yet been reported as natural products from *A. annua*, including the amorphane, amorph-4-en-11-ol (**574**); the bisabolanes: β-sesquiphellandrene (1,3(15),10-bisabolatriene [20307-83-9]) (**575**) [134], zingiberene (1,3,10-bisabolatriene[495-60-3]) (**576**) and zingiberenol (1,10-bisaboladien-3-ol [58334-55-7] [495-60-3]) (**577**); and the humulane: γ-humulene (1,3(15),6-humulatriene [26259-79-0]) (**578**) (Figure 3) [296].

### 3.2. Phase 2 (Amorpha-4,11-diene to Dihydroartemisinic Acid)

**Scheme 11:**
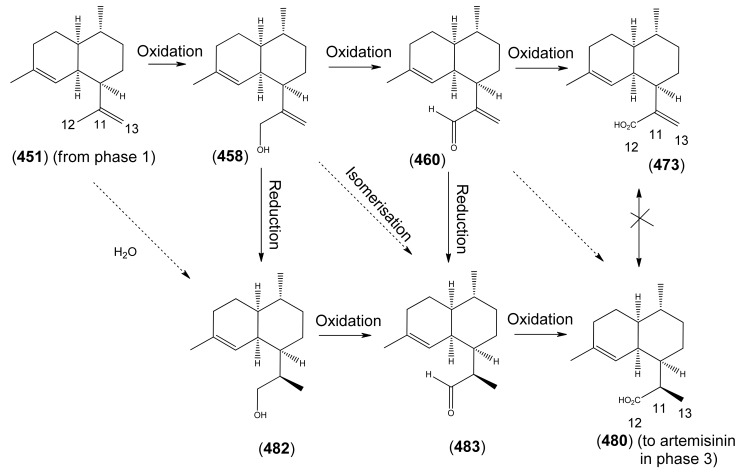
Various possible routes for the oxidation of the isopropylidene group in amorpha-4,11-diene (**451**), yielding artemisinic acid (**473**) and/or dihydroartemisinic acid (**480**) in phase 2 of the biosynthesis of artemisinin.

The second phase in the biosynthesis of artemisinin (**495**) involves modification to the isopropylidene group (C-11, C-12 and C-13) in amorpha-4,11-diene (**451**), yielding putative biosynthetic intermediates such as dihydroartemisinic acid (**480**) and artemisinic acid (**473**). Phase 2 of the biosynthesis has only recently been the subject of experimental investigation and is therefore less well understood than phase 1. In 2005, artemisinic alcohol (**458**), artemisinic aldehyde (**460**), dihydroartemisinic alcohol (**482**) and dihydroartemisinic aldehyde (**483**) were all isolated as natural products from *A. annua* [135]. Collectively, these metabolites suggested the presence of two interlinked pathways for the conversion of amorpha-4,11-diene (**451**) to either dihydroartemisinic acid (**480**) or artemisinic acid (**473**), as is shown in Scheme 11 [135].

Although it has been assumed that all transformations at the 11-, 12- and 13- positions of (**451**) in phase 2 would proceed either by oxidation reactions (horizontal) or by reduction reactions (vertical), it is also possible to postulate more “economical” routes, such as those involving isomerases, which the author has depicted by diagonal dashed arrows in Scheme 11. Thus, isomerization of the Δ^11,13^ bond to the Δ^11,12^ position in (**458**) would produce an enol, which would spontaneously tautomerize to the carbonyl group in (**483**), without any necessity for either reduction at C-11/13 or oxidation at C-12. A trichome-specific cDNA for the cytochrome P_450_ enzyme, CYP71AV1, which catalyzes three successive oxidations at the 12-position of amorpha-4,11-diene (**451**) in the “upper” oxidative pathway in Scheme 11, has now been isolated (see also Section 4.4). These three sequential oxidations of amorpha-4,11-diene (**451**) produce artemisinic acid (**473**) via artemisinic alcohol (**458**) and artemisinic aldehyde (**460**) [196] (note that the exocyclic 11,13-double bond remains intact in this route).

Historically, it has often been assumed that artemisinic acid (**473**) is then the starting point for the third and final phase of the biosynthesis of artemisinin. However, the most recent evidence suggests that dihydroartemisinic acid (**480**), produced from the saturated “lower” branch in Scheme 11, is, in fact, the true precursor to artemisinin. Kim and Kim have reported that artemisinic acid is not converted to dihydroartemisinic acid in *A. annua* [297] and more recent biosynthetic studies, employing both labeled artemisinic acid [155] and dihydroartemisinic acid [185], have confirmed that there is no interconversion in either direction between dihydroartemisinic acid (**473**) and artemisinic acid (**480**), as is represented by the “crossed” double-headed arrow in Scheme 11.

Therefore, if dihydroartemisinic acid (**480**) is the true precursor to artemisinin (**495**) in phase 3 of the biosynthesis, then reduction of the exocyclic double bond in amorpha-4,11-diene (**451**) must be occurring before artemisinic acid (**473**) in Scheme 11. Interestingly, there is now some evidence for such a route to dihydroartemisinic acid (**480**), which involves two oxidations at C-12 of amorpha-4,11-diene (**451**), producing artemisinic aldehyde (**460**) *via* artemisinic alcohol (**458**); followed by reduction of the Δ^11,13^ double bond in (**460**) to dihydroartemisinic aldehyde (**483**); and, finally, oxidation of (**483**) at C-12 to yield dihydroartemisinic acid (**480**) [135]. A recombinant DBR2 enzyme has been purified to approximately 90% from *E. coli* and found to be capable of reducing the Δ^11(13)^ double bond in artemisinic aldehyde (**460**) [but not in artemisinic alcohol (**458**), artemisinic acid (**473**), arteannuin B (**462**) or artemisitene (**497**)] [298]. This enzyme appears to be a member of the enoate reductase family of enzymes, with similarities to 12-oxophytodienoate reductases [298], and its discovery is potentially very significant with regard to defining the biosynthetic route from (**451**) to (**480**) in Scheme 11.

### 3.3. Phase 3 (Dihydroartemisinic acid to Artemisinin)

#### 3.3.1. Dihydroartemisinic Acid as a Late-Stage Precursor to Artemisinin

In this review, it is assumed that the final steps in the biosynthetic pathway to artemisinin (**495**) proceed from dihydroartemisinic acid (**480**), rather than artemisinic acid (**473**) (or, indeed, any of the other late stage intermediates: **462**, **478**, **479**, **485**, **494** or **497** which have been proposed in the past). Discussions of phase 3 of the biosynthesis therefore commence with dihydroartemisinic acid (**480**) - although the other possibilities are also evaluated in some detail in parts b) - e) of this section. Artemisinin is a *seco*-cadinane (Table 38), and carbon-carbon cleavage at C-4/C-5 in (**495**) therefore accompanies formation of the 1,2,4-trioxane ring in this final phase of the biosynthesis. No enzymes have yet been described for any of these putative reactions in phase 3. Indeed, experiments with classical plant peroxidases, a class of enzyme with the potential for involvement in these kinds of reactions, have failed to increase the yield of artemisinin [299,300]. 

It is also possible that the final transformations of dihydroartemisinic acid to artemisinin might proceed via non-enzymatic processes. In this regard, it is interesting to note that artemisinin biosynthesis has recently been correlated with increased levels of singlet oxygen -although this has been explained in terms of the upregulation of genes involved in artemisinin biosynthesis, rather than the operation of non-enzymatic processes [256]. A non-enzymatic mechanism involving molecular oxygen is particularly attractive in view of the variety of spontaneous autoxidation reactions which have already been suggested for the biogenesis of many other highly oxygenated terpenes from *A. annua.* For example, the formation of regular acyclic monoterpenes **263**-**265**, **267**, **271** and **272** in Scheme 1 (Section 2.5.1); irregular acyclic monoterpenes **279** and **280** in Scheme 3 (Section 2.5.2); eudesmane sesquiterpenes **413** and **414** in Scheme 4 (Section 2.6.3); amorphane sesquiterpenes **457**, **465**-**467**, **477**, **478**, **481**, **484**, **485** and **490** in Scheme 5; **479**, **481**, **485** and **493** in Scheme 6; **462**, **478** and **494** in Scheme 7 (Section 2.6.3); and diterpenes **552** and **553** in Scheme 8 (Section 2.7.1) have all been proposed to proceed by spontaneous autoxidation chemistry.

**Scheme 12:**
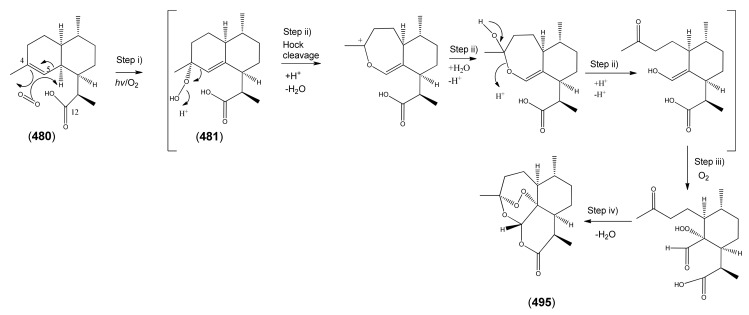
A four-step mechanism for the spontaneous autoxidation of dihydroartemisinic acid (**480**) to artemisinin (**495**) in *A. annua.*

The proposal that the final transformations to artemisinin (**495**) in phase 3 may be non-enzymatic also receives strong support both from *in vivo* studies [168,185] and from experiments which have been performed *in vitro* [167,193,218], under conditions that are relevant to the living plant. Together, *in viv*o and *in vitro* experiments have suggested a mechanism for the conversion of dihydroartemisinic acid (**480**) to artemisinin (**495**) *via* a spontaneous autoxidation process, involving four steps, as is shown in Scheme 12.

The four steps shown in Scheme 12 are: i) photo-sensitized reaction of the Δ^4,5^-double bond in dihydroartemisinic acid (**480**) with singlet molecular oxygen (*via* an “ene-type” mechanism); ii) Hock cleavage of the resulting tertiary allylic hydroperoxide **481**; iii) oxygenation of the enol product from Hock cleavage; and iv) cyclization of the resulting vicinal hydroperoxyl-aldehyde to the 1,2,4-trioxane system of artemisinin (**495**). The tertiary allylic hydroperoxide **481**, which is produced in step i), has already been shown to be a biosynthetic intermediate, linking dihydroartemisinic acid (**480**) and artemisinin (**495**), by *in vivo* experiments with labeled dihydroartemisinic acid (**480**) [185,189]. Compound **481** has also been described independently as a natural product from *A. annua* [167,170]; and quantitative studies, which monitored the decline of dihydroartemisinic acid (**480**) and the increase of artemisinin (**495**) during leaf development and senescence have confirmed that an intermediate, such as **481**, is probably involved in this conversion [301] [it is interesting to note that the alternative secondary allylic hydroperoxide from the spontaneous autoxidation of dihydroartemisinic acid (**480**) in step i] has also been proposed as the precursor to another amorphane sesquiterpene endoperoxide from *A. annua*, arteannuin H (**465**) [166], as is shown in Scheme 5 in Section 2.6.3)*.*

There is – as yet – no direct evidence from *in vivo* studies to suggest how further transformation of **481** to the 1,2,4-trioxane ring in artemisinin might occur in *A. annua*. The evidence for steps ii)-iv) which are proposed in Scheme 12 comes solely from *in vitro* studies, conducted with the tertiary allylic hydroperoxide **481** [171,236,237,239,254], which can be obtained in good yield from photosensitized oxygenation of **480** [193,218]. These *in vitro* experiments have shown that **481** can indeed undergo sponataneous conversion to a transient enol as proposed in step ii) of Scheme 11 [204,238,243,302,303], and it has even been possible to fully characterize this unstable intermediate by 2D-NMR at low temperature [193]. This known reaction of allylic hydroperoxides is referred to as a Hock cleavage (or a Criegee rearrangement) and it is in this step that carbon-carbon bond cleavage occurs between C-4 and C-5, thereby producing the *seco-*cadinane skeleton of artemisinin (**495**). 

The double bond in the enol which is produced by step ii) then reacts rapidly with a second molecule of oxygen in step iii). This reaction most probably proceeds *via* an “ene-type” mechanism similar to that for the conversion of **480** to **481**, although apparently without the requirement for light (*i.e.* ^3^O_2_ rather than ^1^O_2_ is perhaps the active species) [193,236,304,305]. All carbons in the resultant α-aldehydo hydroperoxide [238] are now at the correct oxidation level and this intermediate immediately “zips up” to the 1,2,4-trioxane ring of artemisinin (**495**) in step iv). 

It has been speculated that the proximity of the 12-carboxylic acid group to the Δ^4,5^-double bond in **480** and the hydroperoxide functionality in **481** might be the reason why both transformations i) and ii) proceed with such apparent ease *in vitro* [218]. Finally, it is worth reiterating that both *in vivo* and *in vitro* studies have shown that the tertiary allylic hydroperoxide **481** can be transformed into a wide variety of other compounds, in addition to artemisinin (**495**). Many of these compounds have been obtained previously as natural products from *A. annua* (see Scheme 5 and Scheme 6 in Section 2.6.3). Inspection of Scheme 12 suggests that some of these metabolites, such as dihydroarteannuin B (**479**) and dihydro-*epi-*deoxyarteannuin B (**485**) may be formed by alternative reactions to the Hock cleavage of **481** in step ii), while others such as the *seco*-cadinane **493** may arise by alternative reactions of the enol from Hock cleavage in step iii).

#### 3.3.2. Artemisinic Acid (**473**) as a Late-Stage Precursor to Artemisinin

[2-^14^C]-Mevalonic acid lactone has been shown to be incorporated into artemisinic acid (**473**) in *A. annua* using plantlet hydroponic and stem tip feeding methods [306]. Several experiments in the older literature (1990’s and earlier) have suggested that artemisinic acid (**473**) can then be converted in to both arteannuin B (**462**) and artemisinin (**495**) [215,281,285]. All these experiments employed radiolabelled forms of artemisinic acid that were presented to cell-free systems, which had been derived by extensive manipulations of *A. annua* plants. However, the most recent biosynthetic study [155], which employed stable isotope-labeled artemisinic acid [15-[^2^H_3_^13^C]-(**473**)] administered to a whole plant system, seemed to show that artemisinic acid was not a biosynthetic precursor to artemisinin. The experimental conditions for this study were purposely chosen to allow comparison with a previous investigation, which had demonstrated that dihydroartemisinic acid [15-[^2^H_3_^13^C]-(**480**)] was a precursor to artemisinin (**495**)) [185] (see discussion in a) above). In addition, it should be noted that a separate study with a cell-free system has also confirmed that dihydroartemisinic acid (**480**) is a precursor to artemisinin in *A. annua*, while artemisinic acid (**473**) is not [307]. 

**Scheme 13:**
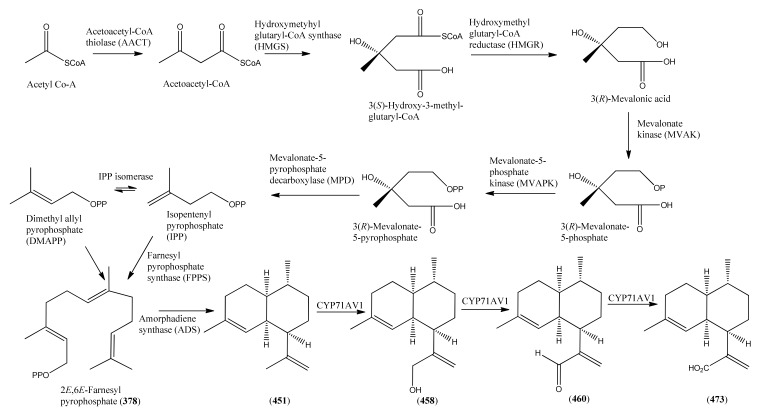
Genetic engineering of enzymes for the production of artemisinic acid (**473**) from *A. annua* in to a microbial host.

One explanation for these apparently contradictory results with artemisinic acid (**473**) may lie in the differing experimental approaches which have been adopted. The threshold for detection by NMR of a metabolite which is labeled with a stable isotope is likely to be several orders of magnitude higher than the corresponding threshold for a radioisotpic label. Thus, it is possible that the “older” experiments (1990s and earlier), which detected radiolabel, might - in reality - be identifying only trace quantities of metabolites, which were not derived directly from the labeled precursor. It is possible that partial degradation of a radio-labeled precursor can lead to some radioactivity appearing in a “pool” of small molecules, such as acetyl-CoA (see Scheme 13). These small molecules would then serve as precursors for several biosynthetic pathways, including the terpenoid pathway, and could thereby be incorporated indirectly into many metabolites from such pathways. By contrast, the stable isotope-labeled metabolites which have been detected by NMR [155] represented at least 1% (very often between 5-30%) of the label that had been supplied and therefore provide a picture only of the most significant and direct transformations which have been undergone by the precursor. In addition, it should also be pointed out that the possibilities for introducing artifacts when performing feeding studies with whole plants, which require no external manipulation, is likely to be significantly reduced as compared to cell-free extracts, which must endure many perturbations to the biological system (homogenization, addition of buffers *etc*..).

Although the most recent experiments with artemisinic acid (**473**) failed to observe any detectable incorporation into artemisinin, a very significant conversion was observed into arteannuin B (**462**), as well as six other highly oxygenated sesquiterpene natural products, all of which retained the 11,13- double bond, as shown in Scheme 7 in Section 2.6.3 [155] [furthermore, there was no evidence for conversion to any 11,13-dihydro metabolite, including artemisinin (**495**)]. Such transformations can most easily be accounted for by oxidation of the Δ^4,5^-double bond in **473** to a hydroperoxide, analogous to that postulated in Scheme 12 for dihydroartemisinic acid (**481**). There is indeed ample precedent for the formation of such hydroperoxides from **473** by photooxygenation reactions *in vitro* [243], which lead both to arteannuin B (**462**) [251] and 11,13-dehydro analogues of artemisinin [254,308].

In conclusion, although the absence for any detectable transformation of artemisinic acid into artemisinin in the most recent study [155] is at variance with the earlier literature (pre-1990’s) [281,284,286] for the biosynthesis of artemisinin, it is consistent with much of the more recent literature (post-1990’s), in which there is now a gathering consensus that dihydroartemisinic acid (**480**), rather than artemisinic acid (**473**), is the true late-stage precursor to artemisinin (**495**). 

#### 3.3.3. Arteannuin B (**462**) and Dihydroarteannuin B (**479**) as Late-Stage Precursors to Artemisinin

As noted above, in the most recent study of the metabolism of artemisinic acid, arteannuin B (**462**) was obtained as the major metabolite of artemisinic acid (**473**), without any evidence for the accompanying formation of artemisinin (**495**) [155]. However, because both arteannuin B (**462**) and artemisinin (**495**) were often reported together from “older” biosynthetic studies with artemisinic acid (**473**), some authors have previously proposed that arteannuin B (**462**) might be an intermediate in the proposed conversion of artemisinic acid (**473**) [215,251] to artemisinin (**495**) [203,248,285]. It is indeed possible to convert arteannuin B into artemisinin by chemical transformations [239] and there have been various suggestions as to mechanisms by which this transformation might also occur *in vivo* [209,217]. A microbial system which is capable of converting arteannuin B (**462**) to artemisinin (**495**) has recently been described [309] and an enzyme with the appropriate activity has also been purified [310,311]. On the other hand, a study which found that epoxyartemisinic acid (**487**) could be converted to arteannuin B (**462**) also stated that epoxyartemisinic acid (**487**) could not be transformed into artemisinin (**495**) and therefore – by implication – arteannuin B cannot be a precursor to artemisinin [284]. It has also been claimed that the 11,13-dihydro analogue of arteannuin B (**462**), dihydroarteannuin B (**479**), can be converted into artemisinin by cell free extracts of *A. annua* [249,312].

#### 3.3.4. epi-Deoxyarteannuin B (**478**) and Dihydro-epi-deoxyarteannuin B (**485**) as Late-Stage Precursors to Artemisinin

*epi*-Deoxyarteannuin B (**478**) was obtained as a minor metabolite of artemisinic acid (**473**), without any evidence for the accompanying formation of artemisinin (**495**), in the most recent study of the metabolism of artemisinic acid [155]. However, experiments in the “older” literature (1990’s and before), using radiolabelled forms of both *epi*-deoxyarteannuin B (**478**) and its 11,13-dihydro analogue, dihydro-*epi*-deoxyarteannuin B (**485**), have suggested that both were intermediates [286] in the respective biotransformations of artemisinic acid (**473**) and dihydroartemisinic acid (**480**) to artemisinin (**495**) [284]. However, once again, more recent experiments using dihydro-*epi*-deoxyarteannuin B (**485**) which had been labelled with a stable isotope [169] and fed to an intact plant system have shown no detectable incorporation into artemisinin (**495**); although three novel hydroxylated metabolites were obtained in significant quantities from this feeding experiment [190].

#### 3.3.5. The seco-Cadinane (**494**) and Artemisitene (**497**) as Late-Stage Precursosr to Artemisinin

The *seco*-cadinane aldehyde (**494**) has been hypothesized to be a precursor to artemisinin (**495**) via its enol tautomer, which undergoes reaction with molecular oxygen * to produce artemisitene (**497**), the 11,13-dehydro analogue of artemisinin (this reaction was proposed to occur in a similar manner to the transformations which have now been established experimentally for the enol in steps iii) and iv) in Scheme 12 [217,313]). Artemisitiene (**497**) can then reportedly be converted into artemisinin (**495**) *in vivo* [240] [the enol of **494** was proposed to be derived from Grob fragmentation of the vicinal diol **486**, which is in turn derived from arteannuin B (**462**)].

## 4. Strategies for the Production of Artemisinin from *A. annua* and Derived Systems

Although artemisinin can be produced by chemical synthesis [171,228,229,230,231,232,233,234,235,236,237,238,239], the structural complexity of this target and the large number of steps involved in all published synthetic routes render this approach far too expensive for many in the Third World who are most affected by malaria. Various alternative possibilities for the production of artemisinin from *A. annua* and derived systems have therefore been extensively investigated, and this topic has been amply reviewed [313,314,315,316,317,318,319,320]. Four strategies are considered in this review, all of which might benefit from a knowledge of the full biosynthetic pathway to artemisinin. These are: 1. Plant breeding programmes; 2. Plant tissue culture; 3. Endophytic fungi; and 4. Genetic engineering. The most successful strategy to date has been plant breeding, resulting in the cultivar “Artemis”, which was registered in Switzerland in 1999, and contains up to 1.4% artemisinin (by comparison, the extremely variable yields obtained from wild varieties of *A. annua* typically range between 0.01-0.5% artemisinin [321]). There is a potential for even higher yields in the future from on-going plant breeding programmes, such as that of the CNAP *Artemisia* project at the University of York (UK) [195]. 

Recent attempts to produce artemisinin through fermentation, by genetically engineering several enzymes from *A. annua* in to a microbial host, would seem to have the greatest promise for the cheap and reliable production of artemisinin. This approach, pioneered by the synthetic biologist Jay Keasling at UC Berkeley (USA), has so far yielded microbially-produced artemisinic acid (**473**), which is then transformed to artemisinin (**495**) in a separate chemical process [see Section 3.3b for a discussion of the confusion surrounding the true biosynthetic status of artemisinic acid (**473**)]. Amyris Biotechnologies and Sanofi-aventis are currently developing a commercial-scale manufacturing process, which is based on this semi-synthetic approach to artemisinin.

The discussions in Section 4.1, Section 4.2, Section 4.3 and Section 4.4 emphasize how a full understanding of both the phytochemistry of *A. annua* and the biosynthesis of artemisinin can be helpful for improving the production of this important antimalarial drug by each of these four strategies.

### 4.1. Plant Breeding Programmes

Plant selection and breeding programmes have often sought to combine the properties of artemisinin-rich clones of *A. annua* (such as the Chinese and Vietnamese varieties), with more vigorous but lower-yielding clones (such as some of the European varieties) in order to achieve a robust hybrid with a high yield of artemisinin (typically 1% or more – corresponding to an agricultural yield of approximately 200 Kg/ha for dry leaves) [322,323,324]. There is increasing evidence that artemisinin production in *A. annua* is localized in the biseriate glandular trichomes: specialized structures, found predominantly on the surface of leaves and flowers [244,325]. Several authors have reported that the artemisinin content is highest just before flowering [326,327,328] (interestingly, this trend is apparently not followed by other metabolites: the content of artemisia ketone (**276**) decreases before flowering and then increases afterwards; caryophyllene (**405**) shows the opposite trend; while levels of monoterpenes, such as cineole (**326**), bornyl acetate (**335**) and camphor (**341**) all remain relatively constant throughout growth [112]) and it has also been noted that the content of artemisinin can be up to ten-fold higher in flowers, as compared with leaves [19,329,330]. These observations may simply reflect an increased density of glandular trichomes in the flowers [301], and hence a correspondingly higher yield of artemisinin. By contrast, artemisinic acid (**473**) seems to be obtained in maximum yield at the late vegetative stage (however, the concentration of artemisinic acid in *A. annua* (**473**) is strongly dependant on chemotype – it can be up to ten-fold higher than that of artemisinin in European and Chinese varieties, although this is reversed in Vietnamese strains).

It has also been observed that increased illumination or sunlight results in a higher yield of artemisinin [331,332,333]. This would be consistent with the hypothesis that the final steps in the biosynthesis of artemisinin proceed in the trichomes by spontaneous autoxidation reactions of dihydroartemisinic acid (**480**), requiring ^1^O_2_, which is produced in the presence of light and a photosensitizer (e.g., chlorophyll – see step i) in Scheme 12). Several researchers have endeavoured to quantify not just the amount of artemisinin (**495**) [334], but also its presumed biosynthetic precursors, dihydroartemisinic acid (**480**) [335] and artemisinic acid (**473**) [335,336,337], when studying the developmental biology of *A. annua*. One particularly interesting study has concluded that correlated variations in all three natural products prove the existence of chemotypes of *A. annua*. Thus, plants with a high artemisinin (**495**) level were found also to have a high dihydroartemisinic acid (**480**) level, but a relatively low concentration of artemisinic acid (**473**); while chemotypes with low levels of artemisinin (**495**) and dihydroartemisinic acid (**480**) contained a correspondingly high concentration of artemisinic acid (**473**) [338]. These findings were explained [20] on the assumption that the enzymatic reduction of artemisinic acid (**473**) to dihydroartemisinic acid (**480**) might represent a "bottle neck" in the biosynthetic pathway to artemisinin (**495**). However, given that it seems now to be proven that artemisinic acid (**473**) is not a precursor to dihydroartemisinic acid (**480**) (see crossed arrow in Scheme 11) [155,185], the existence of these two chemotypes might instead be explained on the assumption that artemisinic acid (**473**) is a “dead end” metabolite, leading “away” from artemisinin (**495**) in phase 2 of the biosynthetic route, as is implied in Scheme 11.

Finally, there have been several recent attempts to create transgenic *A. annua* plants with elevated levels of artemisinin [339,340,341] by transferring genes for various enzymes in the biosynthetic pathway to artemisinin (e.g. HMGR, FPPS, ADS and CYP71AV1 – see Scheme 13). 

### 4.2. Plant Tissue Culture

**Figure 4:**
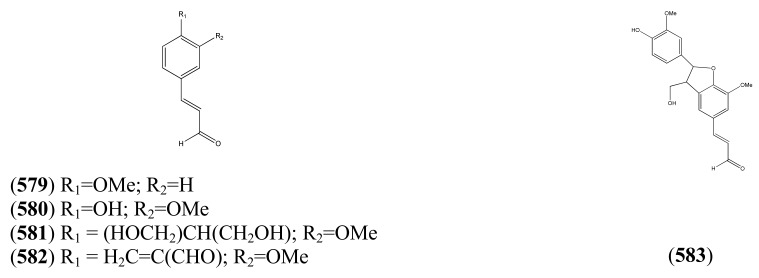
Metabolites which have been isolated from tissue culture of *A.annua*, but which have not yet been reported from *A. annua* plants.

The production of artemisinin from plant tissue cultures of *A. annua* has been reviewed [342]. Although artemisinin can be obtained in reasonable quantities from differentiated shoot cultures [343,344,345], it is produced in only small or undetectable amounts [346,347,348] by callus or suspension cell cultures of *A. annua*. Instead, the chemistry of undifferentiated callus and suspension cultures of *A. annua* seems to be dominated by sterols, such as stigmasterol (**570**) and sitosterol (**568**) (Section 2.7.2) [86,313]; as well as phenylpropanoids, such as scopoletin (**184**) (Section 2.3; Table 17), all of which are also found in significant quantities in the parent plants [86,313]. Other phenylpropanoids, such as 4-methoxycinnamaldehyde (**579**), coniferaldehyde (**580**), (2-glyceryl)-*O*-coniferaldehyde (**581**) [87], (2-propenal)-*O*-coniferaldehyde (**582**) [87] and balanophonin (**583**) [87] are not yet known from *A. annua* plants themselves (Figure 4). The triglyceride, glycerol 1,2-di-9-octadecenoate 3-octadecanoate (**584**), with some structural similarities to 9-octadecenoic acid, 2,3-dihydroxypropyl ester (**82**) (Table 5), has also been obtained from plant tissue culture [86]. These major alterations observed in the metabolism of undifferentiated callus and suspension cultures may reflect the absence of glandular trichomes, in which much of the biosynthesis of the mono- and sesquiterpenoids which are described in Section 2.5 and Section 2.6 is thought to occur. 

Much of the current research activity into the production of artemisinin by plant tissue culture revolves around hairy root cultures of *A. annua* [349] which have been transformed by *Agrobacterium tumefaciens* [350,351] (elicitors from endophytic fungi – see the next section – have also been employed in *A. annua* plant tissue cultures [202,352,353]). Correlations have been observed between peroxidase activity and artemisinin levels in hairy roots [354], although it has also been suggested that a high peroxidase activity in cell cultures may be partly responsible for their very low artemisinin contents observed [355].

### 4.3. Endophytic fungi

Endophytic fungi live symbiotically within the tissues of many plants. Interest in endophytic fungi as a novel source of plant-derived drugs was first sparked by the observation of the lateral transfer of genes which are involved in the biosynthesis of taxol (paclitaxel) from *Taxus brevifolium* L. [356] - the plant which produces this important anti-cancer compound - to an endophytic symbiont. Thus, if it were found that some of the genes for the biosynthetic pathway to artemisinin in *A. annua* had been “naturally” transferred to an endophyte which inhabited this species, then it might be possible to utilize that endophyte for the production of artemisnin in a fermentation process. Several endophytes have now been described from *A. annua* [357]. While chemical studies of such endophytic fungi have demonstrated a fascinating variety of natural product chemistry (see Table 44), only one strain is so far reported as a potential source of artemisinin [358].

**Table 44:** Natural Products from Endophytic Fungi Found in *A. annua.*

Endophyte	Compound	Reference
*Myrothecium roridum* (IFB-E009; IFB-E012)	Myrothecine A (**585**)	[359]
	Myrothecine B (**586**)	[359]
	Myrothecine C (**587**)	[359]
*Hypoxylon truncatum* (IFB-18)	Daldinone C (**588**)	[360]
	Daldinone D (**589**)	[360]
	Altechromone A (**590**)	[360]
	(4*S*)-5,8-Dihydroxy-4-methoxy-α-tetralone (**591**)	[360]
*Leptosphaeria sp.* (strain number IV403)	Leptosphaeric acid (**592**)	[361]
	Leptosphaerone (**593**)	[362]
*Colletotrichum sp.*	3β-Hydroxy-ergosta-5-ene (**594**)	[363]
	Ergosterol (**595**)	[363]
	3β,5α,6β-Trihydroxyergosta-7,22-diene (**596**)	[363]
	3β,5α-Dihydroxy-6β-acetoxy-ergosta-7,22-diene (**597**)	[363]
	3β,5α-Dihydroxy-6β-phenylacetyloxy-ergosta-7,22-diene (**598**)	[363]
	3β-Hydroxy-5α,8α-epidioxy-ergosta-6,22-diene (**599**)	[363]
	3β-Hydroxy-5α,8α-epidioxy-ergosta-6,9(11),22-triene (**600**)	[363]
	3-Oxo-ergosta-4-ene (**601**)	[363]
	3-Oxo-ergosta-4,6,8(14),22-tetraene (**602**)	[363]
	Indole-3-acetic acid (**603**)	[363]
	6-Isoprenylindole-3-carboxylic acid (**604**)	[363]

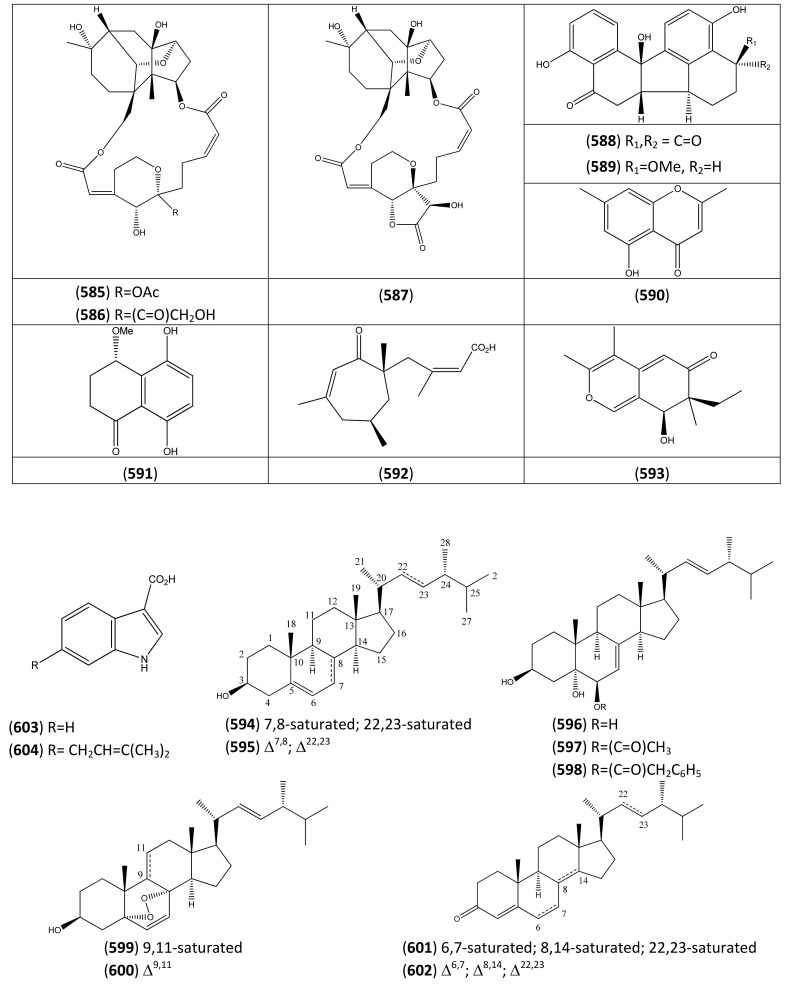

### 4.4. Genetic Engineering

Metabolic engineering has attracted increasing attention over recent years as an alternative means for the production of plant-derived drugs. Both artemisnin from *A. annua* and taxol from *Taxus brevifolium* are attractive targets for this emerging science of synthetic biology since both drugs are available in only limited quantities from the natural source and are also difficult to synthesize chemically [364]. Research into the production of artemisinin from such genetically modified microorganisms has been well reviewed [365,366,367,368,369].

The goal of synthetic biology is to reprogram a microorganism for the efficient production of a natural product by establishing new metabolic pathways, which lead to the formation of the desired metabolite, whilst simultaneously removing existing metabolic pathways which detract from the formation of such a product. To date, investigators have concentrated on expressing the enzymes from phases 1 and 2 of the biosynthetic route to artemisinin in transgenic organisms (see Scheme 9 in Section 3). These have included: HMG-CoA reductase (HMGR) [370]; farnesyl diphosphate synthase (FPPS) [371, 372,373,374]; amorpha-4,11-diene synthase (ADS) [375,376,377]; and the P_450_ enzyme which oxidises amorpha-4,11-diene to artemisinic acid (CYP71AV1) [378] (Scheme 13). Both *Saccharomyces cerevisiae* [379,380] and *Escherichia coli* [381,382,383] have been used as hosts when attempting to establish a viable recombinant microbial pathway for the biosynthesis of artemisinin.

The approach outlined in Scheme 13 has succeded in producing high levels of amorpha-4,11-diene (**451**) from *E. coli* [384] and artemisinic acid (**473**) from *S. cerevisiae* in quantities which exceed those from *A. annua* itself (100 mg^–^^1^·g·L^-1^) [385,386]. The semi-synthesis of artemisinin (**495**) from microbially-produced artemisinic acid (**473**) then requires two further chemical steps: reduction of the exocyclic double bond; and photo-sensitized oxidation of the endocyclic double bond to produce the 1,2,4-trioxane ring in (**495**) [156,237]. Fortunately, the genetically-engineered yeast transports artemisinic acid (**473**) outside of the cell, where it is retained on the cell wall, and can easily be released simply by altering the pH. It is then necessary to purify microbially-produced artemisinic acid (**473**) prior to these chemical transformations.

In order to achieve the heterologous production of artemisinin (**495**) by a large-scale fermentation process, it will be necessary to express every step in the biosynthetic pathway to artemisinin in a microbe. Keasling has observed that “the production of drugs *via* heterologous pathways in microbial hosts is frequently hindered by insufficient knowledge of the native metabolic pathway and its cognate enzymes; often the pathway is unresolved, and the enzymes lack detailed characterization” [387]. These comments are certainly pertinent to the biosynthesis of artemisinin in *A. annua*, as is evident from the foregoing discussions in Section 3.2 and 3.3. The author would argue that the prerequisite for the reconstruction of the complete biosynthetic pathway to artemisinin (**495**) in a transgenic yeast or bacterium is the elucidation of the full details of phase 2 of the biosynthesis in *A. annua*, in which amorpha-4,11-diene (**451**) is converted to dihydroartemisinic acid (**480**); as well as a better understanding of phase 3, in which **480** is then converted to **495** (see Scheme 9). If this can be achieved, then it should - at the very least - be possible to produce dihydroartemisinic acid (**480**), rather than artemisinic acid (**473**), by the fermentation of simple sugars (in fact, the production of dihydroartemisinic acid (**480**) in yeast using the Dbr2 gene has now been described [298] – see also discussion at the end of Section 3.2 – and the use of substrate-promiscuous enzymes as an alternative means for the microbial production of (**480**) is also under investigation [387]). 

If the final stages in the conversion of dihydroartemisinic acid (**480**) to the 1,2,4-trioxane ring in artemisinin (**495**) (phase 3 in Section 3.3) do turn out to be enzymatically catalysed, then it may indeed be possible to genetically engineer all the enzymes for the complete biosynthetic pathway to artemisinin (**495**) into a microbial system. The current evidence, however, points to the final steps in the biosynthesis of artemisinin (**495**) being non-enzymatic – most probably proceeding by spontaneous autoxidation reactions, which occur in the hydrophobic environment of a glandular trichome. If this is the case, then the simplest approach might be to engineer a microorganism to produce dihydroartemisinic acid (**480**) which is exported out of the cell, where it can more easily be isolated and purified [in much the same way that genetically-engineered yeast accumulates artemisinic acid (**473**) on the cell surface]. Inevitably, a chemical oxidation step would still then be required in order to convert microbially-produced dihydroartemisinic acid (**480**) to artemisinin (**495**), but this would certainly represent a saving in time and cost by comparison with the two-step chemical transformation (both reduction and oxidation), which is required for microbially-produced artemisinic acid (**473**). 

An alternative more radical approach would be to attempt to mimic within the microbial fermentation medium the spontaneous autoxidation reactions of dihydroartemisinic acid (**480**) which are believed to occur in the glandular trichomes of *A. annua* plants. This would require the inclusion of some kind of hydrophobic phase in intimate association with the fermentation medium. Previously, dodecane has been incorporated into the culture medium, in order to provide a separate hydrophobic layer in which volatile amorpha-4,11-diene (**451**) produced by microbial fermentation can be trapped [382, 385]. It would be interesting to investigate whether alternative hydrophobic phases can be found which provide a lipophilic environment similar to that of the glandular trichome, in which spontaneous autoxidation is favoured. (Such a phase would also need to be able to efficiently “trap” dihydroartemisinic acid and to be non-toxic to the fermenting organism). It should be noted that our understanding of how the spontaneous autoxidation of natural products such as **480** occurs in the absence of a photosensitizer [388] (or, indeed, any other additional chemical reagents [218]), leading to highly oxidized products such as **495**, is still incomplete. This is a relatively unexplored area which requires fundamental research, but the ultimate prize would be the production of artemisinin, both cheaply and reliably, by a single fermentation process, which incorporates an “in-built” spontaneous autoxidation step.
